# MYCL promotes iPSC-like colony formation via MYC Box 0 and 2 domains

**DOI:** 10.1038/s41598-021-03260-5

**Published:** 2021-12-20

**Authors:** Chiaki Akifuji, Mio Iwasaki, Yuka Kawahara, Chiho Sakurai, Yu-Shen Cheng, Takahiko Imai, Masato Nakagawa

**Affiliations:** Department of Life Science Frontiers, Center for iPS Cell Research and Application (CiRA), Kyoto University, Kyoto, 606-8507 Japan

**Keywords:** Stem cells, Reprogramming

## Abstract

Human induced pluripotent stem cells (hiPSCs) can differentiate into cells of the three germ layers and are promising cell sources for regenerative medicine therapies. However, current protocols generate hiPSCs with low efficiency, and the generated iPSCs have variable differentiation capacity among different clones. Our previous study reported that MYC proteins (c-MYC and MYCL) are essential for reprogramming and germline transmission but that MYCL can generate hiPSC colonies more efficiently than c-MYC. The molecular underpinnings for the different reprogramming efficiencies between c-MYC and MYCL, however, are unknown. In this study, we found that MYC Box 0 (MB0) and MB2, two functional domains conserved in the MYC protein family, contribute to the phenotypic differences and promote hiPSC generation in MYCL-induced reprogramming. Proteome analyses suggested that in MYCL-induced reprogramming, cell adhesion-related cytoskeletal proteins are regulated by the MB0 domain, while the MB2 domain regulates RNA processes. These findings provide a molecular explanation for why MYCL has higher reprogramming efficiency than c-MYC.

## Introduction

Human induced pluripotent stem cells (hiPSCs) are generated from somatic cells and can differentiate into cells of all three germ layers^1,2^. They are functionally identical to human embryonic stem cells (hESCs) but do not require the destruction of the embryo, which has made them attractive sources for regenerative medicine^3^. The original reprogramming was induced by four factors, OCT3/4, SOX2, KLF4, and c-MYC (OSKM). Since then, several new methods have been developed to improve the yield and quality of iPSCs, but the cost remains high and the production remains technically difficult^4,5^. Further complicating the application of hiPSCs is the wide variability in the differentiation capacity of different hiPSC clones^6^.

We have shown that excluding c-MYC from the reprogramming factors significantly lowers the reprogramming and differentiation efficiencies of the resulting iPSCs^7^. The MYC family consists of the oncogenes c-MYC, MYCN, and MYCL in humans^8^. c-MYC was the first MYC gene discovered in human and has been a topic of cancer research ever since^9^. Tumorigenesis depends on high transformation activity derived from the N-terminus region of c-MYC protein^10^. Consequently, OSKM-based reprogramming may not be appropriate for the clinical application of iPSCs. Many groups have reported reprogramming methods that exclude c-MYC overexpression but at the cost of lower reprogramming efficiency^5,7^. MYCL is about 30 amino acids shorter in the N-terminus region than c-MYC and has lower transformation activity^10^. We found that substituting c-MYC for MYCL in reprogramming can increase the number of iPSC colonies and maintain the ability to differentiate into the cells of three germ layers^7^. Furthermore, fewer chimeric mice died by tumorigenesis after the transplantation of MYCL-iPSCs, whereas the transplantation of c-MYC-iPSCs caused lethal tumorigenesis in more than 50% of mice during two years of observation. Despite these observations, little is known about the molecular function of MYCL and the different mechanisms between c-MYC and MYCL to promote reprogramming.

MYC proteins have six MYC Box (MB) domains: MB0, 1, 2, 3a, 3b, and 4 in the N-terminus and a basic helix-loop-helix leucine zipper (bHLHLZ) in the C-terminus^11^, but MYCL does not have MB3a. The C-terminus of c-MYC and MYCL is essential in reprogramming due to its binding with MAX protein, allowing MYC to access the DNA^7,12^. The N-terminus is mainly known as a transactivation domain (TAD), which regulates the target gene, but its function in reprogramming is less clear^13^. We found that a mutant of c-MYC lacking the N-terminal showed low transformation activity and promoted reprogramming^7^. However, which domain on the N-terminal side is essential for reprogramming and what function it performs were not resolved. In addition, MYC proteins act as transcription factors upon interacting with several binding proteins^14^. Although MYCL-binding proteins are important for MYCL function, there are no reports about MYCL-binding proteins during reprogramming.

In this study, using domain deletion mutants of MYC proteins, we found that the MB0 and MB2 domains promote iPSC-like colonies and that the MB0 domain is functionally different between c-MYC and MYCL. In c-MYC, it induced non-iPSC-like colonies by increasing nucleic proteins related to transcription, but in MYCL, the MB0 domain induced iPSC-like colonies by increasing the expression of cell adhesion-related proteins. We also found that deletion of the MB2 domain in MYC proteins prevented colony formation and that MYCL could interact with RNA-binding proteins (RBPs) via this domain. These results suggested that MYCL promotes reprogramming by regulating RNA processing.

## Results

### MYCL promotes reprogramming more efficiently than c-MYC

**Figure 1:**
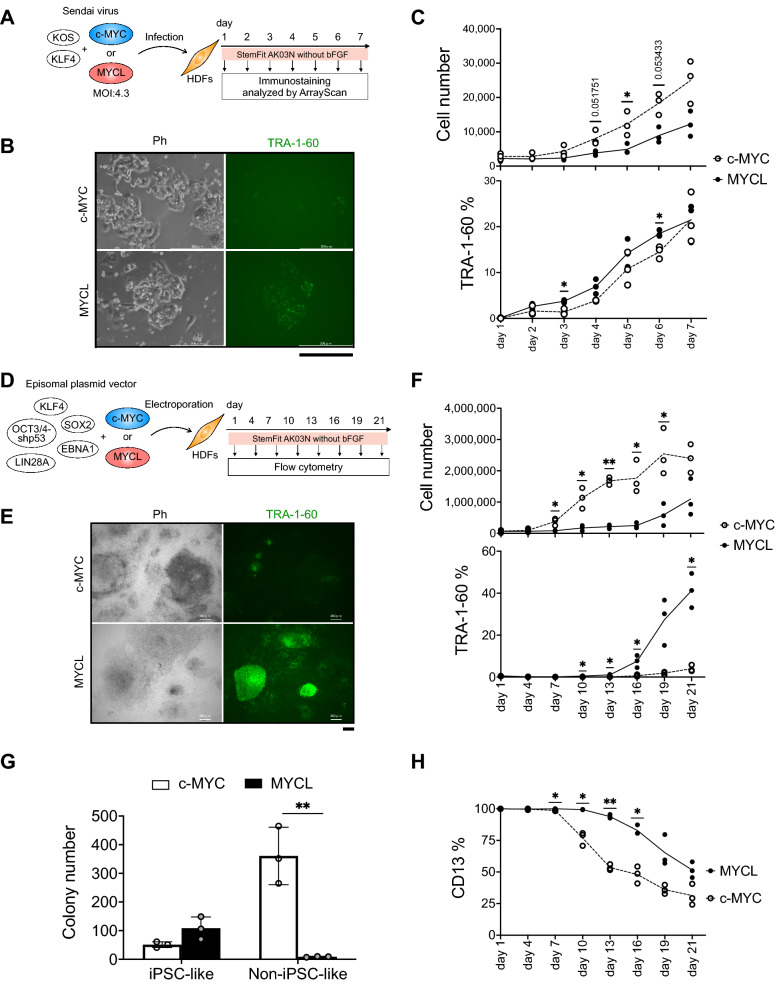
MYCL promotes reprogramming more efficiently than c-MYC. (**A**) Schematic representation of HDF reprogramming with Sendai virus (SeV). HDFs were transduced with SeV carrying KLF4-OCT3/4-SOX2 (KOS), KLF4 (K), and c-MYC or MYCL on day 0. We used an MOI (multiplicity of infection) of 4.3 for each virus. StemFit AK03N without bFGF was used during the transduction and subsequent induction of iPSC-like colonies. We performed immunostaining of the reprogramming HDFs 1 to 7 days after the transduction and analyzed the results using ArrayScan. (**B**) Representative immunostaining images of reprogramming HDFs stained by anti-TRA-1-60 antibody (green) and Hoechst (blue) 7 days after the transduction. Scale bar, 300 μm. Ph, phase contrast. (**C**) Proliferation and expression of TRA-1-60 ( +) cells during reprogramming. HDFs were transduced with SeV, including c-MYC or MYCL, and immunostaining was performed from days 1 to 7. The number of total cells was counted as Hoechst-positive cells. Mean ± SD values are shown. *n* = 3, *p < 0.05 by paired *t*-test. (**D**) Schematic representation of HDF reprogramming with episomal plasmid vector (EpiP). HDFs were transduced with EpiP carrying SOX2, KLF4, OCT3/4-shp53, LIN28A, EBNA1, and c-MYC or MYCL. StemFit AK03N without bFGF was used during the transfection and subsequent induction of iPSC-like colonies. We performed flow cytometry of the reprogramming HDFs every three days from 1 to 19 days plus day 21 after the transduction. (**E**) Representative immunostaining images of reprogramming HDFs stained by anti-TRA-1-60 antibody (green) and Hoechst (blue) 21 days after the transduction. Scale bar, 300 μm. Ph, phase contrast. (**F**) Proliferation and expression of TRA-1-60 ( +) cells during reprogramming were analyzed by flow cytometry. HDFs were transduced with EpiP, including c-MYC or MYCL. Flow cytometry was performed every three days from days 1 to 19 days plus day 21. Mean ± SD for *n* = 3, *p < 0.05 and **p < 0.01 by paired *t*-test. (**G**) The number of iPSC-like and non-iPSC-like colonies derived from 1 × 10^5^ HDFs transduced with EpiP including c-MYC or MYCL on day 21. Mean ± SD values are shown. *n* = 3, **p < 0.01 by unpaired *t*-test. (**H**) Percentage of CD13 ( +) cells during EpiP reprogramming determined by flow cytometry. Mean ± SD values are shown. *n* = 3, *p < 0.05 and **p < 0.01 by paired *t*-test.

To compare the reprogramming phenotypes of MYCL and c-MYC, we used Sendai virus (SeV)-based reprogramming (CytoTune-iPS) and StemFit AK03N medium without bFGF (Fig. 1A). The SeV method has high reprogramming efficiency without genome integration, and c-MYC and MYCL SeV kits are already available^15^. The bFGF exclusion is based on the data in Supplementary Fig. S1. DMEM supplemented with 10% FBS (DMEM + 10%FBS) is the standard medium to induce reprogramming. We used DMEM + 10%FBS when introducing the reprogramming factors, but after 7 days of reprogramming, we replated the cells and used StemFit AK03N without bFGF (03N (-)) from that point on. The MOI (multiplicity of infection) of each SeV was 20. To improve the reprogramming efficiency, we compared three media combinations (Supplementary Fig. S1A). The highest number of colonies was obtained using 03N (-) during reprogramming and 03N ( +) after replating (Supplementary Fig. S1B). These results indicated that the 03N (-) reprogramming condition in the first 7 days enhances the reprogramming efficiency compared to 03N ( +). We then examined the optimal MOI of SeV for the reprogramming (Supplementary Fig. S1C). A lower MOI induced more colonies (Supplementary Fig. S1D), indicating a higher reprogramming efficiency. Following these results, we applied SeV for the transduction at an MOI of 4.3 using 03N (-) during reprogramming.

Next, we conducted immunostaining to analyze the expression of TRA-1-60 from days 1 to 7 after the transduction (Fig. 1A). TRA-1-60 is a glycoprotein and major cell surface marker of hiPSCs and hESCs^16^. We quantified the results using a high-content imaging system, ArrayScan, because the cell number was small during SeV reprogramming for the first seven days, making flow cytometry challenging. On day 7, we observed that c-MYC and MYCL induced a small cell mass to form colonies, but only the colonies induced by MYCL expressed TRA-1-60, while those induced by c-MYC looked like cell aggregations (Fig. 1B and Supplementary Fig. S2). Cell proliferation was highly increased in human dermal fibroblasts (HDFs) transduced with c-MYC compared to MYCL. On the other hand, the percentage of TRA-1-60 ( +) cells increased more in MYCL-transduced HDFs on day 3 after the transduction (Fig. 1C). This difference may be because c-MYC has higher transformation activity than MYCL, which causes different phenotypes, especially cell proliferation^10^.

We confirmed these reprogramming phenotypes using episomal plasmid vector (EpiP)^17^ (Fig. 1D). SeV systems have a higher gene transfer efficiency, leading to more efficient reprogramming. However, we could modify the reprogramming vectors, which is useful for evaluating the molecular mechanism of c-MYC and MYCL, only when using the EpiP system.

Similar to the results with the SeV method, few colonies expressed TRA-1-60 in c-MYC-transfected HDFs (Fig. 1E and Supplementary Fig. S3A). However, the transfection of MYCL resulted in a higher percentage of TRA-1-60 ( +) cells and lower cell proliferation than the transfection of c-MYC (Fig. 1F and Supplementary Fig. S3B). These differences between MYCL and c-MYC were more obvious with EpiP reprogramming than SeV reprogramming (Fig. 1C, F), probably because of differences in the gene transfer efficiency^15,17^, the expression of the transfected factors, cell toxicity, and the time required for the iPSC-like colonies to appear: the SeV system requires about 7 days, but the EpiP system needs about 21 days based on our observations.

We found two types of colonies: “iPSC-like” and “non-iPSC-like” colonies. The iPSC-like colonies produced by MYCL were more flattened and showed a monolayered colony morphology, with each cell tightly packed and expressing TRA-1-60. The non-iPSC-like colonies produced by c-MYC showed a cell aggregation-like morphology, in which individual cells were irregularly aggregated and did not express TRA-1-60. We counted the number of iPSC-like and non-iPSC-like colonies on day 21 and found that c-MYC induced iPSC-like colonies as well as many non-iPSC-like colonies, but MYCL induced almost only iPSC-like colonies and more of them than c-MYC (Fig. 1G and Supplementary Fig. S3).

It has been reported that before the increase in the expression of TRA-1-60, a decrease in the expression of CD13, a marker of fibroblasts^18^, is observed in somatic cell reprogramming. Therefore, we confirmed the expression of CD13 during reprogramming. The percentage of CD13 ( +) cells decreased daily in HDFs transduced with c-MYC or MYCL, but the number of CD13 (-) cells rapidly increased in c-MYC compared to MYCL (Fig. 1H and Supplementary Fig. S4). In particular, the CD13 (-) TRA-1-60 (-) population was larger on day 10 with c-MYC reprogramming than MYCL reprogramming, but the CD13 (-) TRA-1-60 ( +) population from days 16 to 21 was larger with MYCL reprogramming (Supplementary Fig. S4). These results suggested that MYCL promotes TRA-1-60 ( +) cells more than c-MYC, but c-MYC suppresses CD13 expression more than MYCL.

### MYC Box 0 and 2 domains are crucial for colony formation during reprogramming

**Figure 2:**
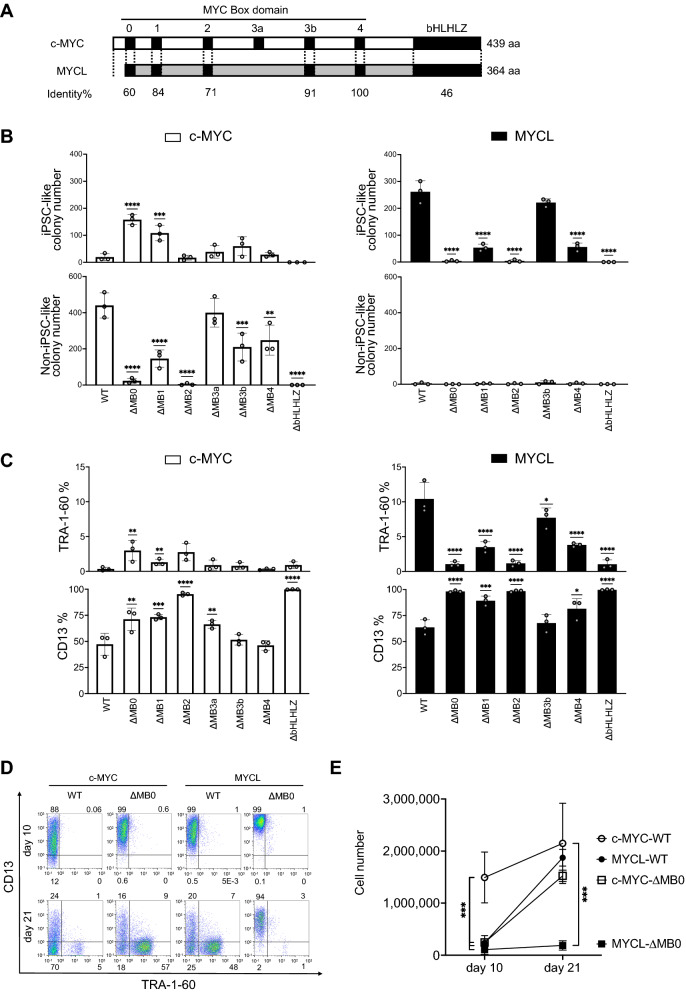
MYC Box 0 and 2 domains are crucial for colony formation during reprogramming. (**A**) Schematic representation of WT c-MYC and MYCL protein. Black boxes show important domains for MYC function, including MB0, 1, 2, 3a (c-MYC only), 3b, 4, and basic-helix-loop-helix leucine zipper motif (bHLHLZ). The percentage of common amino acids in each MYC box domain between MYCL and c-MYC is shown (Identity%). The numbers on the right indicate amino acid lengths. (**B**) Number of iPSC-like and non-iPSC-like colonies transduced with EpiP including c-MYC-WT/mutants (left) or MYCL-WT/mutants (right) on day 21. Mean ± SD values are shown. *n* = 3, **p < 0.01, ***p < 0.001 and ****p < 0.0001 by ordinary one-way ANOVA and Dunnett’s test vs. WT. (**C**) Expression of TRA-1-60 ( +) HDFs and CD13 ( +) HDFs transduced with EpiP including c-MYC-WT/mutants (left) or MYCL-WT/mutants (right) on day 16. Mean ± SD values are shown. *n* = 3, *p < 0.05, **p < 0.01, ***p < 0.001 and ****p < 0.0001 by ordinary one-way ANOVA and Dunnett’s test vs. WT. (**D**) Representative flow cytometry images for TRA-1-60 and CD13 for HDFs transduced with EpiP including c-MYC-WT/ΔMB0 or MYCL-WT/ΔMB0 10 and 21 days after the transduction. Numbers indicate the expression percentage of each quadrant. (**E**) Proliferation of HDFs transduced with EpiP including c-MYC-WT/ΔMB0 or MYCL-WT/ΔMB0 10 and 21 days later. Mean ± SD values are shown. *n* = 3, ***p < 0.001 by ordinary one-way ANOVA and Dunnett’s test vs. c-MYC-WT. The number of cells was counted using a Cell Counter model R1 (OLYMPUS).

Next, we prepared domain deletion mutants to identify which domains in the N-terminus of MYC proteins influence reprogramming (Fig. 2A and Supplementary Fig. S5). We previously showed that a c-MYC mutant lacking transformation activity enhances the formation of iPSC-like colonies. This mutant has a point mutation in the transactivation domain of the N-terminal region, W135E (Fig. 2A and Supplementary Fig. S5B), but can bind to genomic DNA^7^. On the other hand, the bHLHLZ domain in the C-terminus region is a well-known binding domain of MAX^19^. Mutants in the C-terminus region prevent MYC proteins from binding to DNA and thus reprogramming^7^. Finally, we tested the reprogramming activities of these mutants using the EpiP reprogramming system because, as explained above, this method provided a clearer phenotype and was easier to manipulate than the SeV method.

The EpiP mutants were transfected into HDFs with other reprogramming factors, and the number of iPSC-like and non-iPSC-like colonies was counted (Fig. 2B and Supplementary Fig. S6). c-MYC-ΔMB0 promoted the formation of iPSC-like colonies and inhibited the formation of non-iPSC-like colonies compared to c-MYC-WT. In contrast, MYCL-ΔMB0 showed almost no ability to form iPSC-like colonies (Fig. 2B). We confirmed that the protein expression of each domain deletion mutant by western blotting showed no difference compared with c-MYC- or MYCL-WT (Supplementary Fig. S7 and S8). These results demonstrate that the MB0 domain has different functions in c-MYC and MYCL for reprogramming and that c-MYC-ΔMB0 has a similar function as MYCL-WT.

Figure 2B shows that c-MYC-ΔMB1 promoted iPSC-like colony formation like c-MYC-ΔMB0, but it also led to the formation of non-iPSC-like colonies. The formation of iPSC-like colonies by MYCL-ΔMB1 was about a quarter that by MYCL-WT. Unlike c-MYC-WT, c-MYC-ΔMB2 did not induce non-iPSC-like colonies, but it did induce a rate of iPSC-like colonies similar to c-MYC-WT. MYCL-ΔMB2 showed little ability to form iPSC-like colonies, resembling MYCL-ΔMB0. c-MYC-ΔMB3a, -ΔMB3b, and -ΔMB4 had similar colony-forming activities as c-MYC-WT. MYCL-ΔMB3b showed the same reprogramming efficiency as MYCL-WT, but MYCL-ΔMB4 formed about the same small number of iPSC-like colonies as MYCL-ΔMB1. The ΔbHLHLZ mutants of both c-MYC and MYCL failed to induce colonies and were therefore considered to have lost MYC function completely. Thus, the results indicate that in c-MYC, the MB0 and MB2 domains are repressive for iPSC-like colony formation, but in MYCL, they are promotive. Other domains also influenced the colony formation efficiency, but the effect was small.

Next, we analyzed the effect of the MYC-deletion mutants on the expression of TRA-1-60 and CD13 by flow cytometry 16 days after the start of reprogramming (Fig. 2C). Mutants that increased the number of iPSC-like colonies also increased the expression of TRA-1-60, while those that reduced the number of iPSC-like colonies lowered the TRA-1-60 expression (Fig. 2C and Supplementary Fig. S9). c-MYC-WT showed little TRA-1-60 expression, whereas c-MYC-ΔMB0 upregulated the expression. MYCL-ΔMB0, unlike MYCL-WT, failed to upregulate the expression of TRA-1-60. The CD13 expression was also correlated with colony formation. In c-MYC, a significant decrease in CD13 expression was observed for mutants that promoted non-iPSC-like colony formation. As for MYCL, only a slight decrease in CD13 expression was observed for mutants that promoted iPSC-like colony formation. From these results, we concluded that the MB0 domain is essential for the function of MYC in reprogramming but functions differently between c-MYC and MYCL.

To analyze the function of the MB0 domain in more detail, we analyzed the expression of TRA-1-60 and CD13 10 and 21 days after the start of reprogramming by flow cytometry (Fig. 2D). In the case of c-MYC-WT, there was a strong decrease in CD13 expression on day 10, and most cells were CD13 negative on day 21. In the cases of c-MYC-ΔMB0 and MYCL-WT, there was a slight decrease in CD13 expression on day 10, and more than half of cells were expressing TRA-1-60 on day 21. Finally, in the case of MYCL-ΔMB0, there was no change in CD13 or TRA-1-60 expression. More study is needed to determine how CD13 is regulated by c-MYC and MYCL.

Additionally, c-MYC-WT showed higher cell proliferation on day 10, but c-MYC-ΔMB0 resulted in a lower cell proliferation comparable more with MYCL-WT than with c-MYC-WT on day 10 (Fig. 2E). We attributed this effect to the lost transformation activity of c-MYC-ΔMB0. From days 10 to 21, the cell proliferation increased significantly in c-MYC-ΔMB0 and MYCL-WT, and a concomitant increase in the CD13 (-) TRA-1-60 ( +) population was observed (Fig. 2D, E). These observations suggest that the number of cells that were reprogrammed increased rapidly with c-MYC-ΔMB0 and MYCL-WT. With c-MYC-WT, the cell proliferation continued until day 21. However, the CD13 (-) TRA-1-60 ( +) population hardly increased (Fig. 2D), indicating that these cells were not reprogramming but changing to other highly proliferative cell types. From these results, we concluded that the MB0 domain functions negatively in c-MYC and positively in MYCL for reprogramming.

### MYCL regulates cytoskeleton- and cell adhesion-related proteins during reprogramming via the MB0 domain

**Figure 3:**
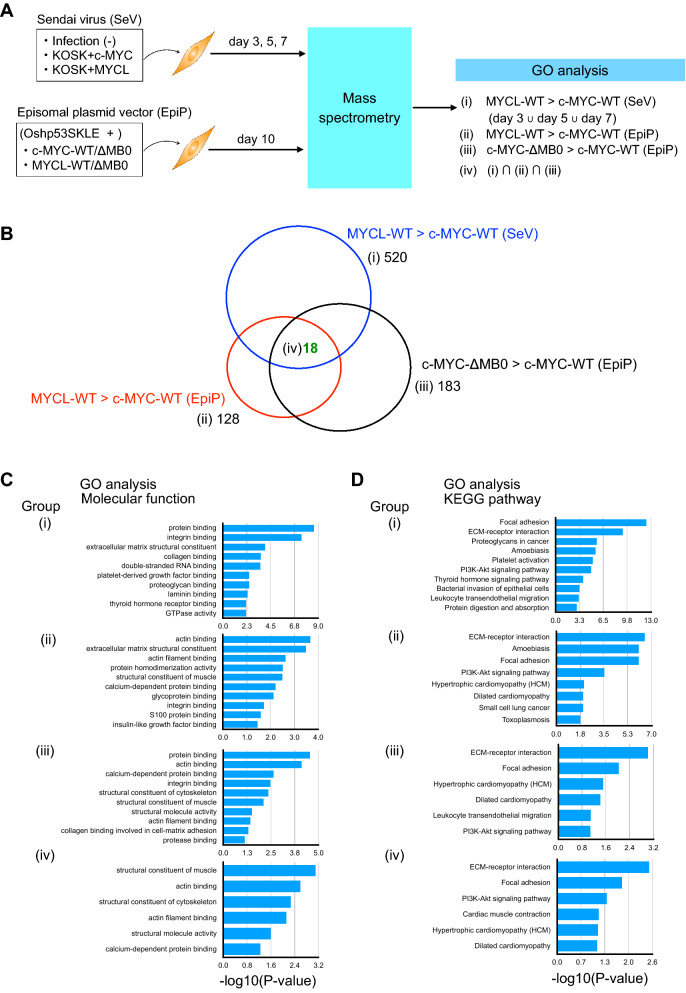
MYCL regulates cytoskeleton- and cell adhesion-related proteins during reprogramming via the MB0 domain. (**A**) Schematic of the mass spectrometry (MS) and GO analysis (DAVID). (**B**) Venn diagram of upregulated proteins during iPSC-like colony formation. (**C**) Molecular functions from the GO analysis of the four groups in (**B**). (**D**) KEGG pathways from the GO analysis of the four groups in (**B**).

To confirm which genes are regulated by the MYCL MB0 domain in reprogramming, we analyzed protein expressions during reprogramming because it was reported that gene expressions do not correlate well with protein expressions^20^. We performed a comprehensive analysis of expressed proteins during reprogramming induced by c-MYC and MYCL WT and ΔMB0 mutants. We used SeV-reprogramming HDFs on days 3, 5, and 7 days and EpiP-reprogramming HDFs on day 10 as samples for mass spectrometry (MS) (Fig. 3A) because the percentage of TRA-1-60 ( +) cells was much higher with SeV than with EpiP for observations up to day 7 (Fig. 1C, F). There was more than a two-fold increase in the expression of 520 (SeV) and 128 (EpiP) proteins with MYCL-WT reprogramming compared to c-MYC-WT reprogramming (Fig. 3B, groups (i) and (ii), respectively) and 183 (EpiP) proteins with c-MYC-ΔMB0 reprogramming compared to c-MYC-WT reprogramming (Fig. 3B, group (iii)). Overall, we identified 18 proteins common to the three groups (Fig. 3B, group (iv)). Then, we applied a Gene Ontology (GO) analysis using DAVID and detected enriched terms during reprogramming^21,22^ (Fig. 3C, D, and Table 1), finding cytoskeleton- and cell adhesion-related proteins are involved in the promotion of reprogramming by MYCL-WT. The same analysis was performed to identify proteins whose expression was upregulated by c-MYC-WT compared with MYCL-WT and c-MYC-ΔMB0 (Supplementary Fig. S10A and Table 2). These proteins were associated with the proliferation of non-iPSC-like colonies. We found that c-MYC-WT regulates proteins involved in cell proliferation, such as the cell cycle and DNA replication. To understand the function of the MB0 domain in reprogramming, MS analysis was applied to HDF samples transfected with MYCL-WT, MYCL-ΔMB0, or c-MYC-ΔMB0 (Supplementary Fig. S10B and Table 3). GO analysis indicated that these proteins were associated with cell adhesion and RNA processing.

**Table 1:** MS analysis of identified proteins in cells reprogrammed by MYCL- or c-MYC-ΔMB0.

**(i) Proteins enriched more than two-fold in MYCL-WT compared with c-MYC-WT (SeV)**					
NRP1	PMEL	IQCH	ZNF507	C1QTNF3	GLIPR2
ING1	THOC7	RPAIN	DGCR8	KRAS	PIPOX
CCNL2	ACOT8	KCNMA1	TTC38	MRI1	DLGAP5
CHPF2	P4HA1	HMCN1	STRA13	SMG5	FAM83D
MTFR1L	TSPYL1	CROT	PLPP1	PRKACB	FSD1
REPIN1	MKLN1	CPS1	MDP1	ZWILCH	ST6GAL1
MCL1	SRR	FAM134A	USP34	CEP41	MATN2
AQP1	PPL	SON	ARL14EP	ACSS3	KLHL11
FAIM	MCMBP	COL1A1	MOCS3	SHC1	DPT
VCPIP1	TPM2	CLDN7	C18orf32	SAG	POSTN
AKAP11	AMDHD2	AHCYL2	MASTL	MAP3K2	COPZ2
ARFGEF3	HBA1,HBA2	S100P	CCNYL1	RALGAPB	ACTR1B
PIAS4	PFKFB3	FAM134C	SDSL	PPIC	NR3C1
FYN	SPAST	MAP4K2	COQ3	CENPV	HERC2
CDS2	TADA2B	XPC	MX1	PCSK9	SDPR
CEP131	FEM1A	ACTG1	TNC	ITPR3	GNPTG
SH3BGRL2	QSOX1	LSM4	FBXL18	SH3BP5L	FARP2
ZIC5	PASK	FLYWCH2	TMEM119	FAP	AGTPBP1
ANKIB1	EDEM3	PANX1	CCDC28A	DDX58	FOXK2
ERICH1	KIAA1211	ZMYM4	FN1	ARSA	CSNK1E
MTRR	NCOA3	PATZ1	UBE2S	DDB2	CCDC68
POLG2	C10orf76	ADIRF	CALD1	RALGAPA1	NUDT9
YAE1D1	C14orf142	TSPAN14	PTGIS	FAM208A	PANK1
TCN2	TAGLN	ALG8	THAP11	NFIC	TMEM165
BLOC1S6	FAM21A	NCOR2	COL12A1	TGFBI	CRELD1
MARH5	CNOT8	RANGRF	MED16	CDA	GULP1
WDR54	MET	NOA1	PRKG1	CHMP1A	SHARPIN
RRP8	TBC1D7	CPQ	IFIT1	THBS1	HSDL2
GORAB	TRAF6	AHDC1	DDX60	NDUFB6	ARHGEF6
CERCAM	NPEPL1	GPR107	MAP3K15	MRS2	ELP3
TPM1	COMMD8	MED4	HACL1	IGFBP3	HTRA1
CD99L2	PEX16	GINS4	DSCR3	UBE2G1	EIF4EBP1
DYNC1I2	ACTN1	YPEL5	SMG6	ITGB4	PTGES
TPK1	REEP6	PMF1	PTBP2	IFIT2	PUM1
DYNC2H1	KDELR3	VIPAS39	KIF1B	EMILIN2	CRIP2
HIGD2A	C7orf26	DNM2	MMP2	KANK2	DHX30
RAP1B	DNAJB5	MRPL33	SPANXA2-OT1	PIR	SDCBP
HMGXB4	POLG	FOXK1	PEX1	LGALS8	LAMC1
DNAH6	PDIA4	MTMR14	S100A14	CNTLN	SLC25A32
TMED4	SPARC	GBP1	CNN2	GCC1	CTHRC1
STAU2	SUPV3L1	DNAJC16	KIAA0430	CASP4	NID2
FAM69C	TIMP2	OGFOD3	EED	DCX	PRNP
KCTD15	GSPT2	PCNT	STARD4	OTUD7B	PPP3CC
WDR35	CTSZ	SLC15A4	BASP1	SLC44A1	AKR1C2
COA3	RAB2B	GNA12	OPA3	INPP5A	GAP43
CAAP1	VWA8	PALM	KRT17	MIEF2	IKBKB
C1orf198	BUB1	ZBTB7A	CD248	ACOX1	DNAJA4
CNN1	ANAPC4	LOX	LAMA5	COL2A1	KRT6A
LRRC41	COL6A3	CABIN1	ECM1	MED8	KIF21A
NOL8	SLC30A5	COL16A1	TWISTNB	GREM1	ICAM1
OSBPL11	TBC1D15	HORMAD2	EPHA2	MRPL51	B3GALT6
USP9Y	VKORC1	ETNK1	MACF1	STAG3	SH3KBP1
BCAR1	KHDRBS3	TLE3	IGF2	STARD3NL	CTDSPL2
FIBP	RANBP10	IFT74	SERPINB2	SAMD9	FZD7
LGALS1	CSRP1	FBLN1	SERPINF1	SHCBP1	TUBG1
CAPN5	PTK7	PLAUR	ZNF185	SGF29	RASA3
ACSF3	DNA2	PRSS23	PKP3	GDAP1	CAV2
FBXO2	CCND1	SLC34A3	KYNU	RBM23	ACBD7
MAP2	MIC13	IFI44	PIK3CA	KLC4	ODR4
GATC	TANGO6	MITD1	ATL1	ANAPC13	SP100
MYL9	COL6A2	PPIL2	MPDZ	CCBL1	TGM2
TGS1	CDYL	KRT10	SNX32	OGN	FMNL3
LAMB1	CSRP2	MON2	FAM127A	WDR4	KRT14
BEND3	TRIM21	RPS6KA4	LENG8	SPRR3	FRG1
KLK14	CDC34	ASAP2	TAP2	NEXN	INPP5B
TPM4	PPFIA1	KRT16	LIMK1	HOOK2	PPP2R2D
CPLX1	SUN1	WDR73	WWC3	SMG7	COL1A2
CCDC92	MYCL	DESI2	FYCO1	RAC2	SEMA7A
TIMP3	PKD1L3	COL5A1	PYURF	DPYSL4	SNX24
SERPINB8	FBXO3	RNF31	AKTIP	BAZ2A	UGGT2
HSPB1	CBX2	IFIT3	QPCTL	C1orf50	CAV1
CD58	COL11A2	ISG15	RPL26L1	TYW3	CD44
PARP2	GOLT1B	FOSL1	GRB7	CREG1	HOMER3
HSPB6	ABR	NID1	ECT2	RAP2A	NDRG1
COL5A2	AURKA	GSDMD	ENG	WDR55	WNT5A
MRGBP	EP300	MAU2	CHST14	GHDC	NPHP3
FABP3	ANPEP	ARHGDIB	BST1	NABP2	SIRT5
DTX3L	HAUS7	LTBP2	CLINT1	PXMP4	DPY19L1
ARL5A	RNF113A	CRBN	GGA1	UBE2F	GPNMB
NT5E	CILP	MROH2B	SEPT5	ILF3	TANC1
STX3	NOTCH3	PLCG2	ARFIP1	NCOA5	QSOX2
SLC2A1	S100A6	CDCA5	CALHM2	KDM4B	TIMELESS
F13A1	COMMD9	REN	MECP2	TNXB	ZYG11B
AHNAK2	RDH10	CLIC3	MME	SLC39A14	GGCX
S100A4	ZCCHC6	CD9	CD82	LTBP1	STK11
UAP1L1	MED12	PXN	GOSR2	B3GNT5	ABI3BP
ITGA2	OASL	CTSK	VAMP8		

**(ii) Proteins enriched more than two-fold in MYCL-WT compared with c-MYC-WT (EpiP)**					
**IGFBP3**	**CLU**	**CTHRC1**	**CDH13**	**CAVIN3**	**CCDC80**
**GLIPR2**	**GBP1**	**FLNA**	**LAMB1**	**S100A6**	**COL5A2**
**CNN2**	**ITGA11**	**SH3BGRL3**	**CPQ**	**PRSS23**	**LAMC1**
**NEGR1**	**LAMA5**	**IFIT2**	**LGALS1**	**HTRA1**	**TAGLN**
**TPM2**	**HSPB1**	**EHD2**	**VIM**	**CAVIN1**	**CSRP1**
**TPM1**	**TGFBI**	**S100A11**	**S100A4**	**COL5A1**	**NEXN**
**MOXD1**	SLFN5	NME2P1	FNBP1	PLSCR3	ATPAF2
ZFYVE16	HSBP1	DGKA	PLCD1	ABI3BP	ANXA3
TCF3	PLCB4	SLC34A3	TMEM192	ADAMTSL1	ENDOD1
PTGES	OSBPL9	LRRFIP1	GDPD2	CRYAB	SETDB1
ST6GALNAC1	SMPD1	SETMAR	SORBS3	B4GALT4	CHKB
RNF14	TNXB	AHNAK2	DCTN3	PTBP2	EHBP1L1
TERF2IP	ARMC8	PRC1	UQCC3	PTP4A2	TTYH3
MYCL	BCAT2	ELN	CRMP1	MX2	SGCE
HABP2	TXNIP	RMND5A	TIMP1	PITPNA	DDX49
CHIC1	COL6A3	IMPACT	RAB11B	TMSB4X	SNX7
DDR2	KRT5	PLEKHO2	SMTN	LIPG	RGS3
GREM1	COL5A1	HNRNPDL	LMOD1	MAP4K4	PPP3CB
QTRT2	ARID1A	KRT17	SELENOM	CYP1B1	CD151
MT1X	CTNNA2	KRT6A	MBD5	RAP1B	R3HCC1
VPS37A	STAM2	CTSL	ITGA1	PAIP1	DHRS4
MTPAP	CDK2				
			Bold value: *p* < 0.05		

**(iii) Proteins enriched more than two-fold in c-MYC-ΔMB0 compared with c-MYC-WT (EpiP)**					
ITGA1	PSMF1	FNBP1	BCAT2	DGKA	YIPF3
MBD5	KRT5	S100A6	MOXD1	RMND5A	PPIL3
TAP1	PAIP2	OXR1	MMP2	SCLY	TBC1D10B
CYP1B1	PPDPF	TPM1	PLSCR3	SEMA7A	PPP3CB
RNF115	TMEM192	IGFBP3	MBD2	RBM6	ITPRIP
SPIRE2	OSBPL9	TPM2	TERF2IP	DIP2B	RAB11B
EDC3	SMPD1	DHRS4	LMOD1	OS9	CPQ
COG3	KRT6A	HSBP1	LRRFIP1	CHCHD6	FNTB
CD151	EXOC6B	MROH1	COL6A3	AHNAK2	BCL2L13
RPS6KC1	NCOR2	UQCC3	SELENOM	COG4	DNAJC17
PRC1	CDK2	INTS1	ATPAF2	ENDOD1	STK10
DUSP23	PTGES	CCDC50	TIMP1	TRIM56	YAP1
CHIC1	EHBP1L1	OLFML3	CLU	RAC2	EHD2
SDC1	DNAJC19	CTNNA2	CDH13	ITPA	PITPNA
SNAPIN	PTPN14	LEMD3	SEC24A	CCDC9	MROH9
PAIP1	ANXA3	BAG3	MAP3K20	ELN	NEXN
STAM2	IMPACT	ZFYVE16	TNXB	RMDN2	CRMP1
HABP2	TIMP2	MYO1D	TRAPPC2L	LAMA5	CWC22
DLGAP4	TANC1	DCTN3	CAVIN3	PPIP5K2	LMAN2L
ELP4	SRSF4	KRT17	IFIT2	ERI1	EEF1AKNMT
SMTN	NEGR1	HNRNPDL	ARID1A	PARP12	SCRN3
WDR4	UBAP1	SLFN5	NCOA5	PHRF1	SPARC
CAV1	KRT16	ERCC2	MYO1C	L2HGDH	LPCAT3
ITGA11	NELFA	PXDC1	IL12RB2	EIPR1	DPP7
MCAM	COG1	POLR3C	HSPB1	CAVIN1	GLIPR2
PIKFYVE	C1orf198	SCPEP1	PALM	NKRF	CNN1
RHOB	CUL4B	CCDC80	ARL2BP	GNG12	BMP1
STIM1	VKORC1	CERCAM	CFAP74	ERGIC3	S100A4
TAGLN	MRPS6	KIF4A	P3H4	ALKBH4	PTP4A2
ELMO2	CRYAB	SLC27A1	MAEA	GRB10	THY1
AP1S2	ZNF579	SLC33A1			

**(iv) Commonly enriched proteins in (i) and (ii) and (iii)**					
KRT6A					
PTGES					
S100A6					
TPM1					
IGFBP3					
TPM2					
KRT17					
COL6A3					
TNXB					
IFIT2					
AHNAK2					
LAMA5					
CPQ					
NEXN					
TAGLN					
HSPB1					
GLIPR2					
S100A4					

Four groups are described: (i) proteins whose peptide counts increased more than two-fold in MYCL-WT/HDFs compared with c-MYC-WT/HDFs using SeV on day 3, 5, or 7; (ii) proteins whose peptide counts increased more than two-fold in MYCL-WT compared with c-MYC-WT using EpiP; (iii) proteins whose peptide counts increased more than two-fold in c-MYC-ΔMB0 compared with c-MYC-WT using EpiP; and (iv) commonly identified proteins. Bold fonts in the group (ii) indicate identified proteins with *p* < 0.05 (two-sample paired *t*-test). *n* = 3 for EpiP reprogramming.

**Table 2:** MS analysis of identified proteins in cells reprogrammed with c-MYC.

**(i) Proteins enriched more than two-fold in c-MYC-WT compared with MYCL-WT (SeV)**					
ATXN7L3B	TIMM21	SLC2A3	CA14	CRLF3	SYT6
TMEM161A	MTM1	METTL15	NKAP	CDS2	MRS2
MARS2	ERCC2	TDP1	MFAP4	ANAPC16	CARS2
NOLC1	IGHMBP2	MRPL34	FECH	PARP2	ING1
ADNP2	STEAP3	AK6	PDZD8	EPB41L5	PEX16
ZER1	CKS1B	GGPS1	DBNDD1	MIEF1	FUCA1
ADSSL1	POTEJ	TMEM209	CCNL2	TOP3A	ULK1
MGA	FAM162A	AMMECR1	ISG20L2	CEP78	NOM1
PAPD4	PROCR	IFRD2	LRRC41	UBR3	PHF3
RIN1	SPPL2B	ARAF	DNM2	HPS5	PSEN1
PARD3	ARHGEF16	RHPN2	PRPF18	SEMA4C	RPUSD3
NYNRIN	ARHGEF7	VRTN	PHF10	DMD	RPL26L1
RANBP6	CNOT4	TSPYL5	CDC25C	REEP4	FADD
INPP5F	ZBTB7A	GPN3	RBPMS2	BRAF	ORC6
CACNA2D2	AP1B1	NCAPD3	RRP8	MASTL	POLR2M
CASC3	NCL	C1orf174	LRRC14	SLC27A3	ACSF3
DHRS11	RBM23	WDR55	CAMK4	NDRG3	ALS2
NOVA1	SOX3	CLCN7	EHMT1	C7orf26	NSUN5
NMRAL1	STK25	NFKB2	OSBPL1A	VPS37B	RAD23A
HS2ST1	LYAR	PHKA1	SDC4	MGST2	SNTB1
MEN1	WDR4	DDX28	C1orf198	AKAP9	COQ9
STYX	PHF5A	PCDH1	TMSB4X	AP1G2	MYO1G
UCKL1	APC	TBC1D15	FASTKD1	APCDD1L	MARH5
ULK3	LONP2	SETD1A	ETFDH	ANKS1A	LRP8
PALD1	ANAPC5	CARMIL1	GATM	PANX1	NME3
UBA52	ZNF806	NCOR2	DVL2	CTDP1	PHKB
GINS3	DNPH1	CDCA5	BCKDK	TTF1	TGFBRAP1
HAUS2	HMGCR	SNCA	KLHDC4	TBP	AP1M2
E2F4	AFAP1L1	ZMYM6NB	N4BP2	TRIT1	CCDC134
ATL1	INTS6	CHD8	SPINT2	RASA2	NCK2
MAL2	ATAD3A	SLC25A32	LSAMP	ACOT8	KIFAP3
JARID2	CLSTN1	USP36	PTGIS	PIAS4	TMEM41B
SEC14L1	TUBGCP4	GEMIN8	VWA9	RPP25L	NRBP2
DOLPP1	WARS2	PLEKHA6	MRGBP	ZCCHC6	ZFP36L1
SLC4A7	SCARB1	ARID1B	PMF1	XXYLT1	ANKRD50
MT-CO1	MET	RBM47	LIN28B	EXD2	GORAB
GCSH	PLTP	PRKAB1	CUTC	SDSL	FARS2
LRRC8E	ARHGAP12	FBXW9	PMS2	NAA30	STRA13
FASTKD5	ZCCHC10	TTK	BNC2	COX16	BCS1L
NDE1	STX3	LARP1B	PTCD1	TPD52	SMG1
ACBD7	TRIP12	PTPMT1	ASB3	MTG1	ANKRD12
STK33	HEXIM1	RBM45	ATG9A	ANKIB1	B3GALNT2
C12orf43	SLC25A15	NDUFAF5	BAG4	NOA1	SFRP2
VPRBP	FOXK1	GPM6B	POLE	TRADD	AMFR
RPS6KA1	PLA2G4A	SELO	PROM1	CHTF18	BOD1
SPC24	KAT7	RAB17	IGFBP6	PNPLA4	AGTRAP
UBE2Q1	HIGD2A	RAPH1	SDF2	ARHGAP4	ODR4
MRPS18C	QSOX1	COX17	CHUK	RAPGEF2	GINS1
DFFA	CENPV	PTPN9	FUT11	ERMP1	SOGA1
DHX32	GEMIN6	HDHD2	GLE1	PTPRZ1	CREG1
GATC	PDXP	MID1	WRAP53	POU2F1	CA2
APPL1	TMEM14C	TXNIP	SLC7A3	FABP6	ITGA2
CWC22	MPDZ	PIGG	ACTB	INCENP	CARNMT1
RHBDD2	MRPL38	PUM1	HPDL	NME4	CDKN2A
TRIM27	ARHGEF10L	BDH1	CDC26	CTU1	ATF7
HMGXB4	L3MBTL3	MAP1LC3A	ISLR	URB1	MRPL21
CAMK1	RILPL1	WDR37	IGF2BP1	NAPEPLD	DPH6
FCF1	ANKRD29	ANAPC13	CD3EAP	WDR89	SLC25A17
DNA2	CENPM	CEP170B	GCA	CSTF2T	NKRF
SLC35A2	PMS1	SLC5A6	COQ5	SPR	RBM15B
USP19	LAMA4	DNM1L	INTS2	BCAS3	KIF22
NHEJ1	RPP40	TNC	SPNS1	RPRD1A	DDB2
IFIT5	ARMCX1	FAT1	DAP	BMPR1A	NPHP3
ARID3A	MRPL13	ZBED1	GTF2H1	PATZ1	DPH2
C11orf98	RRP7A	AKAP1	ANAPC1	NUDT16	CD74
DCAKD	ASB6	DNMBP	UTP11L	TUBG1	HAUS7
GAA	PDK1	DOHH	ISYNA1	BRMS1	EXOC6
ARAP3	CHKB	NOL8	DDX52	ORC5	COA7
NAA40	IQSEC1	TRIM24	DNTTIP1	HEATR6	SH3BP4
ZMYM3	MED30	CLASP2	PRPF38B	TMEM256	GTF3C2
MRPL10	NDUFAF7	VCPIP1	HSPB11	CASP7	TOR1B
ITPR2	PRPF39	GCFC2	KIF21A	DPPA4	TIMM8B
GTF2H4	MRPL16	BAZ1A	EXOC6B	MRPL41	POLR3B
USP9Y	SYNE2	MID1IP1	MOCS2	METTL5	PRUNE
UBE2V2	CDH1	GJA1	CHMP6	RCC1	RAPGEF6
COMMD9	HMBS	SQLE	IGFBP2	DIAPH2	EPB41
HIST1H1E	MAP3K4	CDK18	ARL15	UQCC1	HTATIP2
PTDSS2	TATDN2	MTA1	SNX18	EIF1B	MAEA
SCAF1	UBE2J2	NUP37	BAG1	TSSC4	RCOR2
FAM213A	MFF	TTLL12	UBXN6	EFEMP2	ZZEF1
NFYB	PDS5B	FXN	AGPAT5	ARFGEF2	TCOF1
SCAF11	RAPGEF1	PARG	PRIM1	USF1	L1TD1
SEPHS1	BRD8	POP7	EXT2	TSEN34	CAMKV
SLC29A1	MBD3	PPIF	MTX3	COG1	FASTKD2
ARL14EP	MRPL40	CD320	MBD1	VARS2	BRD3
MRPS18B	ACY1	PPIE	RIF1	VPS8	POGZ
RSL1D1	PAK1	STRBP	TERF2	TOP2A	SLC39A10
QRICH1	CISD1	POLB	DHX37	TRMT5	TIMMDC1
TRABD	SON	SET	LYST	RNASEH2B	MVK
ZC3HC1	NDUFC1	HSPA4L	EXOSC1	TCEANC2	NASP
CYP2S1	NSMCE3	HPRT1	HUS1	PRRC2A	RDH13
KANK1	PHF14	RBM7	SIRT5	QPCTL	CROCC
LIMK2	CWF19L1	KIF1B	VPS39	CHD7	YPEL5
SLC7A5	VPS25	LRWD1	FPGS	NT5C3A	NCKIPSD
PSIP1	CELF2	MDC1	ANP32A	ACADSB	GPC4
TUBB4A	BOP1	NT5C	CTSC	ANKLE2	ORC4
UTP18	CHST14	NUS1	PLK1	GPKOW	SIKE1
ADAM15	NUDCD2	SSU72	NFYC	LIG1	MSH2
HNRNPR	MSI2	STK26	EBP	ITPK1	STAU2
URI1	SLC7A8	MANBAL	RNGTT	PRIM2	SEC24B
MRPS34	RMND5A	F11R	SCAF8	RPAP1	SPATA5L1
RCC2	POLRMT	SERF2	TMEM115	PARP1	C2CD5
BEND3	BRAT1	TERF2IP	MT-ND2	FAM136A	NOP2
ARL5B	GFM2	WDR3	AARSD1	PPID	HRSP12
CHD2	MRPL45	YTHDF1	THEMIS	INTS4	PLEKHA1
CBX3	SIRT7	CENPF	CAMSAP2	MCC	PTPN2
NANOG	SLBP	MCAT	MRPL24	MZT1	ANAPC4
HIST1H1A	KPNA2	MED14	NDUFB9	NEFL	DUS3L
HSP90AA4P	CMSS1	ZNF706	PTRHD1	PBRM1	ABI3BP
MED10	PFAS	BRIX1	QRSL1	THNSL1	ADRM1
CECR2	NCOA5	ABRACL	WIPF2	USP28	GEMIN4
CHD1	SBNO1	LSM12	COG2	TARS2	KIF11
CHD1L	MLH1	MNAT1	DDX47	SRPK1	NACAP1
XPC	NELFCD	MPP6	HAUS6	TANC1	PPAN
MRPL33	PUM2	MRPL15	FAM65A	ERI3	TOM1L2
TIMM13	SPRYD4	MICU1	HMGN5	XPO4	WDR43
DDX20	YTHDC2	SLC25A22	CACUL1	PCF11	PRC1
DNAAF2	ACAT1	RWDD4	DHPS	PFKM	NUDT16L1
MTMR2	GPN1	SMARCA4	EI24	SCFD2	PPP3CB
RNASEH2A	GTF2E2	SMARCD1	ADCK3	TRMT1	RABEP1
CD97	GLT8D1	UBR5	HLTF	TXNRD2	TDP2
CCNY	NCAPD2	USP48	LRCH2	UBQLN1	TRIP10
MCM3	HSPA14	ZBTB8OS	MAP2K7	APOBEC3C	TTC27
DYSF	MINA	BAK1	HERC2	POLG2	TRAP1
EXOSC7	RCL1	C17orf62	PUS7	CASP6	ISOC1
THYN1	MYL6	CBR4	RFC3	SRSF10	LCLAT1
HIBADH	UHRF1	DHX57	MCM6	UBL4A	MRPS17
TFRC	GALK2	MKI67	CEBPZ	CHRAC1	TTC4
METTL3	GSTZ1	UBE2O	LRSAM1	NSA2	AGPAT4
MRPS5	MAP3K7	ZNF330	MLLT11	HK2	BLMH
PCCA	LYPLAL1	CPSF2	PEX3	INPP4A	SORD
SLC35F2	MRPL23	GNL3	WBP11	SAP30	INPPL1
TBC1D9B	MRPS31	GMPS	IMPDH2	SSRP1	LBR
TRIM28	NELFB	LDAH	DDX21	TWSG1	RBMX
NDUFAF1	PHF6	TFB2M	EBNA1BP2	ZMYM2	MLYCD
UROD	PKP3	NUBP2	FANCI	ARIH2	ZNF22
USP11	ARFRP1	PKN1	FAR1	MYBBP1A	PDS5A
CHMP7	COASY	SAP18	GTF3C4	SMARCAD1	DNAAF5
CTAGE5	GART	UBE2I	MRPL27	ENY2	PDK3
RUVBL1	PEAK1	WDR6	MYEF2	PWP2	RPA2
EXOSC4	FRA10AC1	ADAD2	NARS2	FAM64A	XRCC4
GTF2E1	DDX51	BMS1	OGFR	MRPL3	CHAC2
PES1	IGF2BP3	LDHB	ORC3	MSH6	DPYSL5
HEATR1	KATNA1	PTMA	SLC52A2	NLE1	MCM7
POLD1	MCM2	CXorf56	SMAD5	GPATCH4	STOML2
FLVCR1	MYH14	DAXX	ZCCHC8	TAMM41	TOMM34
LGALS3BP	PARN	KIF1BP	C5orf22	TP53RK	NACC1
MRPS30	TKT	ELAC2	PRKCI	PDCD11	DNAJB4
MTMR12	SUPV3L1	DARS2	RAVER1	SIGMAR1	DHODH
SLC3A2	BTAF1	HSDL1	EIF3C	TBPL1	NOC2L
MRPS28	CADM1	MRPL37	SRI	BZW2	NDUFC2
PCCB	DNTTIP2	PCBD2	TRIM2	CPSF1	RABGGTB
RFC5	ECT2	AGTPBP1	TRIM22	ECM29	PANK4
C11orf73	MCAM	THUMPD1	WDR18	RNPS1	SCO1
TIMM17B	INTS8	TUSC3	DCAF16	GTF2A1	SIRT1
UTP6	MRPL11	XRN1	DCTPP1	HSPBP1	TUFM
WDR92	PIR	ACAA2	DNAJC2	NPM3	ADNP
CERS6	NUDT12	APTX	DSG2	NTHL1	BOLA3
DDX24	NUP35	ATL2	NOL11	DDX31	ATPAF1
GCDH	RCHY1	C12orf10	HIP1R	PRPS2	MCM5
WDR75	RHOT1	KIAA1211	DDX54	RSPRY1	CDC123
NOP16	TACO1	GLMN	HOOK1	WNK1	NUP155
POLR1A	TBL3	GNAI1	KIAA2013	C7orf50	GUSB
GUF1	TIMM17A	GRWD1	LBH	CCNK	ILKAP
SMYD5	AGL	POR	LIG3	CMTR1	LRBA
WDR5	TOMM5	ARL8A	PCK2	PDCD4	NAT10
ACO1	TSC2	GNL3L	MTFP1	ABT1	KEAP1
ANKRD28	UBTF	STK3	SCAP	PPAT	PSAT1
APOA1BP	WDR54	SYT1	SCRIB	PTBP3	RNASEH2C
APOO	ASF1A	GSPT2	SDHB	SUPT16H	DBR1
PSME3	PPWD1	UBQLN4	CHAF1A	TRMT10C	SACS
BYSL	CDH13	USP24	ARID1A	GJB2	TEX10
UBA2	CLUH	ARL2	AS3MT	ABCC1	MRPL57
DDX56	EIF2D	ATP1B3	DCAF8	AGK	AIF1L
RBM42	FEN1	CCDC12	ELOVL6	EFNB1	NCAPG2
SARS2	OSBPL2	CDCA8	GNA13	ATF7IP	CCDC50
ADI1	HAUS8	C1orf131	GOLM1	DEK	DTD1
MCL1	INTS1	AHCY	GYS1	PAICS	DTWD1
CCDC59	MAK16	RUVBL2	ISY1	ECT2L	GTPBP10
ZNF593	RBM26	FDXR	MRI1	HS1BP3	LSM6
DNAJC8	SALL4	AATF	LETM1	LAS1L	MRPS11
SMARCA5	TMPO	LTA4H	MYCBP	MRPL19	NEPRO
PCBP2	WBSCR22	CCNH	DLGAP5	NDUFS7	NOP58
HDAC2	WDR48	GLTSCR2	PELP1	HARS2	NT5DC1
HIST1H1B	TOX4	TPI1	LUC7L	ATP11C	NXF1
HNRNPC	VRK1	KDM2A	COMMD8	CACNA2D1	RAD21
HNRNPU	WIPI1	USP39	SSB	CDCA7L	SNF8
LARS	ASH2L	VPS36	ZNF346	CRNKL1	TELO2
LRRC57	AASDHPPT	CNPY3	GLUL	CWC27	TNPO3
CHORDC1	CKAP2	CXADR	ILF2	PEG10	TTI2
MANBA	DCAF13	PLS1	LVRN	C1QBP	UBE2A
MEMO1	EIF4A3	EXTL2	MED24	GLYR1	UBXN1
MRPS18A	EIF5B	NIFK	POLR2H	HMOX2	ABCB6
NDUFB4	FNBP1L	NOL10	KIF5C	EXOSC9	ATP2B1
TOMM40	RBM28	GSTP1	TATDN1	FTSJ3	DAGLB
PLCG1	IFI16	HSPA4	TMEM192	MPI	SMPD4
POLE4	KDM1A	NUP188	TSFM	DLAT	FAM210A
POLR2D	NOL6	PRPF40A	UBAP2	MSTO1	GTF2I
SHPK	NUP133	RBM12B	DAB2IP	SAFB	LARP1
SPCS1	PDHA1	STX18	PPFIA1	NUP50	METTL13
SAAL1	QTRTD1	SUPT6H	ADSL	QSER1	POLR3C
TRMT1L	ASUN	BMP1	AFG3L2	GEMIN5	NEU1
PEX6	HDAC3	NDC80	MRRF	HMGN1	TIMM44
REEP6	OTX2	LPCAT1	MTPAP	SNU13	UMPS
TRIAP1	TUT1	GRSF1	NAA20	ACSS3	NCK1
ERBB2	CCDC28B	XRN2	NDUFAF4	MIF	PPP2R5A
SGSH	CRADD	MT-ND4	GATAD2A	MRPL9	RAD50
OSGEPL1	ADGRL2	MTUS2	NRBF2	FAM192A	DNPEP
THAP11	HEATR5B	RABGEF1	POP1	HEATR3	SRRM2
CACTIN	PRKD1	MRPL50	PRMT1	NIPBL	STK4
SLC25A35	ECE2	RPL13A	SDCBP	NTMT1	TBRG4
ZFAND6	DSEL	SDAD1	RPL21	PDCD5	PPIG
RALGAPB	MYO5C	TRMT6	RBM39	GULP1	DCP1A
IGSF1	POLG	UBE3A	SLC30A1	RFC4	TUBAL3
MGRN1	TPK1	KCTD10	SMAP	ACIN1	UBXN7
TMEM41A	DNAL1	F8A1,F8A2,F8A3	TKFC	BSG	ARHGEF40
FBP1	FBXL6	DFFB	TRIM33	EHBP1	ATP5S
CDC20	PLEKHA7	WDR73	CD70	EMC3	SLC9A3R1
COBLL1	TRIM9	SP1	YARS2	RBM19	CKMT1A,CKMT1B
HSP90AB3P	MRPL35	CCNA2	HDDC2	KDM3B	PPA1
SMARCAL1	MALSU1	AURKB	PPM1G	EARS2	HSP90AA1
SH3GL3	MPHOSPH6	NACC2	HMGB2	GTPBP4	ABCB10
CYP2U1	BSDC1	TLK1	LEO1	MFAP1	GIT1
FLCN	TYRO3	SH3PXD2A	EMC4	CPS1	INTS9
IRS2	SIRT3	FUT8	CDK2	TNFAIP6	SDHAF4
KITLG	ALDH3A1	GPR180	CFAP36	ANK3	CBX2
RBBP9	EML2	POLR3GL	FN3K	VWA5A	RAVER2
ATXN7L3B	TIMM21	SLC2A3	CA14	CRLF3	SYT6
TMEM161A	MTM1	METTL15	NKAP	CDS2	MRS2
MARS2	ERCC2	TDP1	MFAP4	ANAPC16	CARS2
NOLC1	IGHMBP2	MRPL34	FECH	PARP2	ING1
ADNP2	STEAP3	AK6	PDZD8	EPB41L5	PEX16
ZER1	CKS1B	GGPS1	DBNDD1	MIEF1	FUCA1
ADSSL1	POTEJ	TMEM209	CCNL2	TOP3A	ULK1
MGA	FAM162A	AMMECR1	ISG20L2	CEP78	NOM1
PAPD4	PROCR	IFRD2	LRRC41	UBR3	PHF3
RIN1	SPPL2B	ARAF	DNM2	HPS5	PSEN1
PARD3	ARHGEF16	RHPN2	PRPF18	SEMA4C	RPUSD3
NYNRIN	ARHGEF7	VRTN	PHF10	DMD	RPL26L1
RANBP6	CNOT4	TSPYL5	CDC25C	REEP4	FADD
INPP5F	ZBTB7A	GPN3	RBPMS2	BRAF	ORC6
CACNA2D2	AP1B1	NCAPD3	RRP8	MASTL	POLR2M
CASC3	NCL	C1orf174	LRRC14	SLC27A3	ACSF3
DHRS11	RBM23	WDR55	CAMK4	NDRG3	ALS2
NOVA1	SOX3	CLCN7	EHMT1	C7orf26	NSUN5
NMRAL1	STK25	NFKB2	OSBPL1A	VPS37B	RAD23A
HS2ST1	LYAR	PHKA1	SDC4	MGST2	SNTB1
MEN1	WDR4	DDX28	C1orf198	AKAP9	COQ9
STYX	PHF5A	PCDH1	TMSB4X	AP1G2	MYO1G
UCKL1	APC	TBC1D15	FASTKD1	APCDD1L	MARH5
ULK3	LONP2	SETD1A	ETFDH	ANKS1A	LRP8
PALD1	ANAPC5	CARMIL1	GATM	PANX1	NME3
UBA52	ZNF806	NCOR2	DVL2	CTDP1	PHKB
GINS3	DNPH1	CDCA5	BCKDK	TTF1	TGFBRAP1
HAUS2	HMGCR	SNCA	KLHDC4	TBP	AP1M2
E2F4	AFAP1L1	ZMYM6NB	N4BP2	TRIT1	CCDC134
ATL1	INTS6	CHD8	SPINT2	RASA2	NCK2
MAL2	ATAD3A	SLC25A32	LSAMP	ACOT8	KIFAP3
JARID2	CLSTN1	USP36	PTGIS	PIAS4	TMEM41B
SEC14L1	TUBGCP4	GEMIN8	VWA9	RPP25L	NRBP2
DOLPP1	WARS2	PLEKHA6	MRGBP	ZCCHC6	ZFP36L1
SLC4A7	SCARB1	ARID1B	PMF1	XXYLT1	ANKRD50
MT-CO1	MET	RBM47	LIN28B	EXD2	GORAB
GCSH	PLTP	PRKAB1	CUTC	SDSL	FARS2
LRRC8E	ARHGAP12	FBXW9	PMS2	NAA30	STRA13
FASTKD5	ZCCHC10	TTK	BNC2	COX16	BCS1L
NDE1	STX3	LARP1B	PTCD1	TPD52	SMG1
ACBD7	TRIP12	PTPMT1	ASB3	MTG1	ANKRD12
STK33	HEXIM1	RBM45	ATG9A	ANKIB1	B3GALNT2
C12orf43	SLC25A15	NDUFAF5	BAG4	NOA1	SFRP2
VPRBP	FOXK1	GPM6B	POLE	TRADD	AMFR
RPS6KA1	PLA2G4A	SELO	PROM1	CHTF18	BOD1
SPC24	KAT7	RAB17	IGFBP6	PNPLA4	AGTRAP
UBE2Q1	HIGD2A	RAPH1	SDF2	ARHGAP4	ODR4
MRPS18C	QSOX1	COX17	CHUK	RAPGEF2	GINS1
DFFA	CENPV	PTPN9	FUT11	ERMP1	SOGA1
DHX32	GEMIN6	HDHD2	GLE1	PTPRZ1	CREG1
GATC	PDXP	MID1	WRAP53	POU2F1	CA2
APPL1	TMEM14C	TXNIP	SLC7A3	FABP6	ITGA2
CWC22	MPDZ	PIGG	ACTB	INCENP	CARNMT1
RHBDD2	MRPL38	PUM1	HPDL	NME4	CDKN2A
TRIM27	ARHGEF10L	BDH1	CDC26	CTU1	ATF7
HMGXB4	L3MBTL3	MAP1LC3A	ISLR	URB1	MRPL21
CAMK1	RILPL1	WDR37	IGF2BP1	NAPEPLD	DPH6
FCF1	ANKRD29	ANAPC13	CD3EAP	WDR89	SLC25A17
DNA2	CENPM	CEP170B	GCA	CSTF2T	NKRF
SLC35A2	PMS1	SLC5A6	COQ5	SPR	RBM15B
USP19	LAMA4	DNM1L	INTS2	BCAS3	KIF22
NHEJ1	RPP40	TNC	SPNS1	RPRD1A	DDB2
IFIT5	ARMCX1	FAT1	DAP	BMPR1A	NPHP3
ARID3A	MRPL13	ZBED1	GTF2H1	PATZ1	DPH2
C11orf98	RRP7A	AKAP1	ANAPC1	NUDT16	CD74
DCAKD	ASB6	DNMBP	UTP11L	TUBG1	HAUS7
GAA	PDK1	DOHH	ISYNA1	BRMS1	EXOC6
ARAP3	CHKB	NOL8	DDX52	ORC5	COA7
NAA40	IQSEC1	TRIM24	DNTTIP1	HEATR6	SH3BP4
ZMYM3	MED30	CLASP2	PRPF38B	TMEM256	GTF3C2
MRPL10	NDUFAF7	VCPIP1	HSPB11	CASP7	TOR1B
ITPR2	PRPF39	GCFC2	KIF21A	DPPA4	TIMM8B
GTF2H4	MRPL16	BAZ1A	EXOC6B	MRPL41	POLR3B
USP9Y	SYNE2	MID1IP1	MOCS2	METTL5	PRUNE
UBE2V2	CDH1	GJA1	CHMP6	RCC1	RAPGEF6
COMMD9	HMBS	SQLE	IGFBP2	DIAPH2	EPB41
HIST1H1E	MAP3K4	CDK18	ARL15	UQCC1	HTATIP2
PTDSS2	TATDN2	MTA1	SNX18	EIF1B	MAEA
SCAF1	UBE2J2	NUP37	BAG1	TSSC4	RCOR2
FAM213A	MFF	TTLL12	UBXN6	EFEMP2	ZZEF1
NFYB	PDS5B	FXN	AGPAT5	ARFGEF2	TCOF1
SCAF11	RAPGEF1	PARG	PRIM1	USF1	L1TD1
SEPHS1	BRD8	POP7	EXT2	TSEN34	CAMKV
SLC29A1	MBD3	PPIF	MTX3	COG1	FASTKD2
ARL14EP	MRPL40	CD320	MBD1	VARS2	BRD3
MRPS18B	ACY1	PPIE	RIF1	VPS8	POGZ
RSL1D1	PAK1	STRBP	TERF2	TOP2A	SLC39A10
QRICH1	CISD1	POLB	DHX37	TRMT5	TIMMDC1
TRABD	SON	SET	LYST	RNASEH2B	MVK
ZC3HC1	NDUFC1	HSPA4L	EXOSC1	TCEANC2	NASP
CYP2S1	NSMCE3	HPRT1	HUS1	PRRC2A	RDH13
KANK1	PHF14	RBM7	SIRT5	QPCTL	CROCC
LIMK2	CWF19L1	KIF1B	VPS39	CHD7	YPEL5
SLC7A5	VPS25	LRWD1	FPGS	NT5C3A	NCKIPSD
PSIP1	CELF2	MDC1	ANP32A	ACADSB	GPC4
TUBB4A	BOP1	NT5C	CTSC	ANKLE2	ORC4
UTP18	CHST14	NUS1	PLK1	GPKOW	SIKE1
ADAM15	NUDCD2	SSU72	NFYC	LIG1	MSH2
HNRNPR	MSI2	STK26	EBP	ITPK1	STAU2
URI1	SLC7A8	MANBAL	RNGTT	PRIM2	SEC24B
MRPS34	RMND5A	F11R	SCAF8	RPAP1	SPATA5L1
RCC2	POLRMT	SERF2	TMEM115	PARP1	C2CD5
BEND3	BRAT1	TERF2IP	MT-ND2	FAM136A	NOP2
ARL5B	GFM2	WDR3	AARSD1	PPID	HRSP12
CHD2	MRPL45	YTHDF1	THEMIS	INTS4	PLEKHA1
CBX3	SIRT7	CENPF	CAMSAP2	MCC	PTPN2
NANOG	SLBP	MCAT	MRPL24	MZT1	ANAPC4
HIST1H1A	KPNA2	MED14	NDUFB9	NEFL	DUS3L
HSP90AA4P	CMSS1	ZNF706	PTRHD1	PBRM1	ABI3BP
MED10	PFAS	BRIX1	QRSL1	THNSL1	ADRM1
CECR2	NCOA5	ABRACL	WIPF2	USP28	GEMIN4
CHD1	SBNO1	LSM12	COG2	TARS2	KIF11
CHD1L	MLH1	MNAT1	DDX47	SRPK1	NACAP1
XPC	NELFCD	MPP6	HAUS6	TANC1	PPAN
MRPL33	PUM2	MRPL15	FAM65A	ERI3	TOM1L2
TIMM13	SPRYD4	MICU1	HMGN5	XPO4	WDR43
DDX20	YTHDC2	SLC25A22	CACUL1	PCF11	PRC1
DNAAF2	ACAT1	RWDD4	DHPS	PFKM	NUDT16L1
MTMR2	GPN1	SMARCA4	EI24	SCFD2	PPP3CB
RNASEH2A	GTF2E2	SMARCD1	ADCK3	TRMT1	RABEP1
CD97	GLT8D1	UBR5	HLTF	TXNRD2	TDP2
CCNY	NCAPD2	USP48	LRCH2	UBQLN1	TRIP10
MCM3	HSPA14	ZBTB8OS	MAP2K7	APOBEC3C	TTC27
DYSF	MINA	BAK1	HERC2	POLG2	TRAP1
EXOSC7	RCL1	C17orf62	PUS7	CASP6	ISOC1
THYN1	MYL6	CBR4	RFC3	SRSF10	LCLAT1
HIBADH	UHRF1	DHX57	MCM6	UBL4A	MRPS17
TFRC	GALK2	MKI67	CEBPZ	CHRAC1	TTC4
METTL3	GSTZ1	UBE2O	LRSAM1	NSA2	AGPAT4
MRPS5	MAP3K7	ZNF330	MLLT11	HK2	BLMH
PCCA	LYPLAL1	CPSF2	PEX3	INPP4A	SORD
SLC35F2	MRPL23	GNL3	WBP11	SAP30	INPPL1
TBC1D9B	MRPS31	GMPS	IMPDH2	SSRP1	LBR
TRIM28	NELFB	LDAH	DDX21	TWSG1	RBMX
NDUFAF1	PHF6	TFB2M	EBNA1BP2	ZMYM2	MLYCD
UROD	PKP3	NUBP2	FANCI	ARIH2	ZNF22
USP11	ARFRP1	PKN1	FAR1	MYBBP1A	PDS5A
CHMP7	COASY	SAP18	GTF3C4	SMARCAD1	DNAAF5
CTAGE5	GART	UBE2I	MRPL27	ENY2	PDK3
RUVBL1	PEAK1	WDR6	MYEF2	PWP2	RPA2
EXOSC4	FRA10AC1	ADAD2	NARS2	FAM64A	XRCC4
GTF2E1	DDX51	BMS1	OGFR	MRPL3	CHAC2
PES1	IGF2BP3	LDHB	ORC3	MSH6	DPYSL5
HEATR1	KATNA1	PTMA	SLC52A2	NLE1	MCM7
POLD1	MCM2	CXorf56	SMAD5	GPATCH4	STOML2
FLVCR1	MYH14	DAXX	ZCCHC8	TAMM41	TOMM34
LGALS3BP	PARN	KIF1BP	C5orf22	TP53RK	NACC1
MRPS30	TKT	ELAC2	PRKCI	PDCD11	DNAJB4
MTMR12	SUPV3L1	DARS2	RAVER1	SIGMAR1	DHODH
SLC3A2	BTAF1	HSDL1	EIF3C	TBPL1	NOC2L
MRPS28	CADM1	MRPL37	SRI	BZW2	NDUFC2
PCCB	DNTTIP2	PCBD2	TRIM2	CPSF1	RABGGTB
RFC5	ECT2	AGTPBP1	TRIM22	ECM29	PANK4
C11orf73	MCAM	THUMPD1	WDR18	RNPS1	SCO1
TIMM17B	INTS8	TUSC3	DCAF16	GTF2A1	SIRT1
UTP6	MRPL11	XRN1	DCTPP1	HSPBP1	TUFM
WDR92	PIR	ACAA2	DNAJC2	NPM3	ADNP
CERS6	NUDT12	APTX	DSG2	NTHL1	BOLA3
DDX24	NUP35	ATL2	NOL11	DDX31	ATPAF1
GCDH	RCHY1	C12orf10	HIP1R	PRPS2	MCM5
WDR75	RHOT1	KIAA1211	DDX54	RSPRY1	CDC123
NOP16	TACO1	GLMN	HOOK1	WNK1	NUP155
POLR1A	TBL3	GNAI1	KIAA2013	C7orf50	GUSB
GUF1	TIMM17A	GRWD1	LBH	CCNK	ILKAP
SMYD5	AGL	POR	LIG3	CMTR1	LRBA
WDR5	TOMM5	ARL8A	PCK2	PDCD4	NAT10
ACO1	TSC2	GNL3L	MTFP1	ABT1	KEAP1
ANKRD28	UBTF	STK3	SCAP	PPAT	PSAT1
APOA1BP	WDR54	SYT1	SCRIB	PTBP3	RNASEH2C
APOO	ASF1A	GSPT2	SDHB	SUPT16H	DBR1
PSME3	PPWD1	UBQLN4	CHAF1A	TRMT10C	SACS
BYSL	CDH13	USP24	ARID1A	GJB2	TEX10
UBA2	CLUH	ARL2	AS3MT	ABCC1	MRPL57
DDX56	EIF2D	ATP1B3	DCAF8	AGK	AIF1L
RBM42	FEN1	CCDC12	ELOVL6	EFNB1	NCAPG2
SARS2	OSBPL2	CDCA8	GNA13	ATF7IP	CCDC50
ADI1	HAUS8	C1orf131	GOLM1	DEK	DTD1
MCL1	INTS1	AHCY	GYS1	PAICS	DTWD1
CCDC59	MAK16	RUVBL2	ISY1	ECT2L	GTPBP10
ZNF593	RBM26	FDXR	MRI1	HS1BP3	LSM6
DNAJC8	SALL4	AATF	LETM1	LAS1L	MRPS11
SMARCA5	TMPO	LTA4H	MYCBP	MRPL19	NEPRO
PCBP2	WBSCR22	CCNH	DLGAP5	NDUFS7	NOP58
HDAC2	WDR48	GLTSCR2	PELP1	HARS2	NT5DC1
HIST1H1B	TOX4	TPI1	LUC7L	ATP11C	NXF1
HNRNPC	VRK1	KDM2A	COMMD8	CACNA2D1	RAD21
HNRNPU	WIPI1	USP39	SSB	CDCA7L	SNF8
LARS	ASH2L	VPS36	ZNF346	CRNKL1	TELO2
LRRC57	AASDHPPT	CNPY3	GLUL	CWC27	TNPO3
CHORDC1	CKAP2	CXADR	ILF2	PEG10	TTI2
MANBA	DCAF13	PLS1	LVRN	C1QBP	UBE2A
MEMO1	EIF4A3	EXTL2	MED24	GLYR1	UBXN1
MRPS18A	EIF5B	NIFK	POLR2H	HMOX2	ABCB6
NDUFB4	FNBP1L	NOL10	KIF5C	EXOSC9	ATP2B1
TOMM40	RBM28	GSTP1	TATDN1	FTSJ3	DAGLB
PLCG1	IFI16	HSPA4	TMEM192	MPI	SMPD4
POLE4	KDM1A	NUP188	TSFM	DLAT	FAM210A
POLR2D	NOL6	PRPF40A	UBAP2	MSTO1	GTF2I
SHPK	NUP133	RBM12B	DAB2IP	SAFB	LARP1
SPCS1	PDHA1	STX18	PPFIA1	NUP50	METTL13
SAAL1	QTRTD1	SUPT6H	ADSL	QSER1	POLR3C
TRMT1L	ASUN	BMP1	AFG3L2	GEMIN5	NEU1
PEX6	HDAC3	NDC80	MRRF	HMGN1	TIMM44
REEP6	OTX2	LPCAT1	MTPAP	SNU13	UMPS
TRIAP1	TUT1	GRSF1	NAA20	ACSS3	NCK1
ERBB2	CCDC28B	XRN2	NDUFAF4	MIF	PPP2R5A
SGSH	CRADD	MT-ND4	GATAD2A	MRPL9	RAD50
OSGEPL1	ADGRL2	MTUS2	NRBF2	FAM192A	DNPEP
THAP11	HEATR5B	RABGEF1	POP1	HEATR3	SRRM2
CACTIN	PRKD1	MRPL50	PRMT1	NIPBL	STK4
SLC25A35	ECE2	RPL13A	SDCBP	NTMT1	TBRG4
ZFAND6	DSEL	SDAD1	RPL21	PDCD5	PPIG
RALGAPB	MYO5C	TRMT6	RBM39	GULP1	DCP1A
IGSF1	POLG	UBE3A	SLC30A1	RFC4	TUBAL3
MGRN1	TPK1	KCTD10	SMAP	ACIN1	UBXN7
TMEM41A	DNAL1	F8A1,F8A2,F8A3	TKFC	BSG	ARHGEF40
FBP1	FBXL6	DFFB	TRIM33	EHBP1	ATP5S
CDC20	PLEKHA7	WDR73	CD70	EMC3	SLC9A3R1
COBLL1	TRIM9	SP1	YARS2	RBM19	CKMT1A,CKMT1B
HSP90AB3P	MRPL35	CCNA2	HDDC2	KDM3B	PPA1
SMARCAL1	MALSU1	AURKB	PPM1G	EARS2	HSP90AA1
SH3GL3	MPHOSPH6	NACC2	HMGB2	GTPBP4	ABCB10
CYP2U1	BSDC1	TLK1	LEO1	MFAP1	GIT1
FLCN	TYRO3	SH3PXD2A	EMC4	CPS1	INTS9
IRS2	SIRT3	FUT8	CDK2	TNFAIP6	SDHAF4
KITLG	ALDH3A1	GPR180	CFAP36	ANK3	CBX2
RBBP9	EML2	POLR3GL	FN3K	VWA5A	RAVER2

**(ii) Proteins enriched more than two-fold in c-MYC-WT compared with MYCL-WT (EpiP)**					
FIGNL2	MCEE	NDUFB7	BCCIP	CCDC86	HSPA4L
URB1	INTS14	MRPS10	PDHX	SNCA	RPL26L1
NBN	DNAJC2	NDUFAF2	WDR36	UBE2G1	INTS3
NDUFB1	EIF3C	WARS2	SUPV3L1	TOP2A	ANKZF1
PCCA	AKAP9	NOP16	EHMT2	MRPL3	KAT7
POLR3D	MAPKAP1	PAIP1	GK	INTS13	MT-ATP6
NF1	NABP2	OSBPL11	IMPDH2	DIEXF	WDR73
BRD2	MCM7	SFXN4	HSPD1	REXO4	GEMIN4
NCAPD3	NDFIP1	BOP1	PM20D2	TRMT5	STEAP3
SSR2	MTA3	TOMM40	PDS5A	SLC25A17	RIDA
GTF2A2	DCUN1D5	ORC5	CDKN2AIPNL	SET	TFAM
CORO7	THTPA	POLR1B	HSPE1	APOO	NDUFS7
UBE2G2	WRAP53	HARS2	POLRMT	IRF9	RBM19
PPP4R3B	DPCD	FBXO22	CLUH	TSR1	ZNF740
ATP7A	HIGD2A	TIMM17A	COA7	ALKBH5	TTI1
ATR	ITPA	PABPN1	TYMS	RARS2	PHC2
MBTPS1	FAM234A	LPCAT3	BMS1	INTS11	UBE2S
VAMP3	TTC12	TNPO2	NOC3L	TBL3	POLR1A
PFKFB3	CARS2	FBL	LSAMP	ABHD11	ORC2
SH3PXD2A	INCENP	SMN1,SMN2	GNL3	MRPS15	MRPL17
ZNF318	ITM2C	NIP7	SYF2	UCK2	MARS2
CWF19L1	INTS9	CDK1	STK26	IRF2BP2	PTCD1
RBX1	POLR3B	SLC4A7	DNPH1	DIMT1	DUS3L
ULK3	IFT57	NDUFAF4	NGDN	PCBP2	CEPT1
TTC33	ARMCX1	HDDC3	PPIF	MDC1	PES1
CWC27	TRAP1	BRIX1	TOMM6	NCAPG	PWP2
NOL11	DDX41	GCA	DDX20	HEATR1	NT5DC2
KDM3B	TASOR	NCL	MGST1	GTF2H4	SSNA1
ZC3HC1	PALD1	EEF1E1	TRMT10C	NUDT3	COIL
VRK2	SYNPO2	PSMF1	PTBP3	TGS1	NOL10
DDX60	HIGD1A	RAB11FIP5	C1QBP	BCS1L	POLD3
PHACTR4	SIK3	TRIM65	DNMT1	LYAR	YY1
ASPM	CABIN1	WDR43	POLD2	WDR3	DCAF1
GPD1L	MAP3K4	MYC	FAM162A	DDX18	WDHD1
GTF3C2	MYBBP1A	URB2	HMGN5	BAZ1A	MDP1
STAG1	DDX21	VRK1	GUCY1B2	SLC1A3	MTMR6
COX6A1	TMEM33	UBE2D3	RFC5	RRP1	NEDD4
SAAL1	NPM3	TEX10	CDK2AP1	POLR2F	RSL1D1
EDC3	PLA2G4A	HMGA1	DDX24	TRRAP	SELENOO
DTD2	MED23	PBX1	UTP4	H1-4	FANCI
SLC35E1	PEG10	GRPEL1	DPH2	DNAJA3	HAUS1
CCDC115	NOLC1	GNL2	POLD1	MRPS2	ZNF565
PVR	AKAP1	PRRX1	NDUFAF3	PARP12	STRIP1
ZCCHC3	NELFCD	PODXL2	PRR35	KDM1B	BMI1
TRAPPC8	SPNS1	BLOC1S4	UMODL1	KIF21B	C8orf33
NFIX	AFP	TTN	RAVER2	NFATC2IP	CHD1
UBE2D1	DHX38	CDK5RAP1	IDUA	IRAK1	ORMDL3
GPAA1	B3GALT6	XPC	LIN7C	VWA1	MET
METTL1	COA4	CTIF	SHPK	PNKP	

(iii) Proteins enriched more than two-fold in c-MYC-WT compared with c-MYC-ΔMB0 (EpiP)					
FAM83G	IFT20	DOP1B	NOC3L	POLR2F	EXOC8
STK25	ASPM	WIPI2	LSAMP	KHNYN	S100A3
BTAF1	GPD1L	OARD1	ARF1	HACD2	VWA1
URB1	STAG2	SLC25A15	SYF2	RIDA	METTL1
PCDHGA12	CCNYL1	IKBKG	GXYLT1	CEP250	SMARCA4
TGFB1	NECAP1	MAP3K4	NLE1	RBM19	PRIM2
SERPINE2	PPHLN1	UTP3	PTBP3	LMF2	SHPK
SP1	STAG1	CD320	YTHDF2	ORC2	F8A1,F8A2,F8A3
GLUL	SREK1	ARMC9	C7orf50	MRPL17	BMI1
PHKG2	COX6A1	RPL36A	DNMT1	MARS2	C8orf33
NF1	BRI3BP	LAS1L	FAM162A	PTCD1	CCDC93
BRD2	LPIN2	PLA2G4A	HMGN5	CEPT1	MET
ATP6V0C	ZNF622	PEG10	CDK2AP1	LIMD2	COA4
NCAPD3	SNX21	CCDC58	NUP50	ORC4	TTC5
SSR2	NUDT16	LYRM7	SLC16A1	LAGE3	CCDC63
PRIM1	SAAL1	WARS2	ERCC4	RMC1	PODXL2
GTF2A2	DTD2	ZNF24	UTP4	NOL10	BLOC1S4
EPHB3	WASF2	NAA16	BUD23	POLD3	CDK5RAP1
UBE2G2	CCDC115	BOP1	SELENBP1	H2AC21	SPATA5L1
PLCB3	HAPLN3	TNFRSF10B	SNCA	CNBP	XPC
ATP7A	DNAJC2	ORC5	VPS33A	CAMLG	MPC1
PTK2	AKAP9	HARS2	CTSC	DOCK1	MIPEP
HSPA14	THTPA	TIMM17A	INTS13	SPR	PVR
ATR	CEP41	PABPN1	REXO4	CSNK2A1	AKAP1
VAMP3	XPO4	NOLC1	PDF	CCDC51	SRC
PFKFB3	CSTF2T	SMN1,SMN2	APOO	CLPB	AFP
DOCK11	QRSL1	WRNIP1	MSH3	YY1	RAVER2
ZFYVE27	TTC12	HDDC3	IGFBP5	HEATR5A	CRLF3
RNASEH2B	CARS2	STARD4	SMYD3	WDHD1	DDX60
SH3PXD2A	C17orf75	GCA	RPL10L	NSD2	HIGD1A
TNS2	CRLF2	TRIM65	INTS11	MDP1	EXOG
ATG16L1	TNFRSF12A	PBX1	IMP3	GLE1	POLRMT
TTI2	INCENP	UQCC1	AATF	MTMR6	MRPS30
ULK3	INTS9	NDUFAF1	MRPS15	THNSL1	RRP1
TTC33	MTRR	EHMT2	LIG1	RBFOX2	CDC16
KDM3B	TRAP1	HSPD1	POLA2	PKMYT1	KIF21B
TTC21B	PTBP2	UBR5	CCDC171	MAP3K7	NFATC2IP
HEATR3	PALD1	CDKN2AIPNL	TGS1	RSL1D1	TERF2
ZC3HC1	ISY1	NAF1	BAZ1A	FANCI	RIF1
VRK2	SYNPO2	PRORP			

(iv) Commonly enriched proteins in (i) and (ii) and (iii)					
KDM3B					
HMGN5					
URB1					
PALD1					
LSAMP					
MET					
DNAJC2					
RBM19					
AKAP1					
INTS9					
SNCA					
MARS2					
MAP3K4					
INCENP					
AKAP9					
GCA					
NCAPD3					
FAM162A					
ORC5					
ULK3					
POLRMT					
ZC3HC1					
PTCD1					
NOL10					
SHPK					
XPC					
SH3PXD2A					
RAVER2					
PTBP3					
BOP1					
PEG10					
TIMM17A					
SAAL1					
CARS2					
FANCI					
HARS2					
BAZ1A					
PLA2G4A					
APOO					
WARS2					
TRAP1					
RSL1D1					

Four groups are described: (i) proteins whose peptide counts increased more than two-fold in c-MYC-WT/HDFs compared with MYCL-WT/HDFs using SeV on day 3, 5, or 7; (ii) proteins whose peptide counts increased more than two-fold in c-MYC-WT compared with MYCL-WT using EpiP; (iii) proteins whose peptide counts increased more than two-fold in c-MYC-WT compared with c-MYC-ΔMB0 using EpiP; and (iv) commonly identified proteins. *n*=3 for EpiP reprogramming.

**Table 3:** MS analysis of identified proteins in cells reprogrammed with MYCL-WT and c-MYC-ΔMB0 compared with MYCL-ΔMB0.

(i) Proteins enriched more than two-fold in MYCL-WT compared with MYCL-ΔMB0 (EpiP)					
UBQLN2	DYNLRB1	EXOC2	KRT17	NANS	ARL1
REEP5	RAB11B	ARPC2	TPM1	UTP15	TMPO
SGCD	WDR46	CD47	SLC44A2	RASA1	CETN2
TUBB2A	ALDH6A1	STRN3	FBXW10	BAP18	SNW1
HMGB1	POTEF	PDHA1	CAMK2D	TM9SF3	NFS1
PPIC	COMMD4	SCP2	MT-ATP6	YIPF5	MPI
HMGB3	TUSC3	COL3A1	ARF4	ATP5PB	MPDU1
PCNP	MYD88	GNB2	NUCKS1	LIMS1	USP15
SYAP1	RAC1	COG6	TMED2	LRBA	OVCA2
CHMP4B	MACF1	SOX2	PRKACA	MAPK14	CALD1
UTP3	SRSF11	SSR1	MID1	DUSP12	GBP1
BLOC1S1	EXOC5	CSTF3	PCID2	THOC3	ANP32B
ST6GALNAC1	DNAJC9	CACNA2D1	TRIP12	SRSF5	USP48
MAP3K20	MICAL1	NRDC	GADD45GIP1	VAC14	PDIA4
KNTC1	POLR2L	MSRB3	GTF2I	PDXDC1	SF3A3
BAG5	SFXN3	NOL11	ERLIN2	ZNF462	NUCB2
CD320	CRIP1	OSTC	DBNL	ITGB1	AGK
MRPL11	NAA10	RPL37A	STT3A	CTTN	PPIL3
TFG	ARF6	LZIC	PAFAH1B1	EEF1B2	IDI1
THYN1	CCDC43	BMP1	SLC25A24	UGP2	ELN
NDRG3	NEK7	PDCL3	HOOK3	LSM2	COL4A2
TMX4	TUBB4B	UGP2	LAS1L	ACTB	BUD31
SEPHS1	MYO1E	TNS3	HLA-H	RABL3	MAP2
RPL36A	FNTA	VPS26B	DCN	RWDD1	PAIP1
MYDGF	SRBD1	EHD1	PUM1	TOMM20	ITGA5
OPTN	DBI	ANKFY1	PUS7	CRABP2	GPX8
GNAQ	SUGP2	KBTBD3	KPNA4	VDAC3	TXLNG
ACSL4	MTA1	SCPEP1	METTL26	EDIL3	ATP5MG
ATP5ME	MAP7D1	FBLN2	B4GALT4	PLA2G4A	PIK3R4
ABI3BP	ACTG1	LDLR	MBD5	CTNNA1	PLBD2
ASAH1	HINT1	EXOSC7	CSRP1	RPL23A	ZYX
GNS	HMGN1	DIP2B	GNB1	TMEM165	COX7C
WDR61	PTGR1	PITRM1	SNRPB2	DNAH6	H3-3A,H3-3B
ARL8A	TMSB4X	METTL14	CNN2	DPP9	NCKAP1
MAP3K20	FAM114A1	TMSB10	PPIB	ENDOD1	AHNAK
NDRG1	FTH1	CNPY3	S100A10	NDUFB11	PGM2
PITPNA	SGTA	HABP2	C1orf198	NAA50	PODXL
NIF3L1	SGPL1	SRP9	MARCKSL1	DNAJC8	CFL2
NME2	CD59	NDUFB9	TOR1AIP1	NXN	STAT6
PFDN1	DHRS4	RBPJ	NDUFA4	MRPS17	TP53BP1
ATG3	GSPT1	DCTN5	ACSS2	REXO2	ATAD1
ACIN1	BLOC1S3	TMED1	GSTK1	PEBP1	EIF3K
RAB14	RFC3	AKR1B1	ISLR	S100A13	GPX7
SNAP23	CD55	TALDO1	NOP14	SLC25A6	TRIO
EMC2	RPS15A	DSTN	POLR2A	OSBPL3	TSPYL5

(ii) Proteins enriched more than wo-fold in c-MYC-ΔMB0 compared with MYCL-ΔMB0 (EpiP)					
UBQLN2	ACIN1	NEK7	PITRM1	PDLIM5	CAMK2D
HMGB1	SRSF11	RAB11B	ITGA1	H3-3A,H3-3B	CRABP2
REEP5	OPTN	ANKFY1	ISLR	C1orf198	NCKAP1
NDRG1	KNTC1	CD47	SRSF5	LUZP1	HMGN1
SGCD	PAIP1	PRKACA	ERLIN2	IDI1	MAP2
MAP3K20	NIF3L1	SCRN3	MRPS17	HABP2	TOR1AIP1
TUBB2A	POTEF	FAM114A1	TOMM20	PAFAH1B1	ARL1
CCDC43	COG6	NOLC1	UGP2	ITGA5	OSBPL3
PCNP	TSPYL5	COMMD4	LSM2	CHIC1	ANP32B
TMX4	DYNLRB1	EHD1	UTP15	SMTN	STT3A
BLOC1S1	STRN3	PPP4C	PUM1	DUSP12	VPS26B
SYAP1	ASAH1	FNTA	MYO1E	MEAF6	APP
PPIC	ATP5ME	GNAQ	TP53RK	NMT1	GALNT1
NME2	OVCA2	SEMA7A	POLR2L	NDUFA4	SSR1
ACSL4	RAC1	RPL36A	MYO6	LIMS1	YIF1A
HMGB3	OSTC	FTH1	PUS7	CLASP2	UGP2
FBXW10	DBI	PTGR1	PIK3R4	ILF3	USP48
MAP3K20	PDHA1	KRT17	MYD88	RASA1	PPIB
NDRG3	SFXN3	ARPC2	REXO2	GALE	METTL26
GNS	TNS3	ABI1	DBNL	TXLNG	MYO1D
UTP3	DHRS4	PAIP1	USP15	PPIL3	NDUFS6
SEPHS1	MPDU1	PCID2	SRBD1	RFC3	PPIF
BAG5	DNAJC9	SEC24A	ACTG1	SCLY	SMARCC2
ATG3	MAPK14	UBQLN1	LZIC	MPV17	STAM2
SCPEP1	MRPS24	MTA1	TRIP12	VAC14	BMP1
TFG	PLA2G4A	BAP18	THOC3	DCTN5	PGM2
MT-ATP6	NOL11	METTL14	SCP2	COG7	CERS2
MRPL11	SUGP2	EXOC5	POLR2A	MCRIP1	SLIT3
CHMP4B	TUSC3	BLOC1S3	CSTF3	CTNNA1	HOOK3
SGPL1	DIP2B	DUSP23	GNB2	SENP3	COL3A1
MICAL1	PFDN1	SOX2	GSPT1	EIF2B1	ITGB1
DCN	NRDC	LDLR	GADD45GIP1	SETD7	AGK
CUL4A	SGTA	CNPY3	KPNA4	DNASE2	MPHOSPH10
ARF6	PODXL	MPI	FADS2	RAI14	BOLA2,BOLA2B
HLA-H	ARL8A	HINT1	NSA2	TPMT	IMPACT
MACF1	PDXDC1	GSTK1	ATAD1	EDIL3	USP9X
THYN1	CD320	CD59	METAP1	IGF2R	OCIAD2
MYDGF	PITPNA	FBLN2	TPM1	NAA50	CACNA2D1
NACA4P	MAP7D1	NANS	MBD5	EEF1B2	YIPF5
NAA10	WDR46	TM9SF3	DCTD	EEA1	TMSB10
NDUFB9	LRRC17	YAP1	GTF2I	CD55	SNAP23
ABI3BP	WDR61	RABL3	PRKRA	DNAJC8	EIF3H
SNW1	LMCD1	KLF4	PSMD4	S100A10	KBTBD3
SNRPB2	COL4A2	CFL2	LSM5	FBXO22	ACYP1
ARF4	VDAC3	ALDH6A1	CYP51A1	CBX3	SNX27
ATP5MG	CNN1	CLINT1	ACSS2	EIF2B3	SLC25A24
CTTN	CALD1	DYNC1LI1	NDUFAF2	S100A13	CDC42BPB
TMPO	EWSR1	ANTXR2	TP53BP1	RBM17	IDH3G
RPL37A	SPATS2L	PHLDB1	CARHSP1	CFAP74	EMC2
PLS1	HSD17B7	FAP	NXN	TUBB4B	SF3A3
MTR	OSTF1	TMSB4X	SRP9	VASP	EXOC2
NCBP1	SFXN4	NUCKS1	RPS29	MANF	COPS6
VPS4B	AHNAK	CSRP1	SLC25A6	CARM1	FKBP2
TMOD3	RBPJ	TALDO1	ATP5PB	PRPF4B	TBC1D15
GPX7	B4GALT4	MSRB3	DNAH6	COX7C	OGFR
ALDH1L2	CRIP1	AP3M1	SCAMP2	C1orf50	ELN
TSN	TOR1B	PDS5A	PDIA4	RWDD1	GABPA
DYNLL1	USP47	ENDOD1	NUCB2	GINS4	DR1
FKBP5	OAS2	MRTO4	NFS1	NPC2	ABI2
SERPINE2	NDUFAF4	ZYX	HACD3	EXOSC7	

(iii) Commonly enriched proteins in (i) and (ii)					
WDR46	TUSC3	RBPJ	NFS1	NIF3L1	DNAJC8
MYO1E	MYD88	FBXW10	ARF4	TM9SF3	TMPO
RAC1	SFXN3	CAMK2D	MPDU1	CTNNA1	EEF1B2
TMSB10	MACF1	NUCKS1	OVCA2	CHMP4B	COL3A1
LDLR	DBNL	PRKACA	ANP32B	PCNP	S100A10
CRABP2	EXOC5	TRIP12	SLC25A24	PAIP1	NEK7
NOL11	DNAJC9	GADD45GIP1	SF3A3	ARL1	MICAL1
POLR2L	CRIP1	GTF2I	AGK	PODXL	S100A13
TOMM20	CCDC43	AHNAK	PPIL3	REXO2	STT3A
CFL2	SRBD1	ENDOD1	IDI1	FNTA	H3-3A,H3-3B
POTEF	SUGP2	PDHA1	ELN	FBLN2	SCPEP1
TSPYL5	MTA1	HOOK3	COL4A2	NDRG3	TXLNG
SGTA	TMSB4X	HLA-H	MAP2	EXOC2	MPI
REEP5	ACTG1	PUM1	ATP5MG	SOX2	HMGN1
SGCD	HINT1	KPNA4	PIK3R4	DBI	UBQLN2
HMGB3	PTGR1	METTL26	COX7C	TUBB2A	NDUFB9
SYAP1	EMC2	B4GALT4	NCKAP1	SRP9	PDXDC1
UTP3	RPL36A	MBD5	ASAH1	FAM114A1	TOR1AIP1
BLOC1S1	CD59	SNRPB2	PGM2	CD55	MAP7D1
MAP3K20	NUCB2	C1orf198	TP53BP1	SNAP23	COG6
KNTC1	BLOC1S3	ACSS2	ATAD1	UGP2	TNS3
BAG5	STRN3	GSTK1	RPL37A	KRT17	SGPL1
CD320	SCP2	NANS	RFC3	DCTN5	CSRP1
TFG	GNB2	UTP15	TALDO1	USP48	ATG3
THYN1	SSR1	BAP18	ISLR	NDUFA4	EDIL3
TMX4	CSTF3	YIPF5	POLR2A	ITGB1	ZYX
ARL8A	CACNA2D1	LIMS1	OSBPL3	ATP5PB	PCID2
GNAQ	NRDC	THOC3	GPX7	PUS7	RASA1
ACSL4	MSRB3	SRSF5	PPIC	SRSF11	DNAH6
ATP5ME	OSTC	VAC14	MYDGF	USP15	ALDH6A1
ABI3BP	LZIC	ARF6	HMGB1	MT-ATP6	COMMD4
GNS	BMP1	UGP2	ERLIN2	NAA10	CNPY3
MAP3K20	VPS26B	LSM2	FTH1	ITGA5	HABP2
NDRG1	ANKFY1	RABL3	DUSP12	CD47	DHRS4
NME2	KBTBD3	RWDD1	CTTN	MRPL11	SNW1
PFDN1	EHD1	PLA2G4A	PPIB	OPTN	ARPC2
MAPK14	EXOSC7	SLC25A6	SEPHS1	WDR61	TPM1
ACIN1	DIP2B	NAA50	DCN	PDIA4	GSPT1
DYNLRB1	PITRM1	NXN	PAFAH1B1	VDAC3	TUBB4B
RAB11B	METTL14	MRPS17	PITPNA	CALD1	

Three groups are described: (i) proteins whose peptide counts increased more than two-fold in MYCL-WT compared to MYCL-ΔMB0; (ii) proteins whose peptide counts increased more than two-fold in c-MYC-ΔMB0 compared to MYCL-ΔMB0; and (iii) commonly identified proteins. *n* = 3.

We also compared phosphorylated proteins during SeV reprogramming with MYCL and c-MYC. In total, there was more than a two-fold relative increase of 17 phosphorylated proteins with MYCL-WT and 132 phosphorylated proteins with c-MYC-WT. The GO analysis indicated that the phosphorylated proteins increased by MYCL included cytoskeleton-related proteins and those increased by c-MYC included transcription-related proteins (Supplementary Fig. S11).

### MYCL regulates RNA processing-related proteins during reprogramming via the MB2 domain

**Figure 4:**
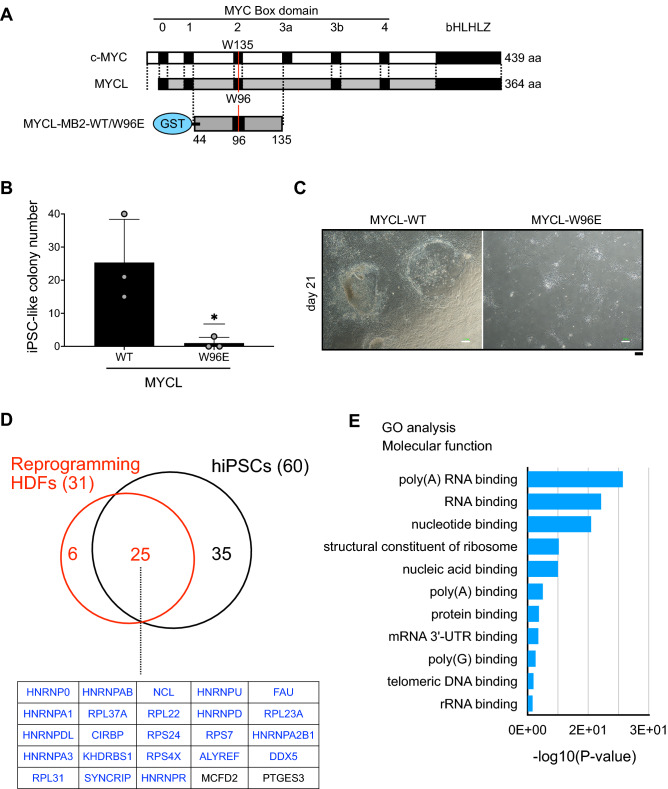
MYCL regulates RNA processing-related proteins during reprogramming via the MB2 domain. (**A**) W96 and W135 in the MB2 domain of MYCL and c-MYC, respectively. The structure with the recombinant protein of the MB2 domain of MYCL-WT/W96E is shown below. The numbers on the right indicate amino acid lengths. (**B**) The number of iPSC-like and non-iPSC-like colonies derived from 1 × 10^5^ HDFs transduced with EpiP including MYCL-WT or MYCL-W96E on day 21. Mean ± SD values are shown. *n* = 3, *p < 0.05 by unpaired *t*-test. (**C**) Representative images of reprogramming HDFs 21 days after the transduction of EpiP, including MYCL-WT or MYCL-W96E. Scale bars, 100 μm. (**D**) Venn diagram of enriched proteins between reprogramming HDFs and hiPSCs by AP-MS. A list of the 25 commonly enriched proteins is shown below. Blue indicates RBP (23 in total). (**E**) Molecular function from the GO analysis of the 25 commonly identified proteins in (**D**).

Our analysis also revealed that, along with the MYCL MB0 domain, the MYCL MB2 domain is important for reprogramming (Fig. 2B). It has been reported that the c-MYC MB2 domain is involved in transformation activity, and tryptophan 135 within the MB2 domain is necessary for this activity^10^. MYCL also has a tryptophan residue within its MB2 domain but little transformation activity^23^. We hypothesized that this domain in MYCL has reprogramming function. We therefore produced a mutant in which tryptophan 96 was substituted with glutamate (W96E). This tryptophan is equivalent to tryptophan 135 in c-MYC (Fig. 4A and Supplementary Fig. S5B). We confirmed the expression of MYCL-W96E by western blotting (Supplementary Fig. S12). Next, we examined the effect of MYCL-W96E for reprogramming. HDFs were transfected with reprogramming factors including MYCL-WT or -W96E. MYCL-W96E could not induce iPSC-like colonies, suggesting tryptophan 96 is crucial for reprogramming (Fig. 4B, C). We thus hypothesized that the residue might be important for MYCL to bind to other proteins. To identify the binding proteins, we produced GST-fusion recombinant proteins of the MYCL MB2 domain (Fig. 4A). GST-MYCL-MB2-WT or -W96E proteins were immobilized on glutathione Sepharose, and affinity columns were prepared. Cell lysates were applied to the column, and, after washing, the bound proteins were eluted. We used the cell lysates from reprogramming HDFs, but since it was difficult to collect a large amount, we also used cell lysates from hiPSCs. The reason for using the hiPSC lysates is that many of the proteins expressed in reprogramming HDFs are highly expressed in hiPSCs as well^16,24–27^.

We identified 31 candidate proteins that bind to the MB2 domain of MYCL-WT but not of MYCL-W96E during reprogramming in the HDF lysates (Fig. 4D and Table 4). Of those 31 proteins, 25 proteins were also identified using hiPSC lysates, and 23 were RNA-binding proteins (RBPs; Fig. 4D, genes written in blue). Six proteins were identified only in the reprogramming HDFs lysates: HNRNPK, DDX17, C1QBP, KBTBD3, COPG2, and SIKE1, of which HNRNPK, DDX17, and C1QBP are RBPs. From these results, there were 26 RBPs identified in the HDF lysates in total. We confirmed the function of the 31 proteins using a public database (https://www.nextprot.org/)^28^ and found 16 of them are involved in RNA processing. A GO analysis using DAVID also showed that the 31 proteins are related to controlling pre-mRNA splicing, capping, and polyadenylation, suggesting functions in mRNA export, turnover, localization, and translation (Fig. 4E). These results suggested that MYCL interacts with RBPs via its MB2 domain and promotes reprogramming by post-transcriptional regulation.

**Table 4:** AP-MS analysis of identified proteins in MYCL-MB2-WT using cell lysates from reprogrammed HDFs and hiPSCs.

(i) Proteins enriched more than two-fold in MYCL-MB2-WT compared with MYCL-MB2-W96E (reprogramming HDFs) (31)					
***HNRNPA1***	***HNRNPD***	***HNRNPDL***	***HNRNPA2B1***	***HNRNPA0***	***HNRNPA3***
***KHDRBS1***	***HNRNPAB***	***ALYREF***	***DDX5***	***HNRNPU***	***SYNCRIP***
***HNRNPR***	***HNRNPK***	***DDX17***	***C1QBP***	*FAU*	*RPL22*
*RPL37A*	*RPL23A*	*NCL*	*RPS24*	*RPL31*	*RPS4X*
*RPS7*	*CIRBP*	MCFD2	KBTBD3	COPG2	PTGES3
SIKE1					
	Italic value	RNA binding proteins			
	Bold italic value	: RNA processing proteins			

(ii) Proteins enriched more than two-fold in MYCL-MB2-WT compared with MYCL-MB2-W96E (hiPSCs) (60)					
***HNRNPA1***	***HNRNPD***	***HNRNPDL***	***HNRNPA2B1***	***HNRNPA0***	***HNRNPA3***
***KHDRBS1***	***HNRNPAB***	***ALYREF***	***DDX5***	***HNRNPU***	***SYNCRIP***
***HNRNPR***	***HNRNPK***	***PRPF31***	***DDX42***	***LYAR***	***YBX1***
*FAU*	*RPL22*	*RPL37A*	*RPL23A*	*NCL*	*RPS24*
*RPL31*	*RPS4X*	*RPS7*	*CIRBP*	*RPL23*	*RPL30*
*LSM14A*	*DDX18*	*NSUN2*	*ILF3*	*TRMT1*	*SRP9*
*IGF2BP1*	*SRP14*	*ANP32A*	*ILF2*	HACD3	UBE2N
GPC4	MDK	BAX	ANP32E	ANP32B	APEX1
PTGES3	MCFD2	CFDP1	ACTR2	ITGB1	SFRP1
DSTN	PDHB	HADHA	HADHB	NDUFS5	CNBP
	Italic value	RNA binding proteins			
	Bold italic value	: RNA processing proteins			

(iii) Common enriched proteins enriched between reprogramming HDFs and hiPSCs (25)					
***HNRNPA1***	***HNRNPD***	***HNRNPDL***	***HNRNPA2B1***	***HNRNPA0***	***HNRNPA3***
***KHDRBS1***	***HNRNPAB***	***ALYREF***	***DDX5***	***HNRNPU***	***SYNCRIP***
***HNRNPR***	*FAU*	*RPL37A*	*RPL22*	*RPL23A*	*CIRBP*
*RPS24*	*RPS7*	*RPS4X*	*RPL31*	*NCL*	PTGES3
MCFD2					
	Italic value	RNA binding proteins			
	Bold italic value	: RNA processing proteins			

Three groups are described: (i) protein interactors whose peptide counts increased in reprogramming HDFs more than two-fold in MYCL-MB2-WT than MYCL-MB2-W96E; (ii) protein interactors whose peptide counts increased in hiPSCs more than two-fold in MYCL-MB2-WT compared to MYCL-MB2-W96E; and (iii) commonly identified proteins. *n* = 1.

## Discussion

Here we described the molecular function of MYCL during reprogramming and compared it to the c-MYC function by focusing on MYC Box domains. We found that the MB0 and MB2 domains are important for reprogramming, and deleting either region compromised the reprogramming ability of MYCL. Proteomic analysis revealed that MYCL regulates the expression of cell adhesion-related proteins during reprogramming via the MB0 domain (Fig. 3C, D). We also found the possibility that the same domain is regulated by post-translational modifications (PTM), as discussed below. It is known that cell-substrate adhesion is closely related to the mesenchymal-epithelial transition (MET)^29^ and that MET occurs during the reprogramming process^30–32^. We speculate that MYCL promotes iPSC-like colony formation via the MET process by upregulating cell adhesion-related genes. Furthermore, we identified that the MB2 domain is required for MYCL to promote reprogramming by binding to RBPs, especially RNA processing-related proteins (Fig. 4D, E). It has been reported that RBPs regulate MET through post-transcriptional regulation. For example, heterogeneous nuclear ribonucleoprotein (hnRNP) A1 regulates the alternative splicing of Rac1 to control MET^33^. These findings suggest that MYCL regulates the RNA processing of cell adhesion-related genes transcribed by MYCL itself or other genes. Therefore, we hypothesize that transcriptional and post-transcriptional regulation by MYCL promotes MET, which increases the efficiency of reprogramming and leads to higher quality iPSCs.

Western blotting revealed that MYCL protein has a unique expression pattern (Supplementary Fig. S8 and S12). The calculated molecular weight of MYCL is about 40 kDa (364 aa), but we detected three strong bands at around 60 kDa, which we verified with second antibody (Supplementary Fig. S13). Since the expression of MYCL-ΔMB0 showed a strong single band, we speculate that the MYCL MB0 domain is the PTM site (Supplementary Fig. S8). Such a phenomenon was not observed in c-MYC (Supplementary Fig. S7). One possible type of relevant PTM is phosphorylation. Phosphorylation is crucial for protein function. For example, RNA polymerase II (Pol II) is required for transcription pauses in a promoter-proximal position during transcription initiation. In order to initiate transcription, the C-terminal domain of Pol II must be phosphorylated by P-TEFb^34^. In addition, the phosphorylation of c-MYC on threonine 58 in the MB1 domain promotes c-MYC binding to F-box and WD repeat domain containing 7 (FBXW7), causing the ubiquitination of c-MYC, which triggers c-MYC degradation^35^. Similarly, MYCL might undergo phosphorylation to change its activity and interaction with binding proteins. However, this hypothesis requires further study.

Comprehensive proteomic analysis suggested that the MYCL MB0 domain influences the expression of cell adhesion-related proteins, and MYCL shows an up-regulation of phosphorylated cytoskeletal proteins (Fig. 3C, D, and Supplementary Fig. S11A). Cell adhesion is mediated by adhesion molecules, such as integrins and cadherins, which function in the extracellular matrix (ECM) and cell–cell adhesion and are important for cell communication and the regulation of fundamental physiological processes such as tissue development and maintenance^36,37^. Human iPSCs and hESCs have unique focal adhesion localization, and appropriate adhesion to the ECM is required to regulate reprogramming via MET and maintain pluripotency^38–40^. Accordingly, our study supports MYCL regulating cell-substrate adhesion through its MB0 domain to promote reprogramming. In other words, MYCL might regulate proteins involved in cell adhesion and the cytoskeleton directly or indirectly to cause MET and promote reprogramming. In c-MYC, loss of the MB0 domain positively affects iPSC-like colony formation, suggesting that this domain has a different function compared to MYCL. This functional difference is somewhat surprising since the domain is well conserved (Supplementary Fig. S5B). We would like to clarify this point in the future.

**Figure 5:**
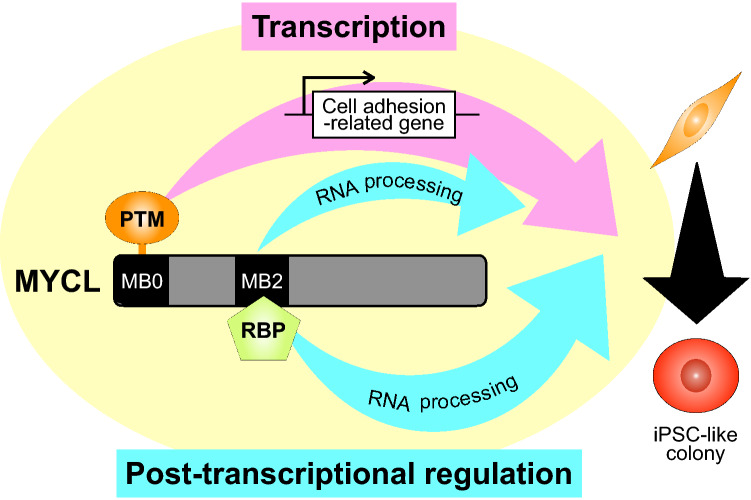
Model of the reprogramming process by MYCL. MYCL promotes iPSC-like colonies via its MB0 and MB2 domains. The MB0 domain regulates the expression of cell-adhesion proteins, possibly via post-translational modifications (PTM). The MB2 domain regulates RNA processing by interacting with RNA-binding proteins (RBP). We speculate that MYCL promotes reprogramming through the synergistic effects of these two mechanisms.

We also found that the MB2 domain has an important function in MYCL-reprogramming (Fig. 2B,C). Deleting the MB2 domain completely compromised the reprogramming ability of MYCL. In c-MYC, the MB2 domain has an important function in transformation activity^14^, and tryptophan 135 in the MB2 domain is essential for this activity. The equivalent tryptophan residue in MYCL is tryptophan 96. MYCL has little transformation activity, but we showed that the mutation of tryptophan 96 completely lost the reprogramming ability of MYCL. To further investigate the function, we sought interacting proteins by affinity column chromatography. We found 31 proteins, including 26 RBPs, that interact with the MYCL MB2 domain (Table 4, genes written in blue). A GO analysis suggested that some of the 31 proteins are involved in RNA processing (Table 4). It has been reported that altered RNA processing affects somatic cell reprogramming^41^. Therefore, we hypothesize that MYCL also promotes MET in reprogramming by regulating RNA processing via interactions with RBPs at its MB2 domain. An illustrative summary of how MYCL regulates cell reprogramming through these two domains is shown in Fig. 5.

To conclude, we have demonstrated that MYCL promotes more efficient reprogramming than c-MYC, regulates the expression of cell adhesion and cytoskeletal proteins, and is involved in RNA processing via a single tryptophan residue in the MB2 domain. Following these findings, we propose that MYCL causes MET by regulating the expression of proteins involved in the promotion of reprogramming from the RNA-processing stage. Further elucidation of the function of MYCL in reprogramming will improve the quality and efficiency of iPSC generation.

## Material and methods

### Cell culture

HDFs (106-05f.) were purchased from Cell Applications, Inc. HDFs were cultured in DMEM (08459-64, Nacalai Tesque) supplemented with 10% FBS (10439-024, gibco) and 1% penicillin and streptomycin (15140-122, Pen/Strep, gibco). The hiPSC clone 201B7 was used in this study^2^. iPSCs were cultivated on iMatrix-511 (NP892-012, Nippi)-coated (0.5 μg/cm^2^) cell culture plates with StemFit (AK03N, Ajinomoto) supplemented with bFGF and passaged via dissociation into single cells using TrypLE Select (A12859-01, Life Technologies) on day 7 following a previously reported protocol^42^.

### Generation of iPSCs

A frozen stock of HDFs was thawed and cultured for four days, and then 1 × 10^5^ cells were collected by trypsinization. With SeV, HDFs were transduced with the CytoTune-iPS 2.0 (c-MYC) or CytoTune-iPS 2.0L (MYCL) Sendai Reprogramming Kit (DV-0304, DV-0305, ID Pharma). With EpiP, HDFs were electroporated with 1.2 μg of plasmid mixtures with the Neon Transfection System (MPK1096 and MPK10096, Invitrogen). The plasmid mixtures included pCXLE-SOX2, -KLF4, -OCT3/4-shp53, -LIN28A, and pCXWB-EBNA1 with wild-type or mutant pCXLE-c-MYC or -MYCL^17^. The mixing ratio of SOX2, KLF4, OCT3/4-shp53, LIN28A, EBNA1, and c-MYC/MYCL was 1:1:2:1:0.5:2. After that, the cells were plated in a 6-well plate and cultured in StemFit AK03N without bFGF with iMatrix-511 at 0.25 μg/cm^2^ in SeV or 0.125 μg/cm^2^ in EpiP. The culture medium was changed the next day and every three days after that. The colonies were counted 21 days after plating.

### Episomal plasmid vector construction for deletion mutants of c-MYC and MYCL

We previously generated pCXLE-c-MYC and -MYCL from human cDNAs encoding c-MYC and MYCL amplified by PCR and cloned into pENTR1A^17^. Primers for the deletion mutants were designed using the Primer Design tool for the In-Fusion HD Cloning Kit (639650, Takara) and inserted into pENTR1A. The switch from pENTR1A to pCXLE was done using the Gateway system (11791020, Invitrogen). The primers used are listed in Table S1.

### Immunostaining

The cells were fixed with 4% formaldehyde (163-20145, Wako) for 20 min at room temperature. Then, the fixed cells were treated with PBS (14249-24, Nacalai Tesque) containing 0.5% Triton X-100 (35501-15, Nacalai Tesque) and 3% bovine serum albumin (01281-84, BSA, Nacalai Tesque) for 20 min at room temperature for permeabilization. The cells were incubated with primary antibodies diluted in PBS containing 3% BSA at 4℃ overnight. After washing with PBS, the cells were incubated with fluorescence-conjugated secondary antibodies for 1 h at room temperature. Nuclei were visualized with Hoechst 33342 (346-07951, DOJINDO). Anti-TRA-1-60 (1:500, 560071, BD Pharmingen, and 1:500, 09-0068, Stemgent) and Alexa 488-conjugated goat anti-mouse IgG, IgM (H + L) (1:250, A10680, Invitrogen) were used as the antibodies.

### Imaging and quantification

Stained cells were imaged using a BZ-9000 imaging system (KEYENCE) or ArrayScan High-Content Systems (Thermo Fisher Scientific). HCS Studio 2.0 Cell Analysis Software (Thermo Fisher Scientific) was used to quantify cell counts and signal intensities. The Cellomics BioApplication system (Thermo Fisher Scientific) was programmed to capture and analyze 25 images per well. The total cell number was detected by Hoechst 33342 staining. The number of TRA-1-60 ( +) cells was calculated as the number of TRA-1-60 ( +) cells among Hoechst ( +) cells. TRA-1-60 ( +) cells were calculated by dividing this number by the total cell number.

### Flow cytometry

Transduced cells were harvested with 0.25% trypsin/1 mM EDTA (25200-056, gibco) each day after the transduction for the analysis. At least 5 × 10^4^ cells were stained with the following antibodies in FACS buffer (2% FBS, 0.36% glucose (16806-25, Nacalai Tesque), 50 μg/μL Pen/Strep in PBS) for 30 min at room temperature: BV510-conjugated anti-TRA-1-60 (1:40, 563188, BD Biosciences) and PE-Cy7-conjugated anti-CD13 (1:40, 561599, BD Biosciences) antibodies. The analysis was performed using MACSQuant Analyzers (Miltenyi Biotec). Negative controls used a mixture of HDFs without any EpiP transduction and reprogramming HDFs electroporated with EpiP including c-MYC or MYCL. “Isotype” means mixed HDFs stained with the isotype control of anti-TRA-1-60 (1:40, 563082, BD Biosciences) and -CD13 (1:40, 557646, BD Biosciences) antibodies.

### SDS-PAGE

Cells were lysed with SDS sample buffer (0.125 M Tris-base (35434-21, Nacalai Tesque), 0.96 M glycine (17109-35, Nacalai Tesque), and 17.3 mM SDS (31606-75, Nacalai Tesque)) containing 3-mercaptoethanol (139-16452, Wako). Samples were applied and separated in an 8% polyacrylamide gel composed of 30% (w/v)-Acrylamide/Bis Mixed Solution (29:1) (06141-35, Nacalai Tesque), Separating Gel Buffer Solution (4x) (30651-05, Nacalai Tesque) and Stacking Gel Buffer Solution (4x) (32158-25, Nacalai Tesque) for SDS-PAGE.

### Western blotting

Proteins on an SDS-PAGE gel were transferred to a PVDF membrane (IPVH00010, Immobilon-P, Millipore) and probed with the following antibodies using an iBind Flex system (SLF2000, SLF2010 and SLF2020, Invitrogen): anti-human MYCL (1:250, AF4050, R&D) (1:250, C-20, sc-790, Santa Cruz), anti-human c-MYC (1:500, 9E10, sc-40, Santa Cruz, and 1:500, D84C12, CST), anti-β-actin (1:1000, A5441, SIGMA), anti-Goat (1:3000, ab6741-1, abcam), anti-mouse (1:3000, 7076S, CST), and anti-rabbit (1:3000, 7074S, CST) antibodies.

### Preparation of recombinant proteins and affinity purification (AP)

The MB2 region of MYCL-WT or -W96E was cloned into pGEX-6P-1. The plasmids were transformed into BL21 *E. coli* (DE3) (L1198, Promega) competent cells. The fusion proteins, GST-MYCL-WT-MB2 and GST-MYCL-W96E-MB2, were induced by treatment with 0.5 mM IPTG (19742-94, Nacalai Tesque) for 4 h at 37 °C. The proteins were purified using glutathione Sepharose beads (17-0756-01, GE Healthcare). Human iPSCs or reprogramming HDFs were lysed in RIPA buffer (20 mM Tris/HCl (pH 7.6) (35436-01, Nacalai Tesque), 1% NP-40 (25223-75, Nacalai Tesque), 0.1% SDS, 150 mM NaCl (31320-05, Nacalai Tesque), and protease inhibitor (25955-11, Nacalai Tesque)) and then centrifuged. Cell lysates (supernatant) were transferred into a column (29922, Thermo Fisher Scientific) packed with beads conjugated with GST- MYCL-WT or -W96E proteins. After washing, binding proteins were eluted in lysis buffer (12 mM sodium deoxycholate (190-08313, Wako), 12 mM sodium lauroyl sarcosinate (192-10382, Wako), and 100 mM Tris-HCl (pH9.0) (314-90381, NIPPON GENE)) for the MS analysis. The iPSC lysates were prepared 6 days after passaging in two 10-cm dishes (*n* = 1), and reprogramming HDF lysates were prepared 3 days after SeV transduction in five 10-cm dishes (*n* = 1).

### GO analysis by DAVID

The Database for Annotation, Visualization, and Integrated Discovery (DAVID Bioinformatics Resources 6.8) was used to identify enriched biological GO terms and KEGG pathway^43–45^. For more information, please visit the DAVID website (https://david.ncifcrf.gov/home.jsp) and KEGG Database website (https://www.kegg.jp/kegg/kegg1.html).

The methods for MS are described in the Supplementary methods.

## Supplementary Information

Supplementary Information 1.

Supplementary Information 2.

Supplementary Information 3.
