# Supplementary material for: MYCL promotes iPSC-like colony formation via MYC Box 0 and 2 domains
Source: Sci Rep. 2021 Dec 20;11:24254. doi: 10.1038/s41598-021-03260-5 (PMC8688507; doi:10.1038/s41598-021-03260-5)
Supplement: Supplementary file 1 — Supplementary Information 1. [file 41598_2021_3260_MOESM1_ESM.pdf]

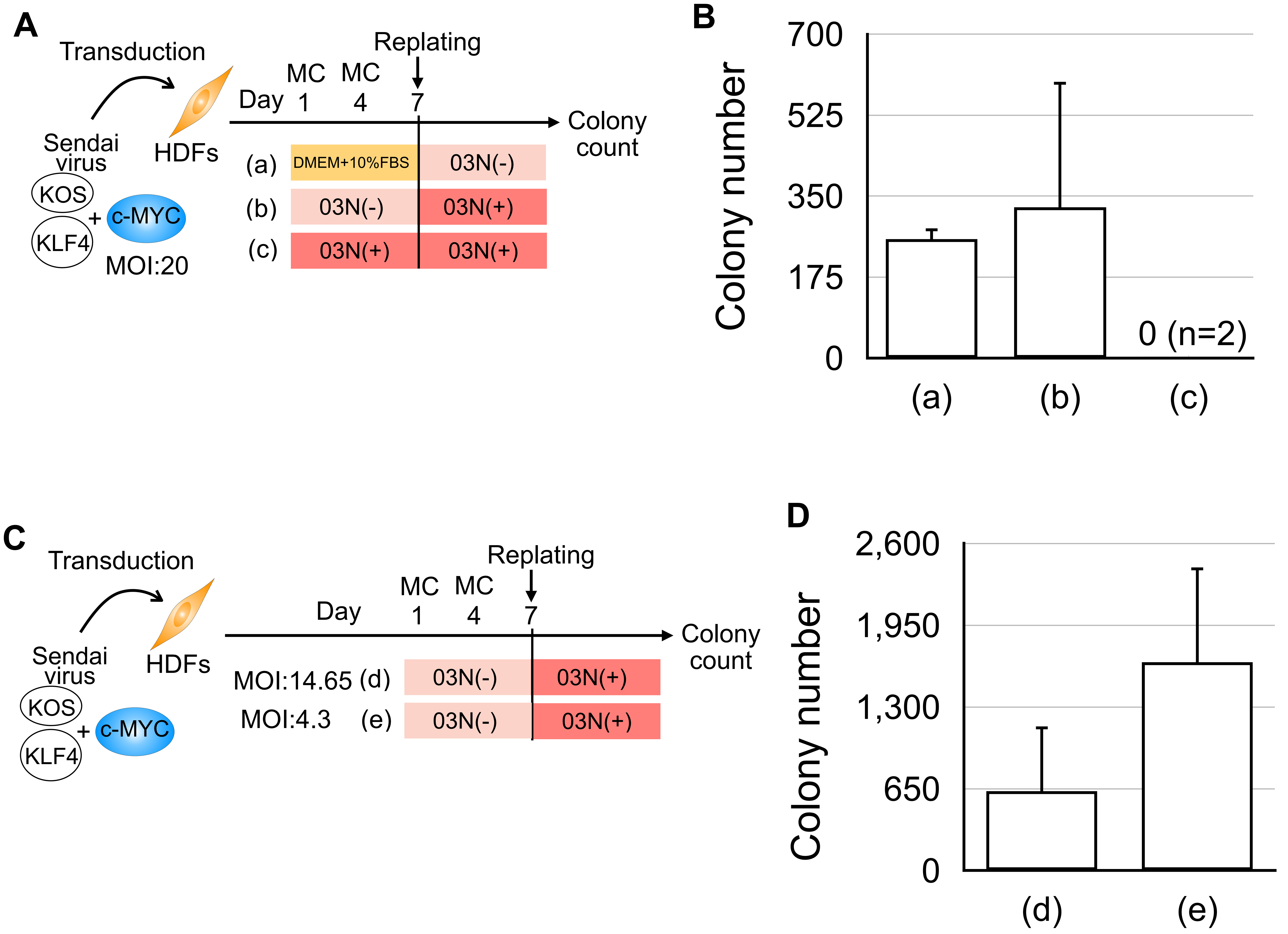
A
Transduction
Sendai virus
KOS
KLF4
+ c-MYC
MOI:20
HDFs
Replating
Day
MC 1
MC 4
7
Colony count
(a) DMEM+10%FBS 03N(-)
(b) 03N(-) 03N(+)
(c) 03N(+) 03N(+)
B
Colony number
700
525
350
175
0
0 (n=2)
(a)
(b)
(c)
C
Transduction
Sendai virus
KOS
KLF4
+ c-MYC
HDFs
Replating
Day
MC 1
MC 4
7
Colony count
MOI:14.65 (d) 03N(-) 03N(+)
MOI:4.3 (e) 03N(-) 03N(+)
D
Colony number
2,600
1,950
1,300
650
0
(d)
(e)

A

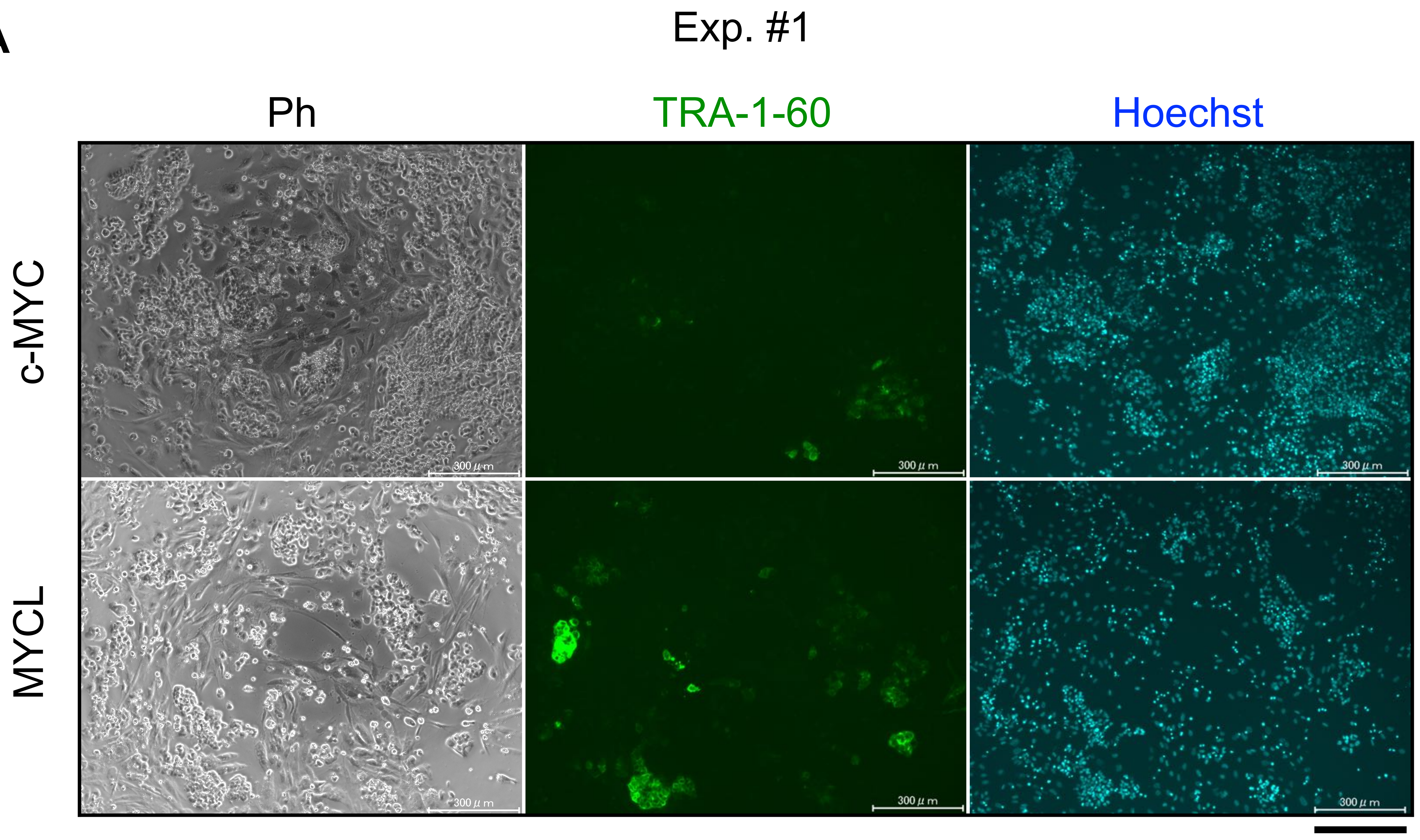
Exp. #1
Ph
TRA-1-60
Hoechst
c-MYC
MYCL

B

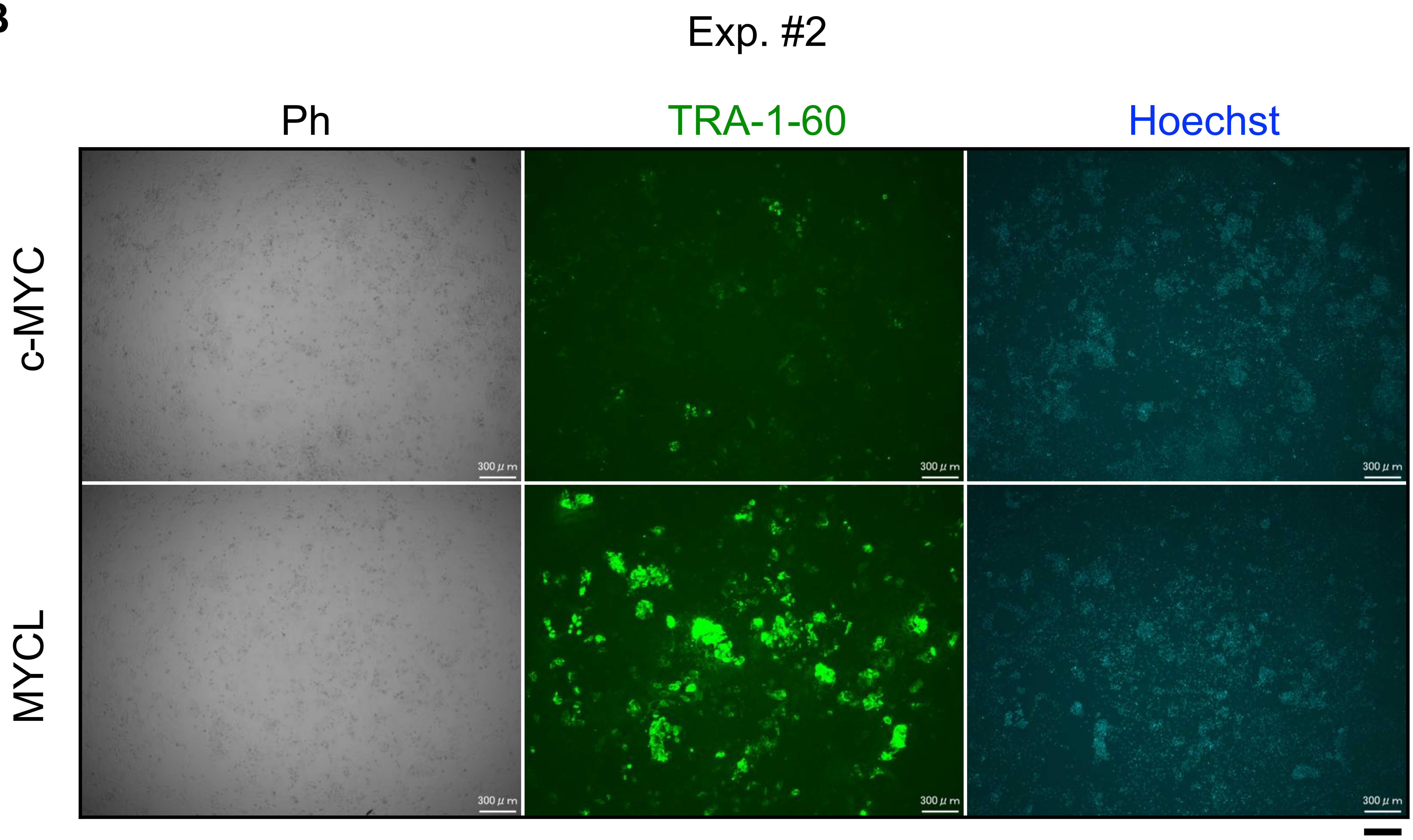
Exp. #2
Ph
TRA-1-60
Hoechst
c-MYC
MYCL

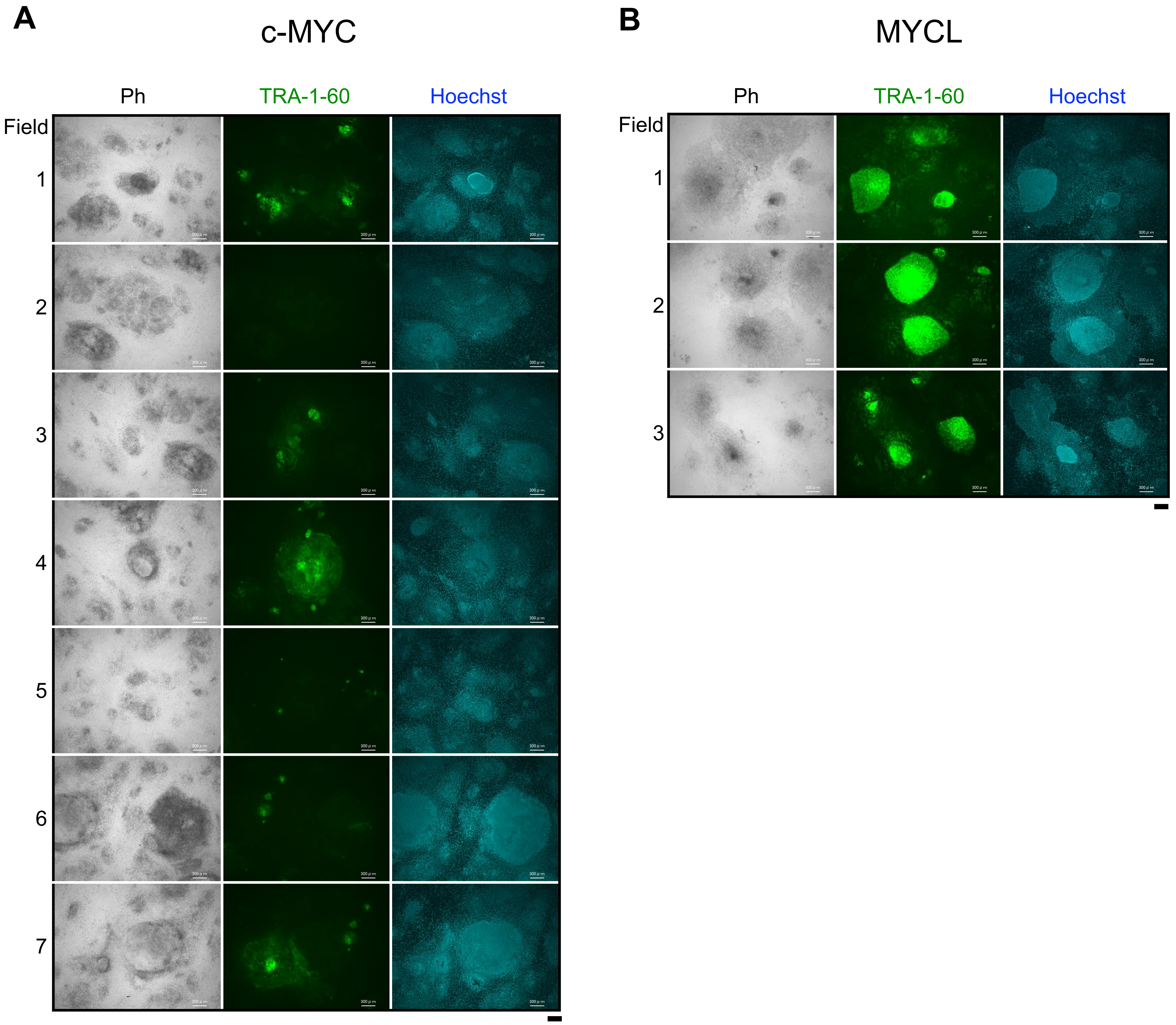
A
c-MYC
Ph
TRA-1-60
Hoechst
Field
1
2
3
4
5
6
7
B
MYCL
Ph
TRA-1-60
Hoechst
Field
1
2
3

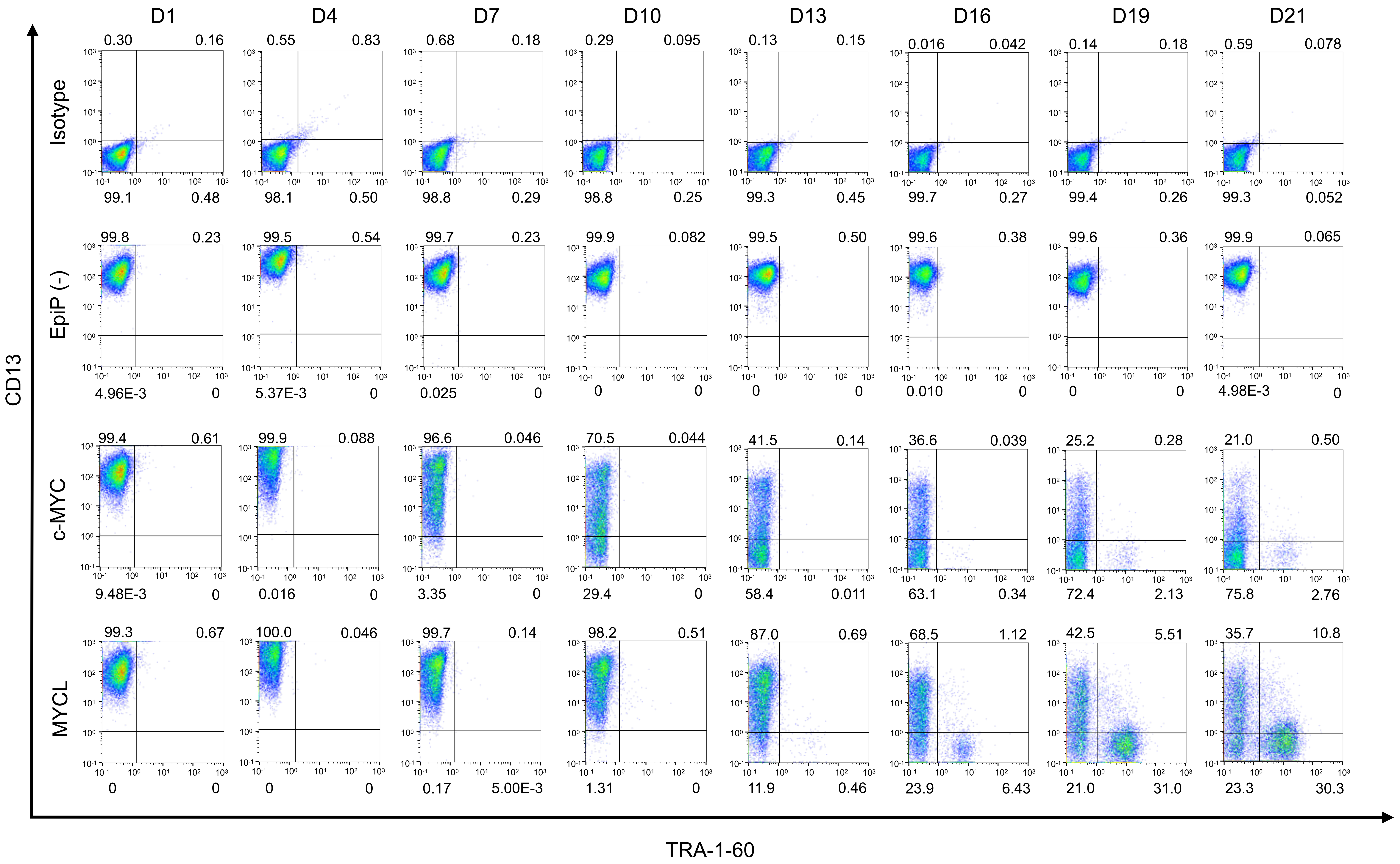
D1
D4
D7
D10
D13
D16
D19
D21
Isotype
EpiP (-)
c-MYC
MYCL
CD13
TRA-1-60

A

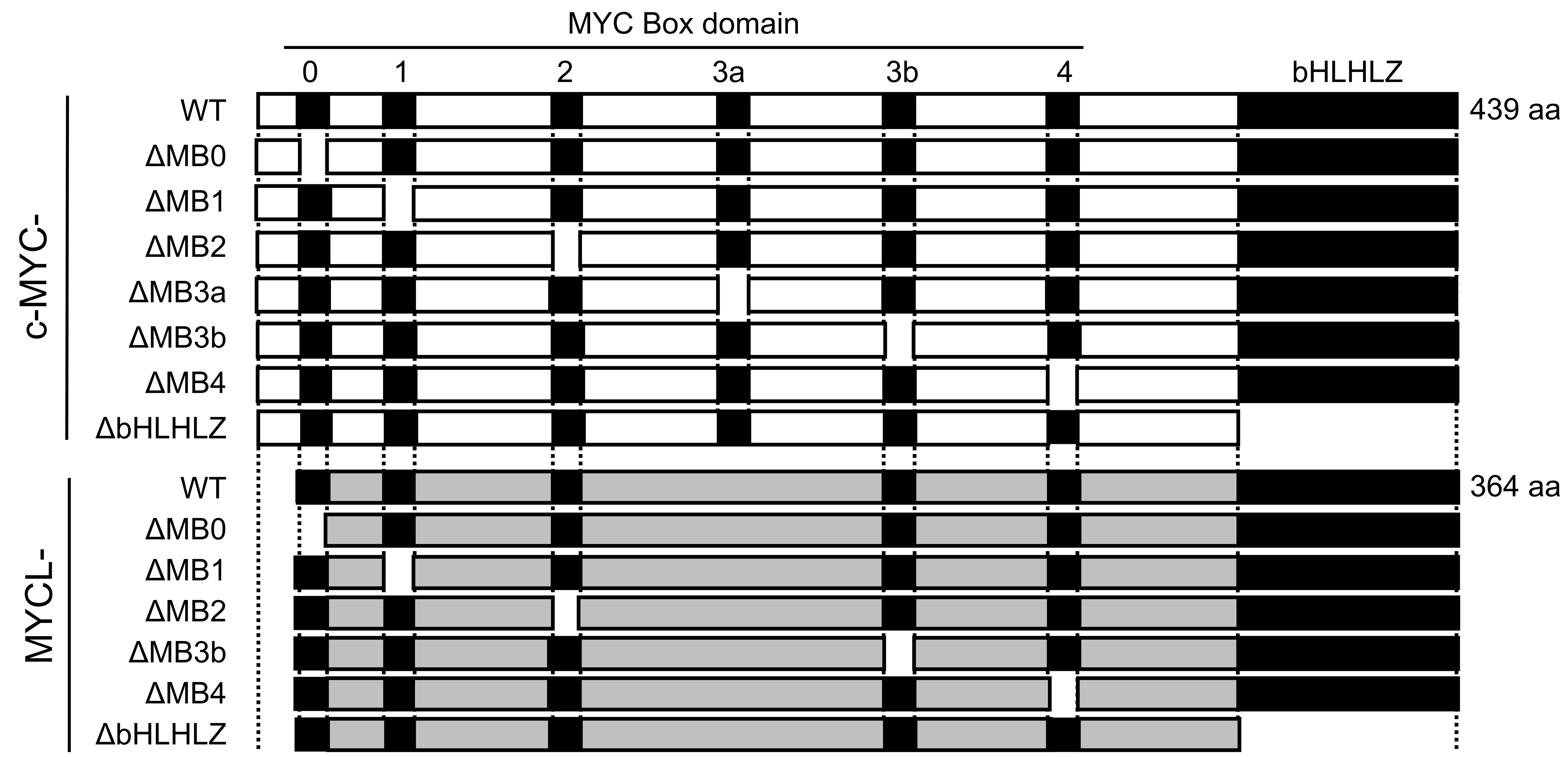

B

```
                                    MB0                                         MB1
           1                                                                                                                   100
    c-MYC  MPLNVSFTNR NYDLDYDSVQ PYFY-CDEEE NFYQQQQQSE LQPPAPSEDI WKKFELLPTP PLSPSRRSGL CSPSYVAVTP FSLRGDNDGG GGSFSTADQL
     MYCL                 MDYDSYQ HYFYDYDCGE DFYRST---- ----APSEDI WKKFELVPSP PTSPPWGLG- --PGAGDPAP GIGPPEPWPG G---CTGDEA
Consensus  .......... ...$DYDSyQ hYFY.cDceE #FYrqq.... ....APSEDI WKKFELlPsP PlSPprrlG. ..PgagapaP gigrg#ndgG G...cTaD#a

                                                 MB2                                                          MB3a
           101                                                                                                                 200
    c-MYC  EMVTELLGGD MVNQSFICDP DDETFIKNII IQDCMWSGFS AAAKLVSEKL ASYQAARKDS GSPNPARGHS VCSTSSLYLQ DLSAAASECI DPSVVFPYPL
     MYCL  ESRGHSKGWG RNYAS----- --------II RRDCMWSGFS ARERL-ERAV SDRLAPGAPR GNPPKASAAP DCTPSL---- -------EAG NPAPAAPCPL
Consensus  EmrgellGgd rnnaS..... ........II rrDCMWSGFS AaarL.eral adrqAaradr GnPnkAraap dCspSl.... .......Eag #PapaaPcPL
                                                 *

                                                                                  MB3b
           201                                                                                                                 300
    c-MYC  NDSSSPKSCA SQDSSAFSPS SDSLLSSTES SPQGSPEPLV LHEETPPTTS SDSEEEQEDE EEIDVVSVEK RQAPGKRSES GSPSAGGHSK PPHSPLVLKR
     MYCL  GEPKT-QACS GSESPSDSEN EEIDVVTVEK R-----QSLG IRKPVTITVR ADPLDPCMKH FHISIHQQQH NYAARFPPES CSQEEASERG PQEEVL----
Consensus  n#pks.qaCa gq#SpadSen e#idlsstEk r.....#pLg ireetpiTtr aDpe#ecede eeId!hqq#h rqAarkrpES cSqeaagerg PqeepL....

                 MB4
           301                                                                                                                 400
    c-MYC  CHVSTHQHNY AAPPSTRKDY PAAKRVKLDS VRVLRQISNN RKCTSPRSSD TEENVKRRTH NVLERQRRNE LKRSFFALRD QIPELENNEK APKVVILKKA
     MYCL  ------ERDA AGEKEDEEDE EIVSPPPVES EAA------Q SCHPKPVSSD TEDVTKRKNH NFLERKRRND LRSRFLALRD QVPTLASCSK APKVVILSKA
Consensus  ......#r#a AaekedreDe eaakrpkl#S eaa......# rccpkPrSSD TE#ntKRrnH NfLERqRRN# LrrrFlALRD Q!PeLanneK APKVVILkKA
                                                                      Basic Region          Helix1          Loop       Helix2

           401                                     440
    c-MYC  TAYILSVQAE EQKLISEEDL LRKRREQLKH KLEQLRNSCA
     MYCL  LEYLQALVGA EKRMATEKRQ LRCRQQQLQK RIAYLTGY
Consensus  laYiqalqaa Eqr$asEerq LRcRr#QLqh riaqLrns..
             Helix2              Zipper
```

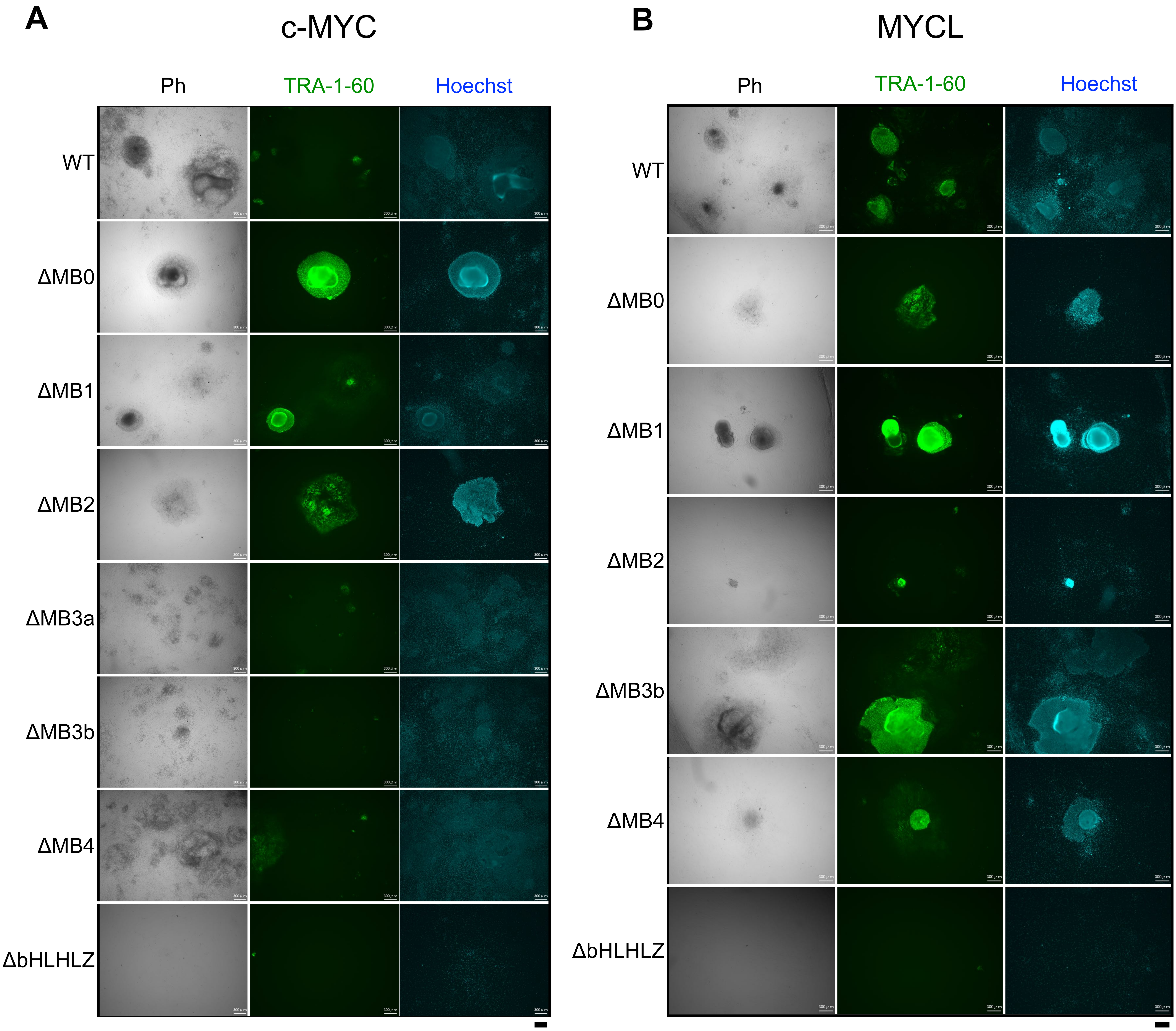
A
c-MYC
Ph
TRA-1-60
Hoechst
WT
ΔMB0
ΔMB1
ΔMB2
ΔMB3a
ΔMB3b
ΔMB4
ΔbHLHLZ
B
MYCL
Ph
TRA-1-60
Hoechst
WT
ΔMB0
ΔMB1
ΔMB2
ΔMB3b
ΔMB4
ΔbHLHLZ

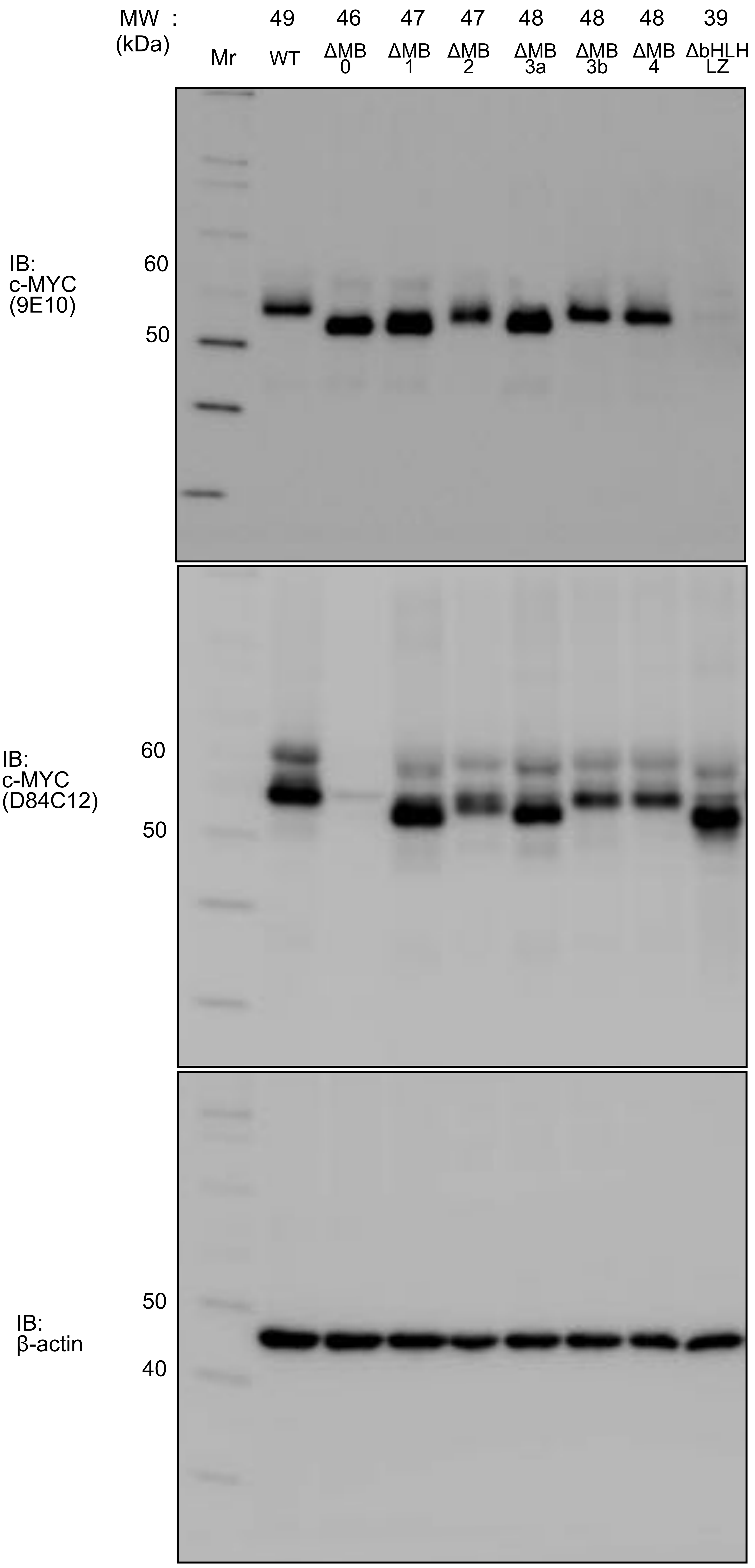
MW :
(kDa)
Mr
49
WT
46
ΔMB 0
47
ΔMB 1
47
ΔMB 2
48
ΔMB 3a
48
ΔMB 3b
48
ΔMB 4
39
ΔbHLH LZ
IB:
c-MYC
(9E10)
60
50
IB:
c-MYC
(D84C12)
60
50
IB:
β-actin
50
40

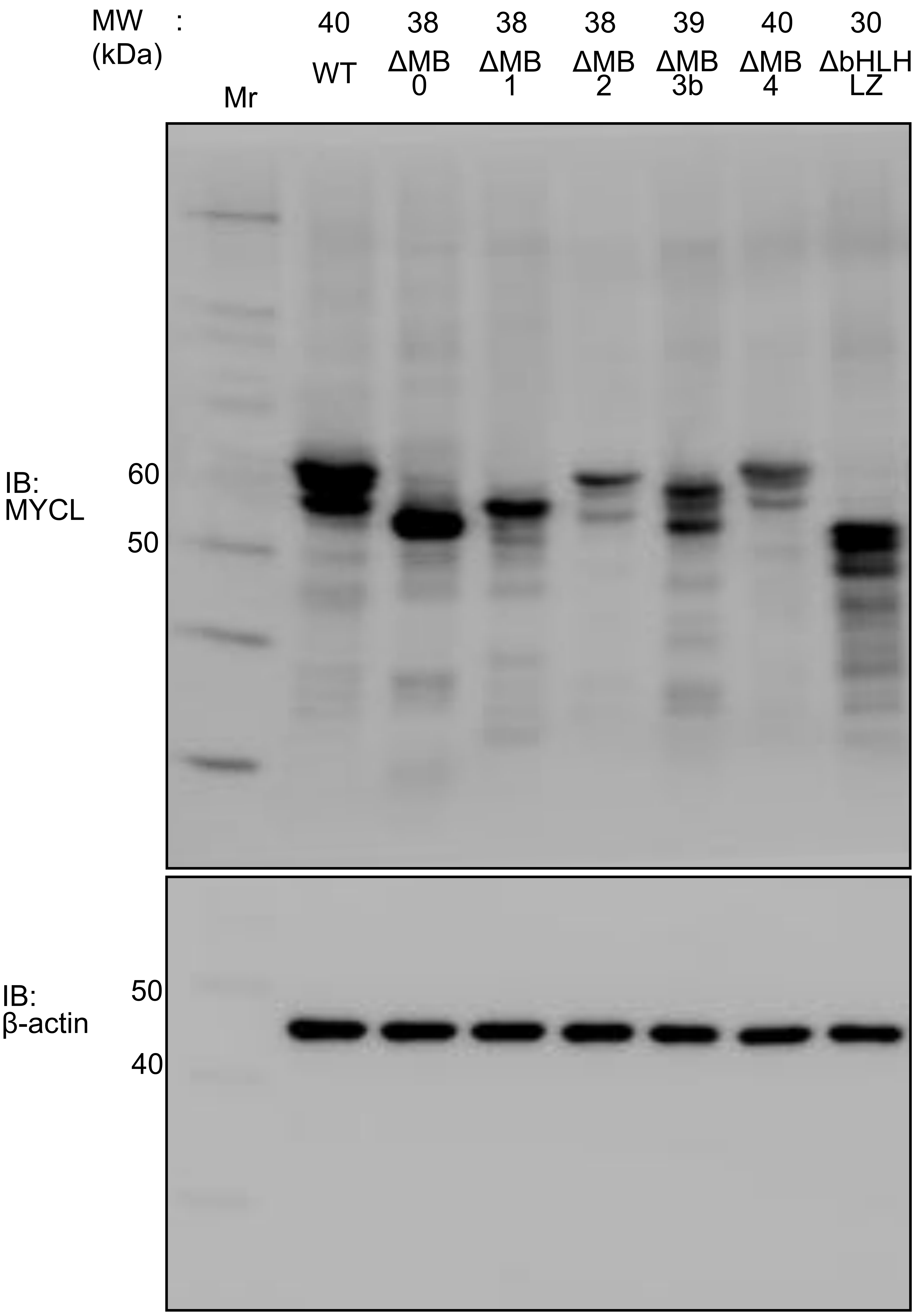
MW (kDa) :
40 WT
38 ΔMB 0
38 ΔMB 1
38 ΔMB 2
39 ΔMB 3b
40 ΔMB 4
30 ΔbHLH LZ
Mr
IB: MYCL
60
50
IB: β-actin
50
40

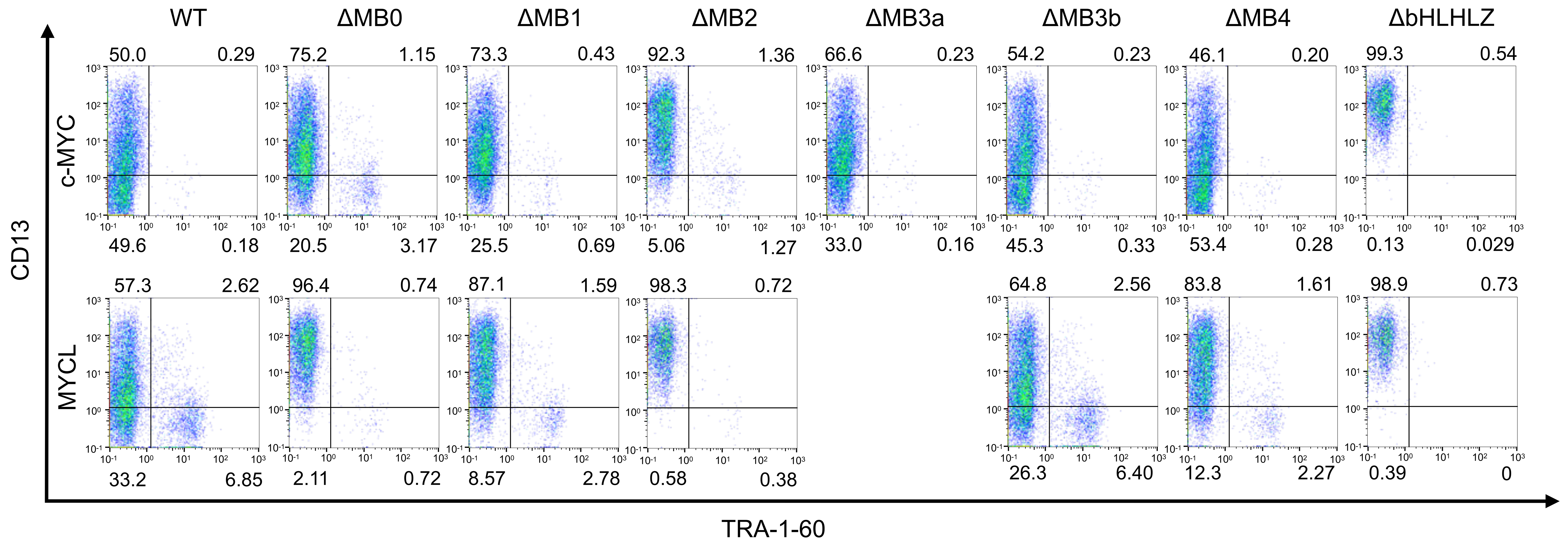
WT
ΔMB0
ΔMB1
ΔMB2
ΔMB3a
ΔMB3b
ΔMB4
ΔbHLHLZ
CD13
c-MYC
MYCL
TRA-1-60
50.0 0.29 49.6 0.18
75.2 1.15 20.5 3.17
73.3 0.43 25.5 0.69
92.3 1.36 5.06 1.27
66.6 0.23 33.0 0.16
54.2 0.23 45.3 0.33
46.1 0.20 53.4 0.28
99.3 0.54 0.13 0.029
57.3 2.62 33.2 6.85
96.4 0.74 2.11 0.72
87.1 1.59 8.57 2.78
98.3 0.72 0.58 0.38
64.8 2.56 26.3 6.40
83.8 1.61 12.3 2.27
98.9 0.73 0.39 0

Akifuji *et al.*, Supplementary Figure S10

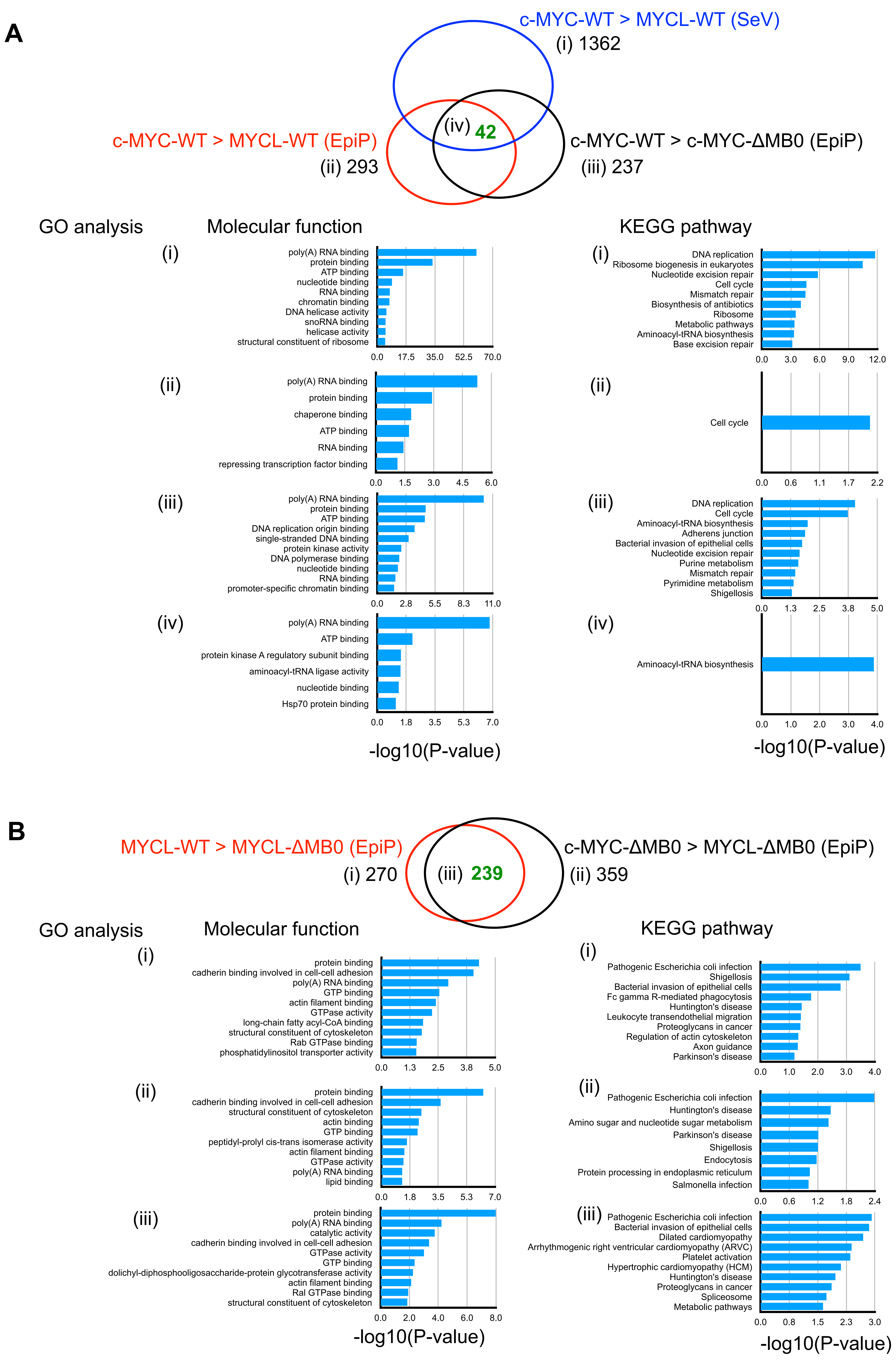

A Phosphorylated proteins in MYCL

FLNC	CD99	MAP1A
FILIP1L	TNS1	PITPNM1
PALLD	LZTS1	HSPB1
MYH11	CNOT2	KRT17
TACC1	SMG6	SAMD4B
NEXN	ASMTL	

GO analysis
Molecular function

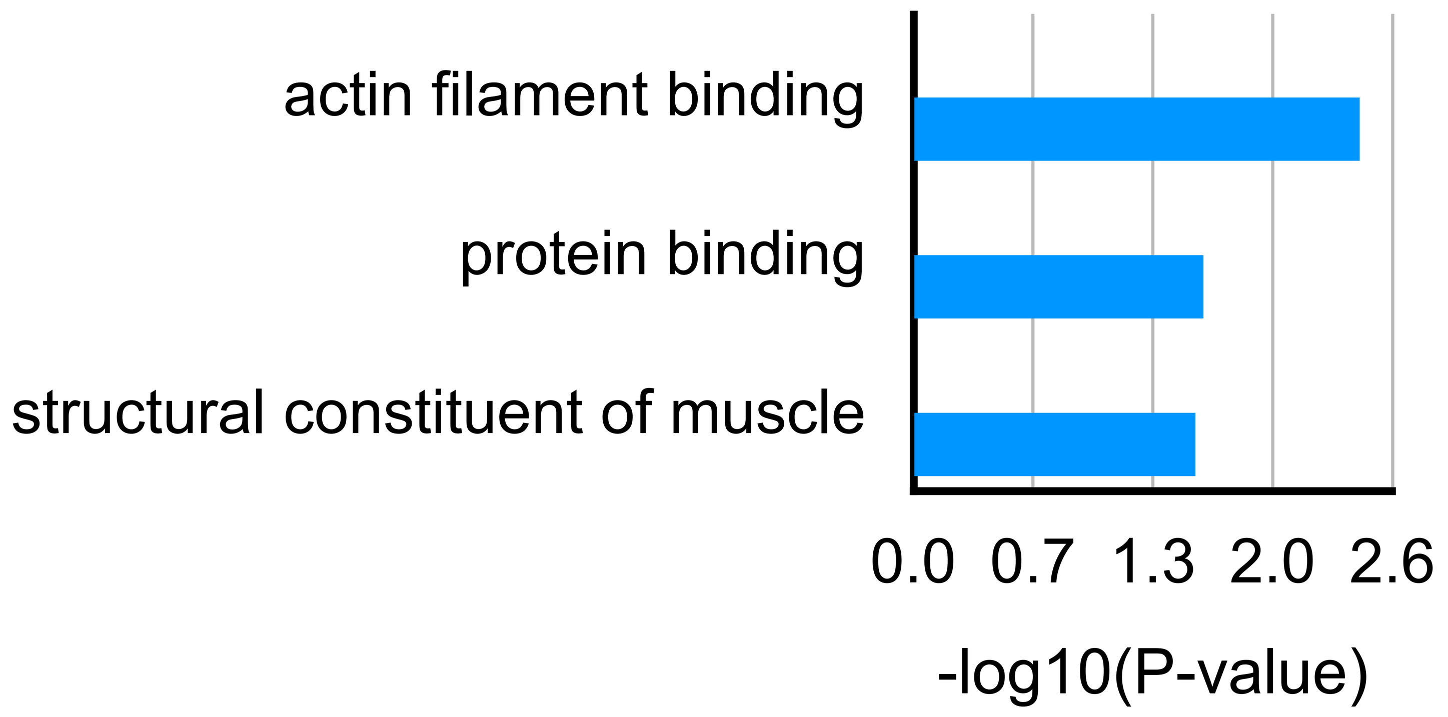

B Phosphorylated proteins in c-MYC

HNRNPA3	SRSF1	THRAP3	SSRP1	GTF3C1	UPF3B	PRR12	FAM208B	TNIK	ZBTB21	ZNF687	ZNF106
SALL2	TJP2	TJP1	NCK2	CHAMP1	POU2F1	ELMSAN1	TRIM24	SETDB1	KIZ	TLE3	GAL
SRRM2	TOP2A	SF3B1	L1TD1	GPS1	UTP14A	RANBP2	ALPL	SSB	MKI67	CGN	DSTN
LIN28A	U2SURP	HNRNPU	ENAH	SLC16A1	LARP1	GTF2I	DSG2	PAK4	MAP7	PARD3	NOLC1
TGS1	RAPGEF6	PHACTR2	LIG1	ZNF219	RBM10	KLF12	TTC28	RNF113A	ZMYND8	PKP4	CHAF1B
PAICS	TRIM71	ZNF423	ADGRL2	MASTL	SIPA1L1	CNKSR2	MDN1	RPL24	AGFG1	SLC9A3R1	UPF1
MDC 1	DNMT3B	PCDH1	ZRANB2	HSPA4	DOCK6	PDS5A	LSR	EPB41L5	STAU2	ATF7IP	MAP3K2
CERS4	TRIM28	TTF1	RBMX	MCM2	SRRM1	TRA2B	DPPA4	CLDN6	AATF	NASP	HMGA1
R3HDM2	ATRX	YBX1	ZNF638	TXLNA	NTHL1	KPNA2	SURF2	NUP35	SORT1	NOM1	ZNRF2
BCLAF1	ETV6	CXorf23	DDX54	BAG6	TCOF1	BCL7C	PSIP1	MTA1	ZNF358	UBAP2L	NPM1
FAM117B	POLDIP3	GIT1	SHC1	SNAPC5	LIG3	TMPO	CCNL1	LPAR1	NCL	BOP 1	ZNF608

GO analysis
Molecular function

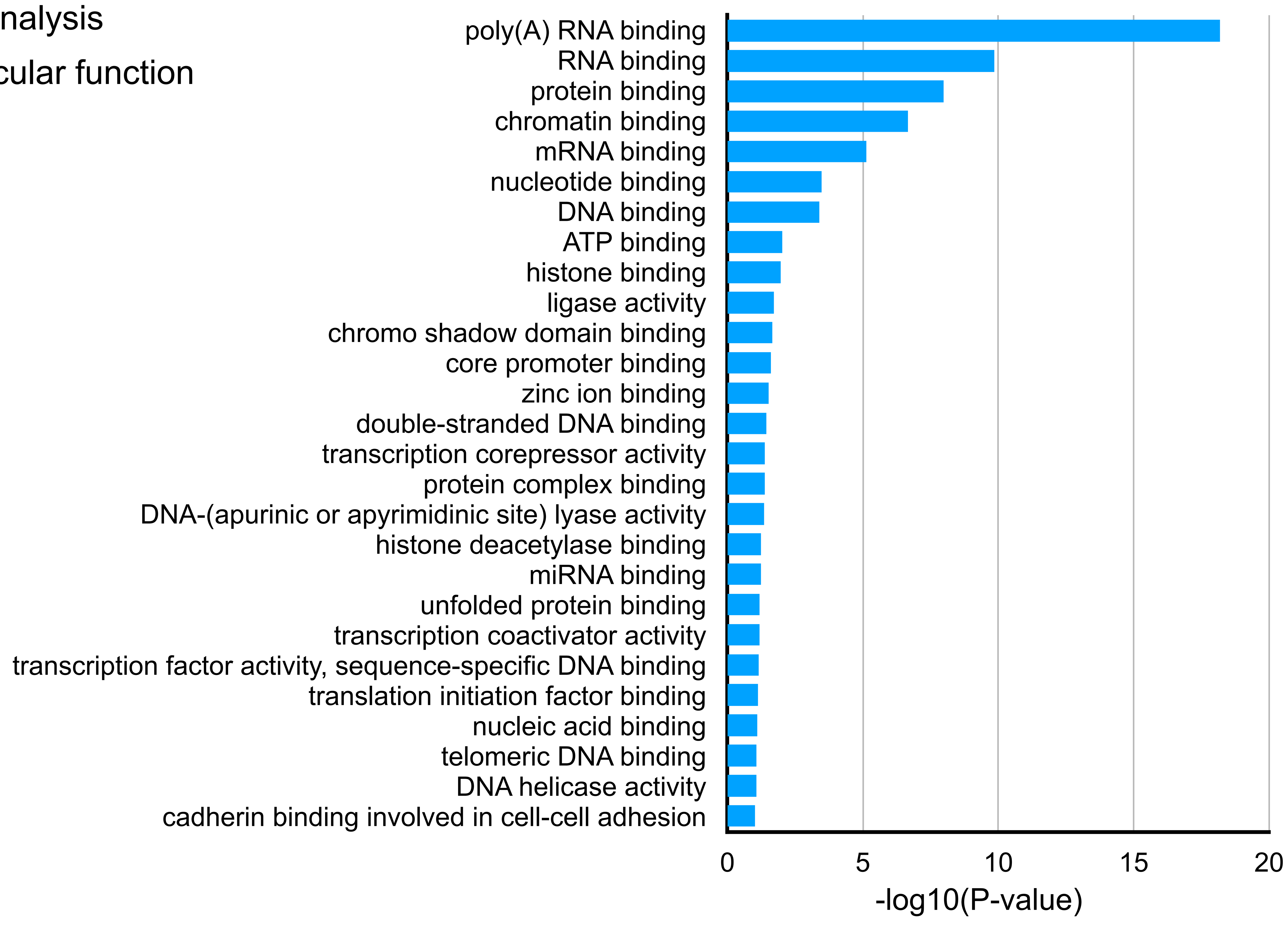

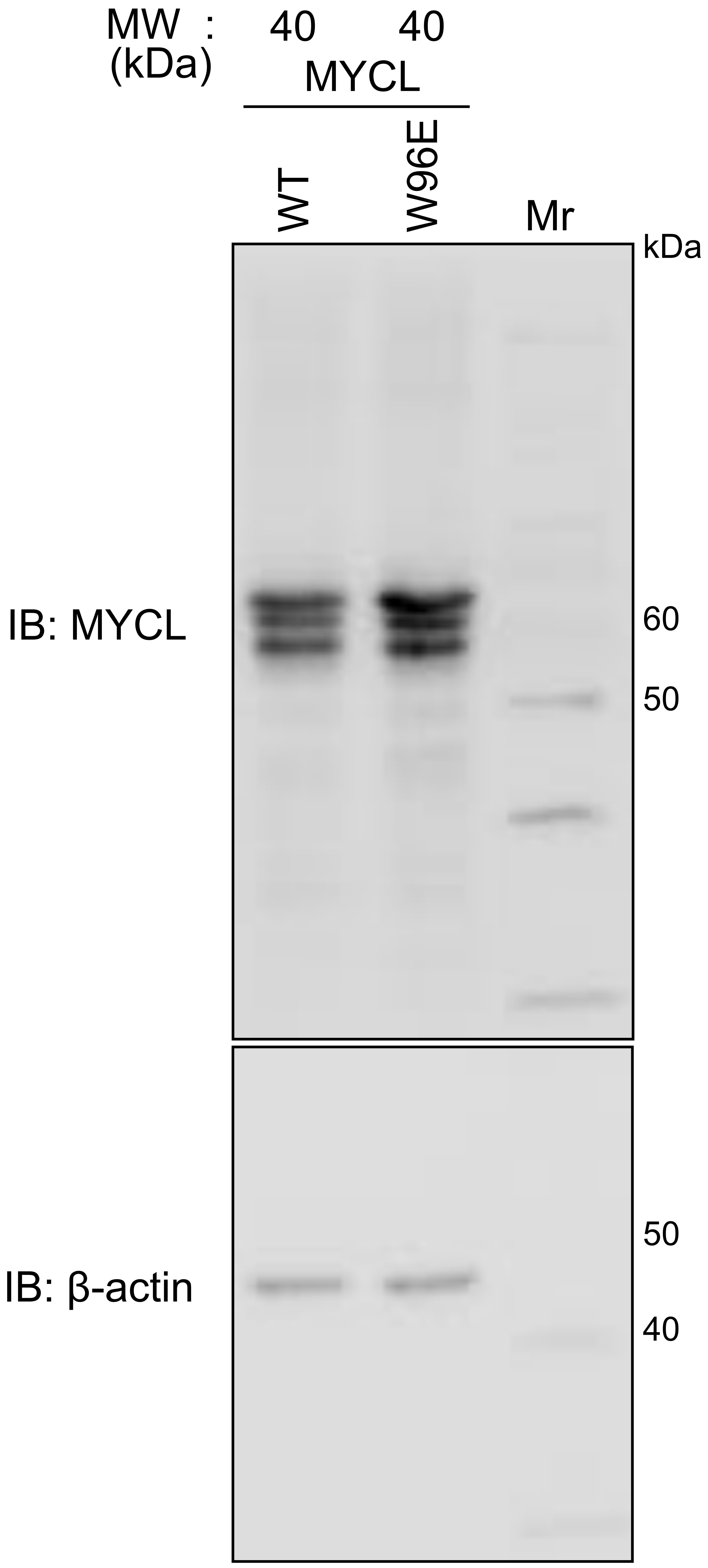

MW (kDa) : 40 40
MYCL
WT
W96E
Mr
kDa
IB: MYCL
60
50
IB: β-actin
50
40

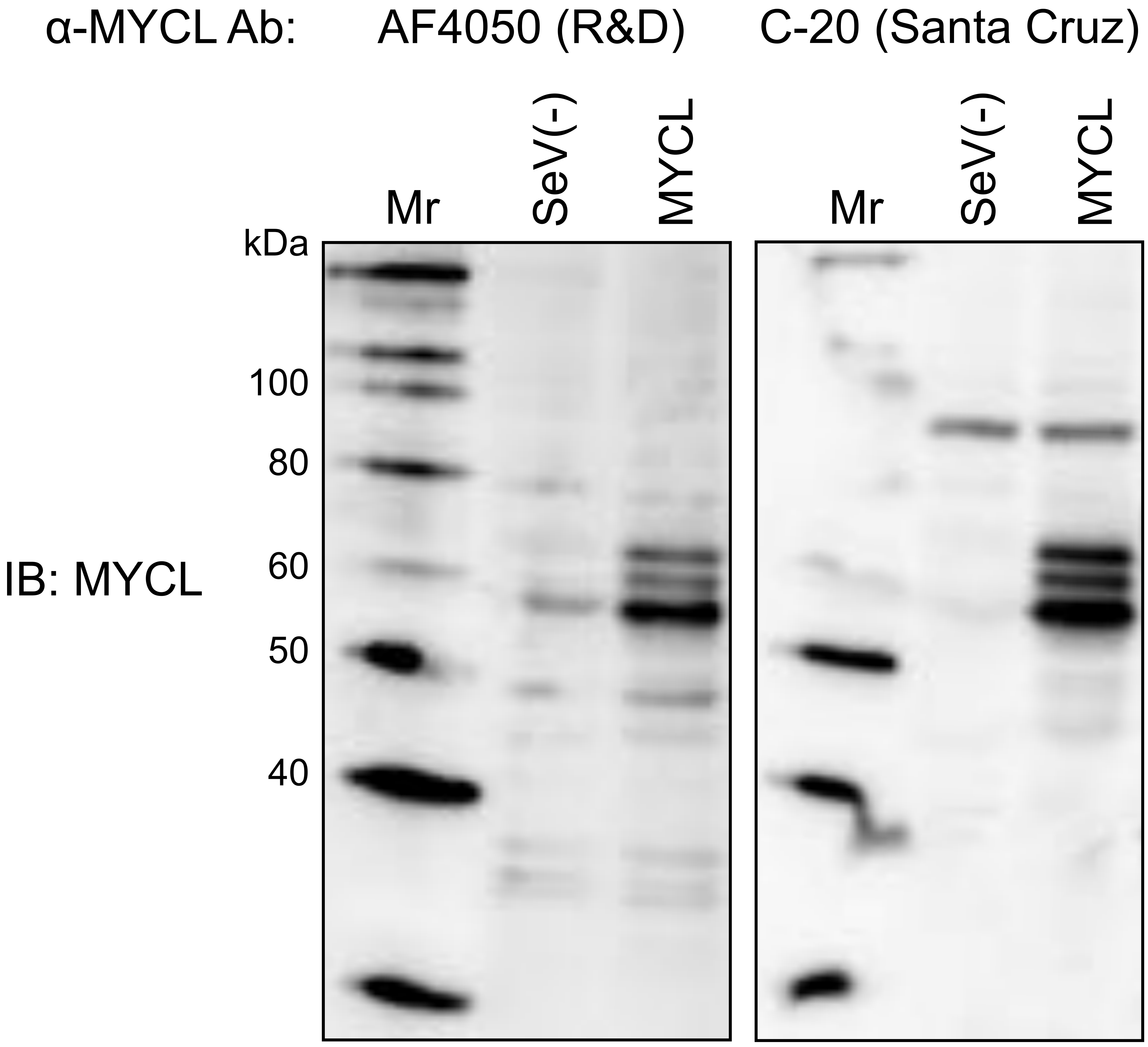
α-MYCL Ab:
AF4050 (R&D)
C-20 (Santa Cruz)
Mr
SeV(-)
MYCL
Mr
SeV(-)
MYCL
kDa
100
80
60
50
40
IB: MYCL

Originals for Supplementary Figure S7 (IB: c-MYC (9E10))

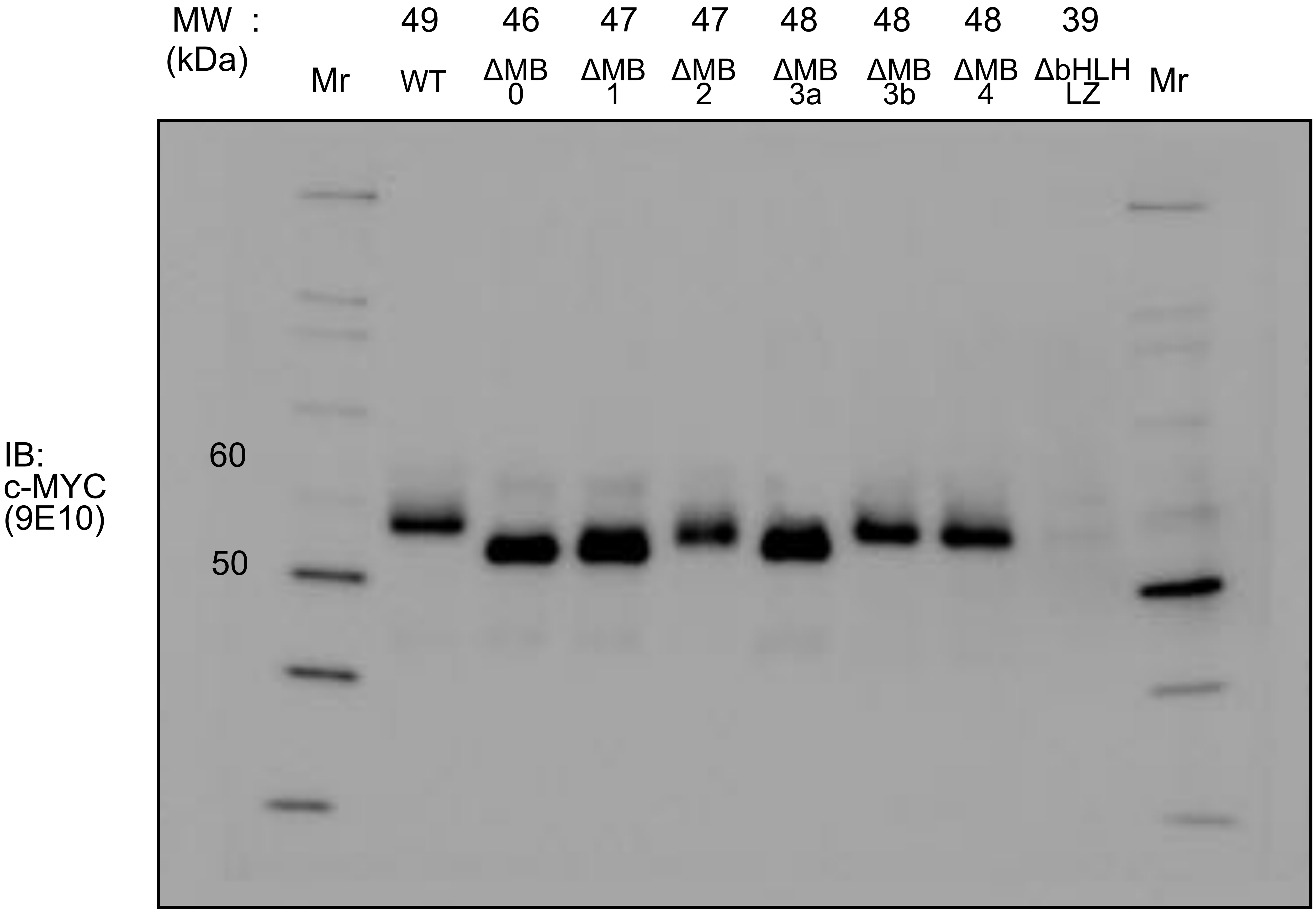

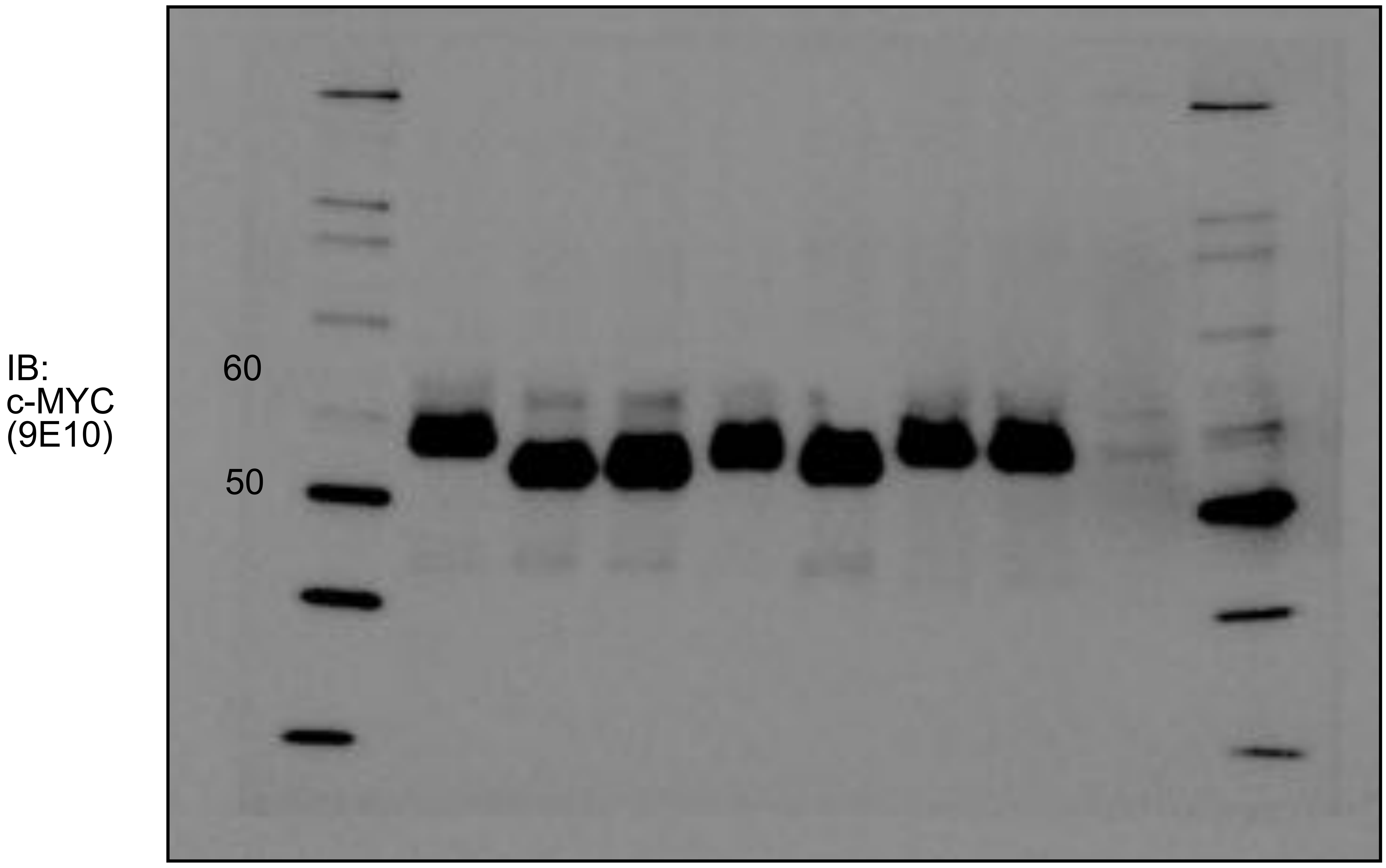

Contrast adjusted so that the edge of the membrane is visible.

Originals for Supplementary Figure S7 (IB: c-MYC (D84C12))

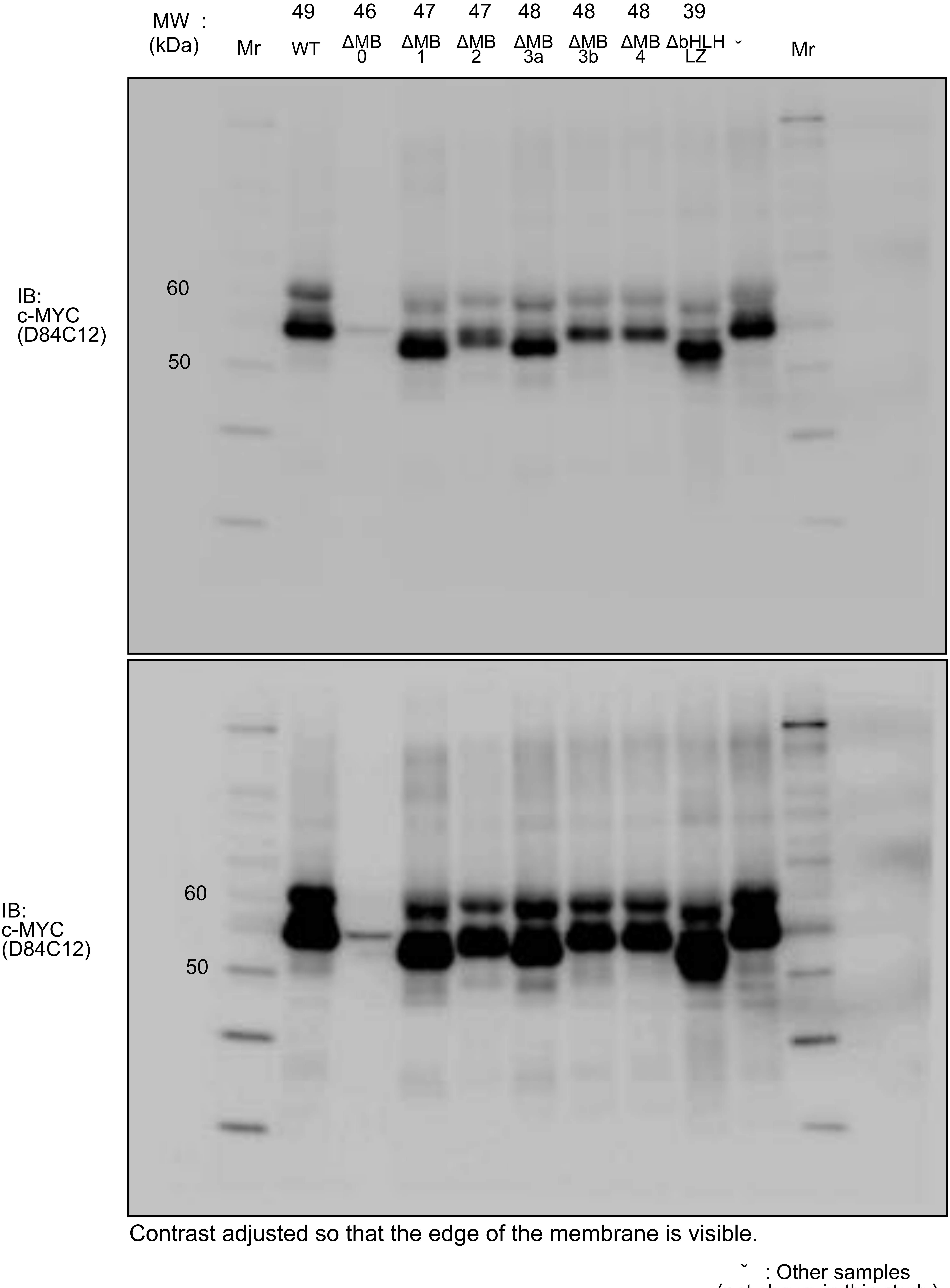

Contrast adjusted so that the edge of the membrane is visible.

ˇ : Other samples
(not shown in this study)

Originals for Supplementary Figure S7 (IB: β-actin)

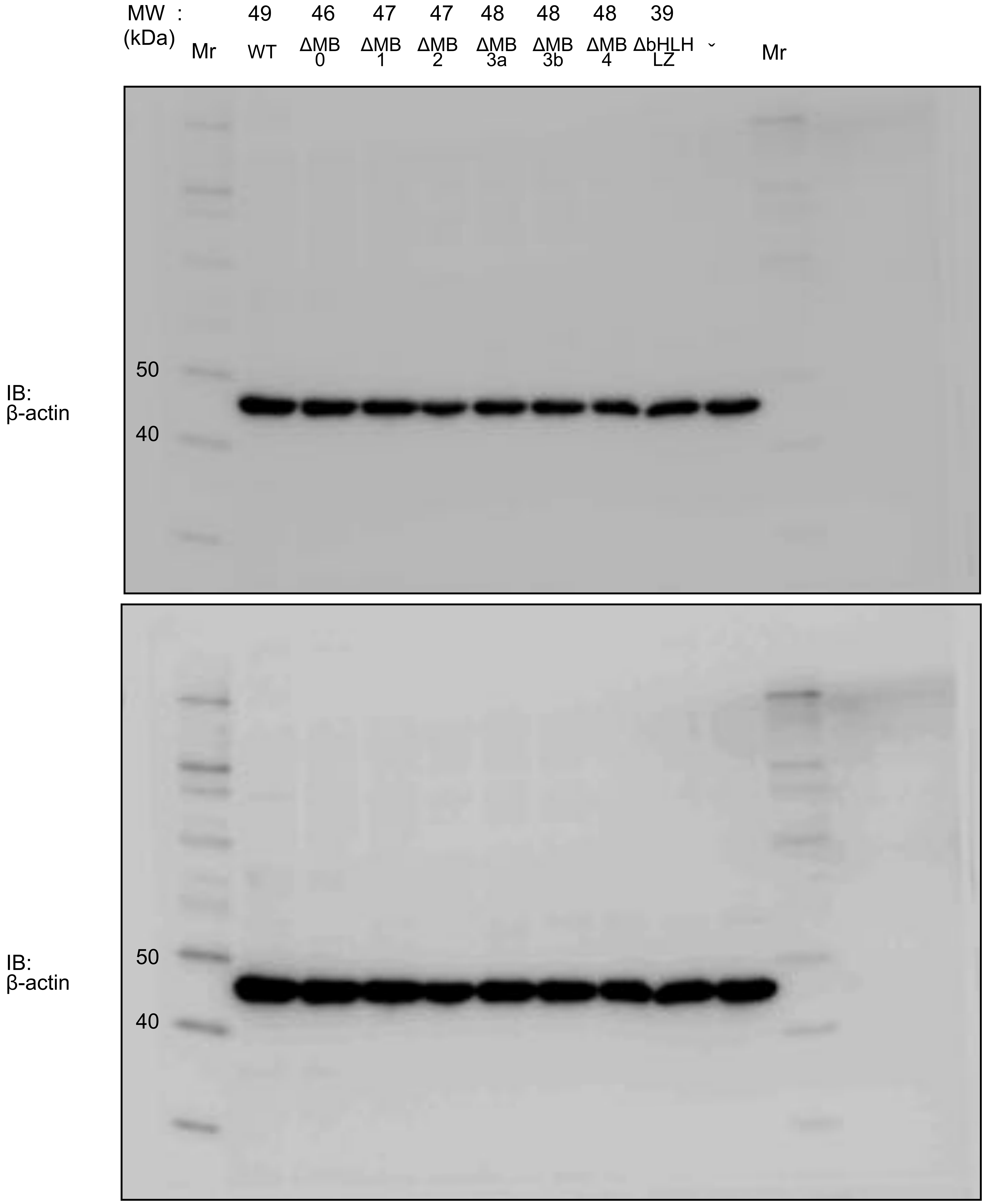

Contrast adjusted so that the edge of the membrane is visible.

˘ : Other samples
(not shown in this study)

Originals for Supplementary Figure S8 (IB: MYCL)

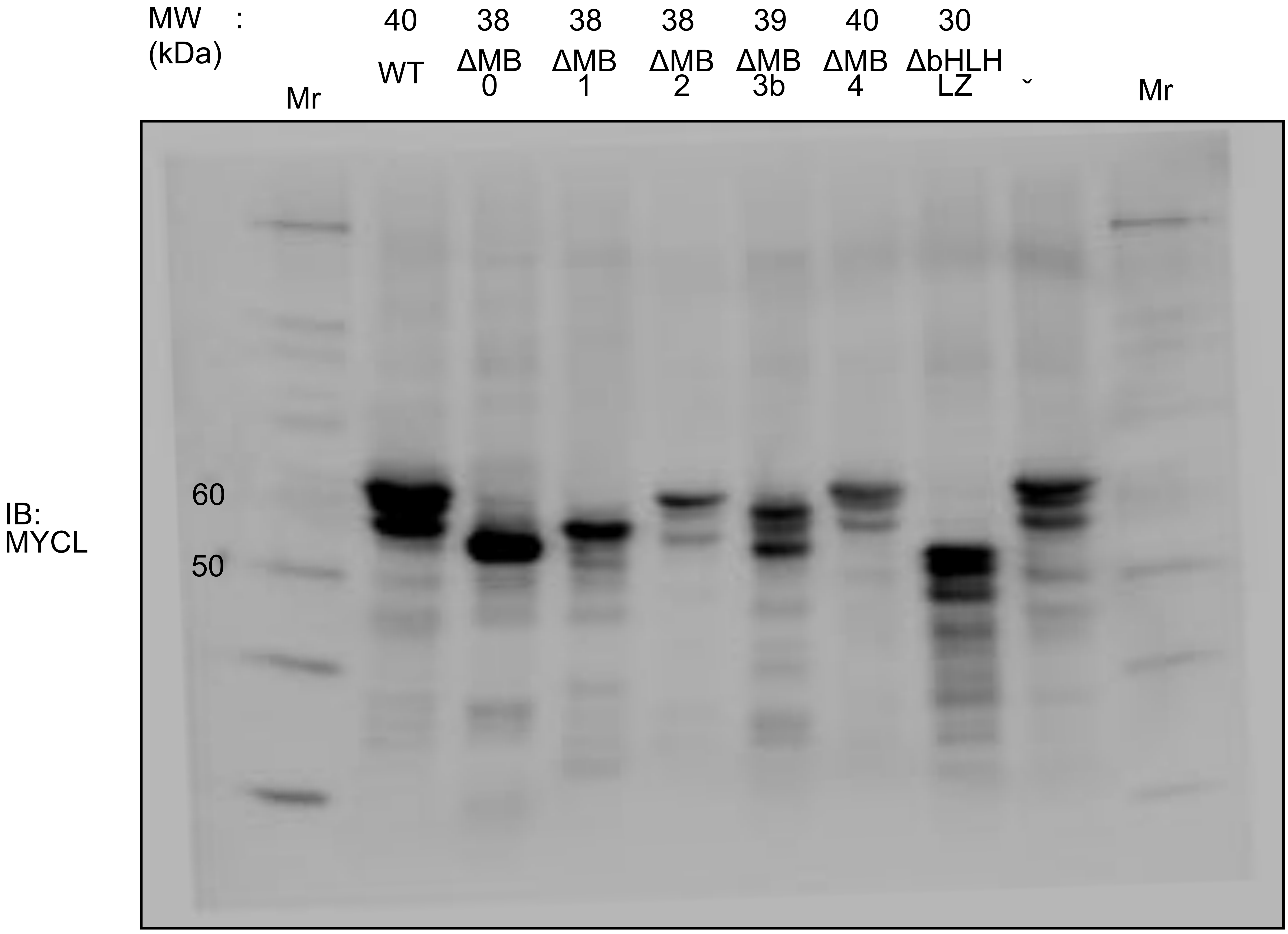

ˇ : Other samples (not shown in this study)

Originals for Supplementary Figure S8 (IB: β-actin)

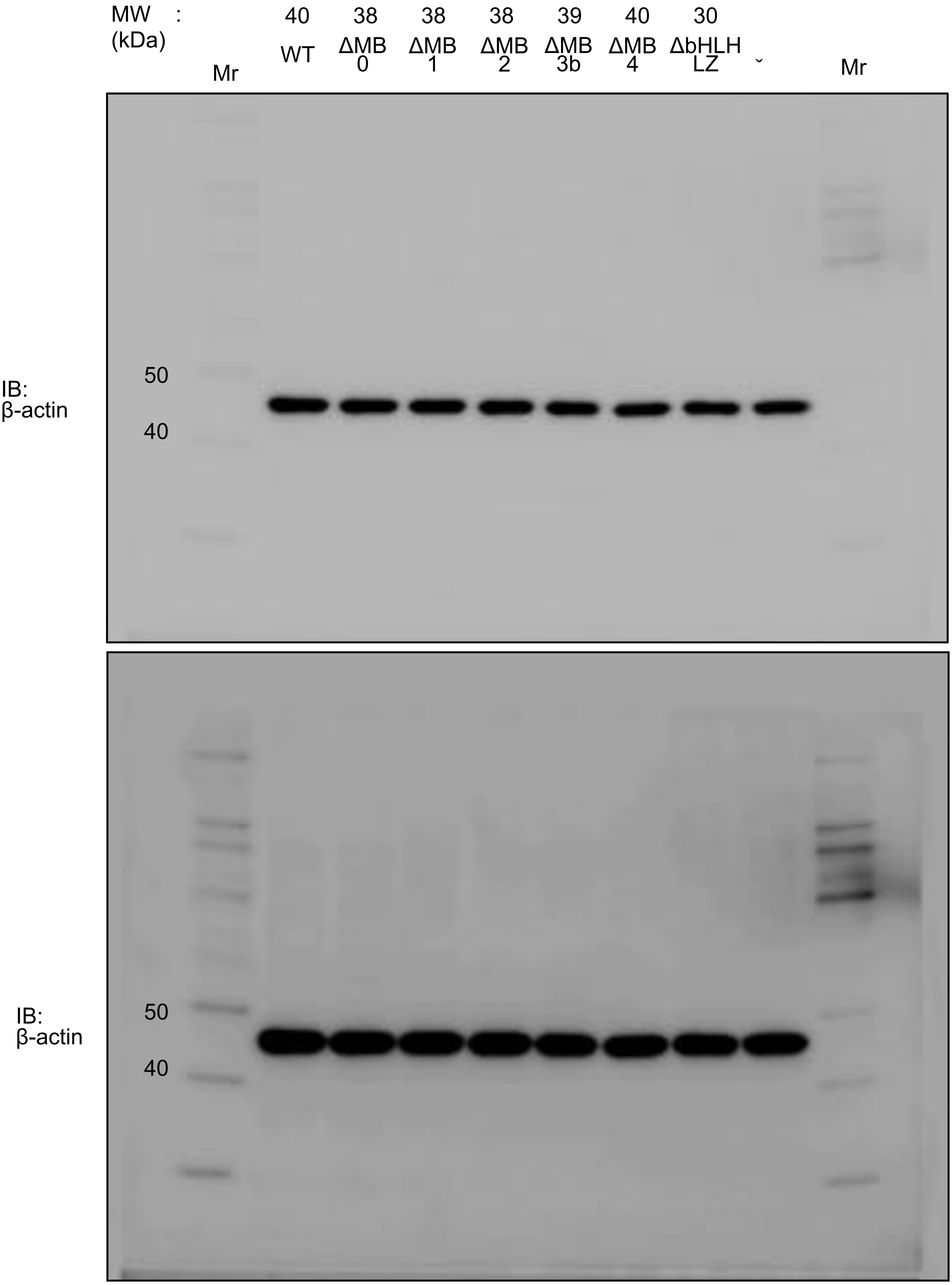

Contrast adjusted so that the edge of the membrane is visible.

ˇ : Other samples (not shown in this study)

Originals for Supplementary Figure S12 (IB: MYCL)

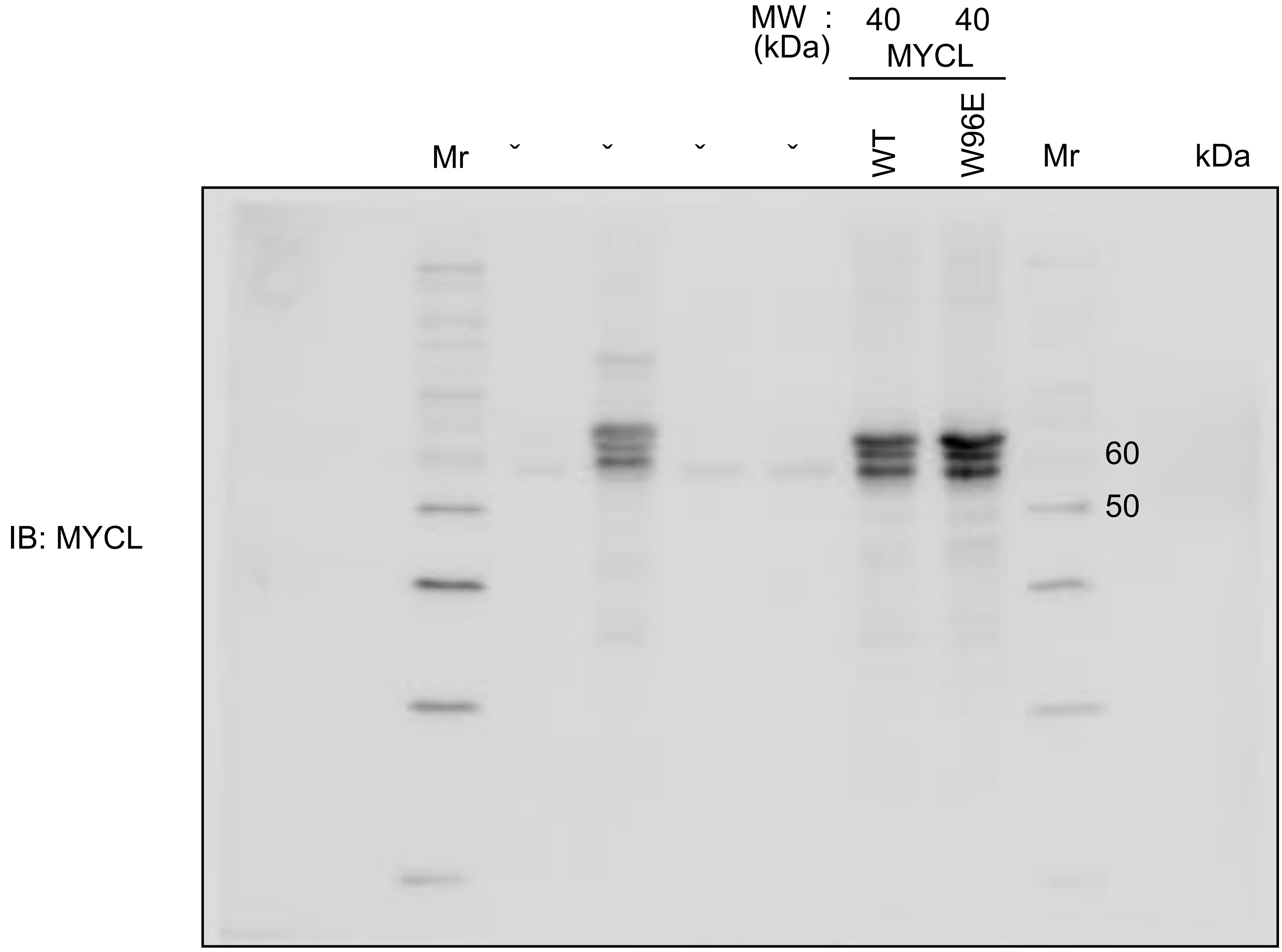

ˇ : Other samples (not shown in this study)

Originals for Supplementary Figure S12 (IB: β-actin)

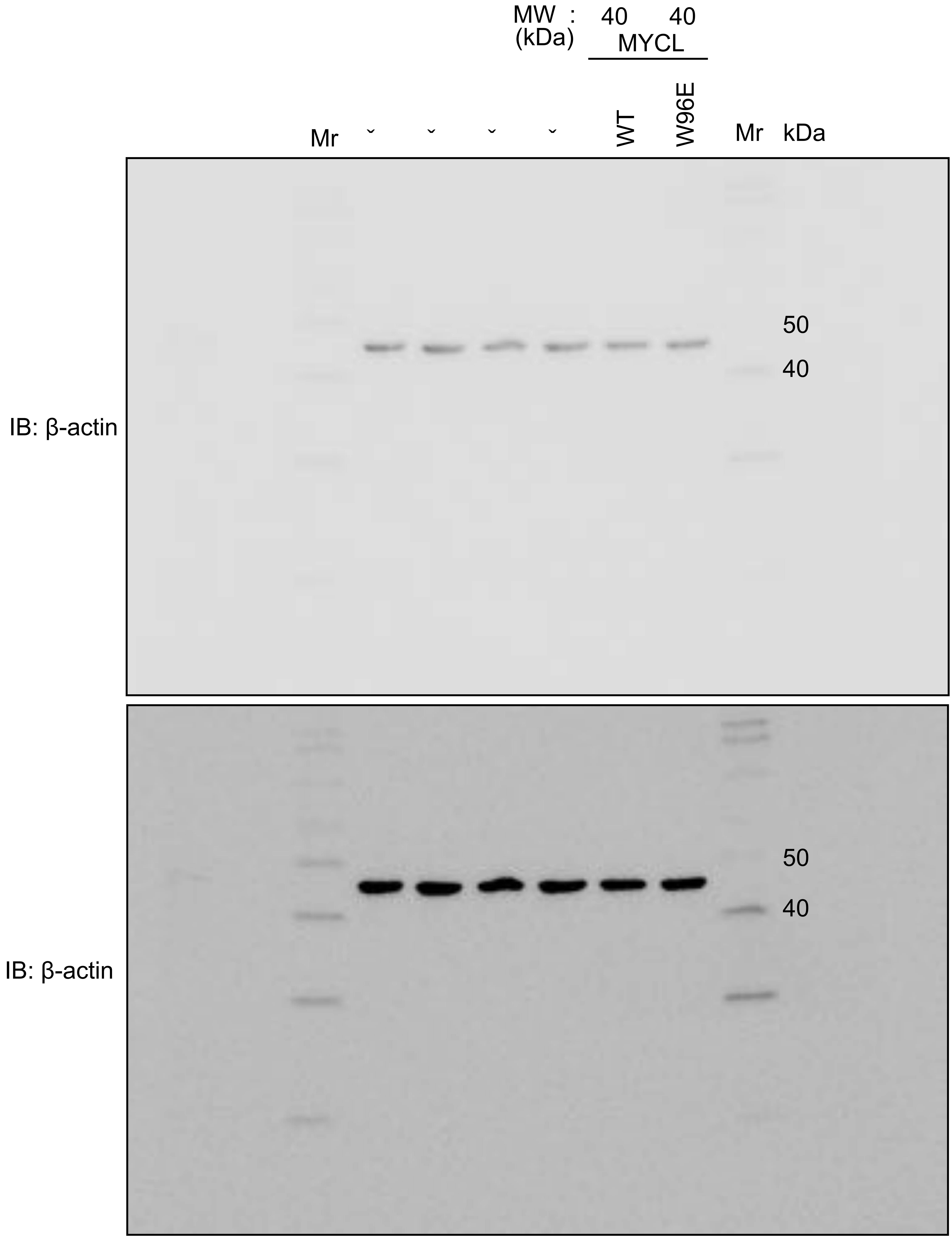

Contrast adjusted so that the edge of the membrane is visible.

ˇ : Other samples (not shown in this study)

Originals for Supplementary Figure S13 (IB: MYCL (AF4050))

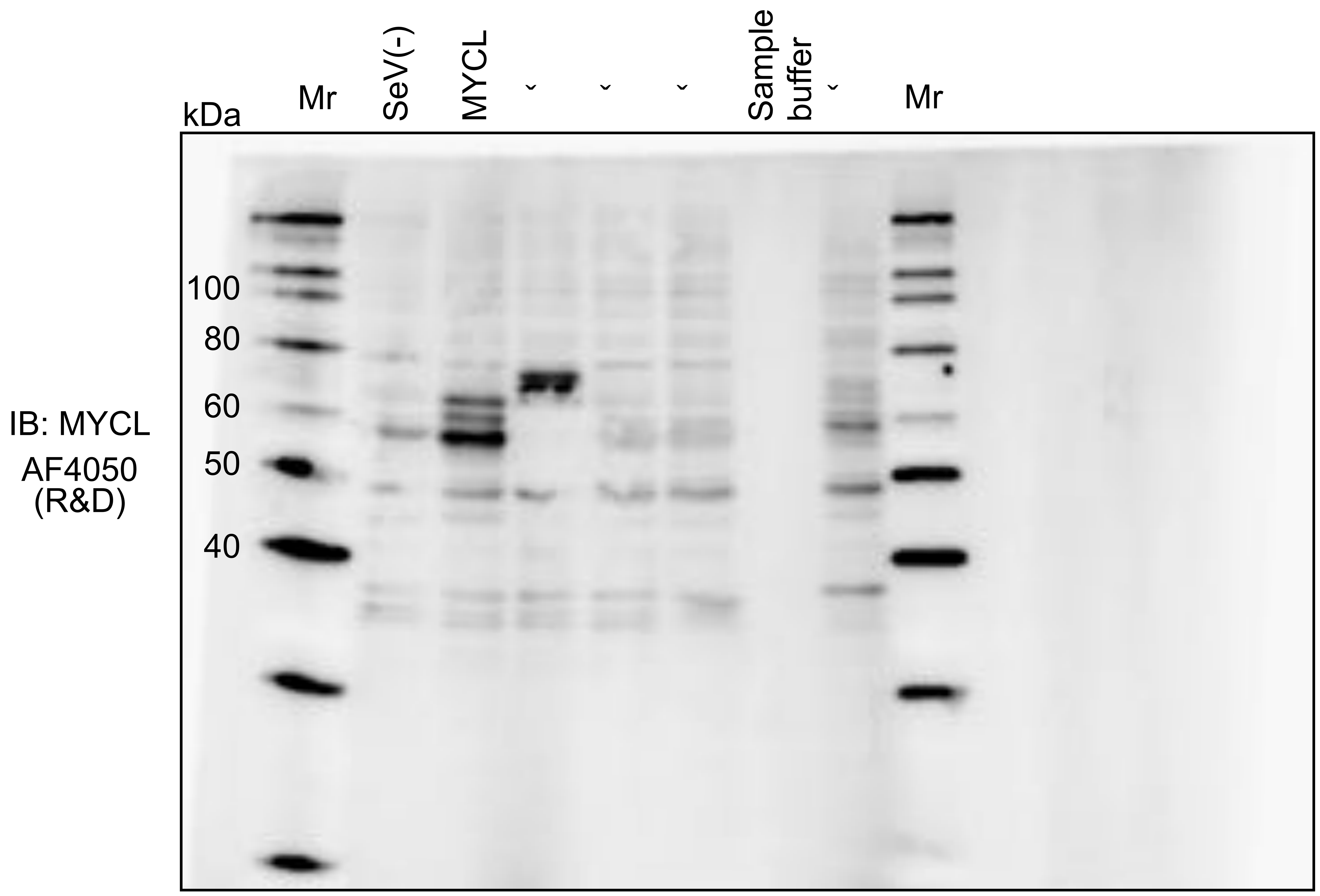

ˇ : Other samples
(not shown in this study)

Originals for Supplementary Figure S13 (IB: MYCL (C-20))

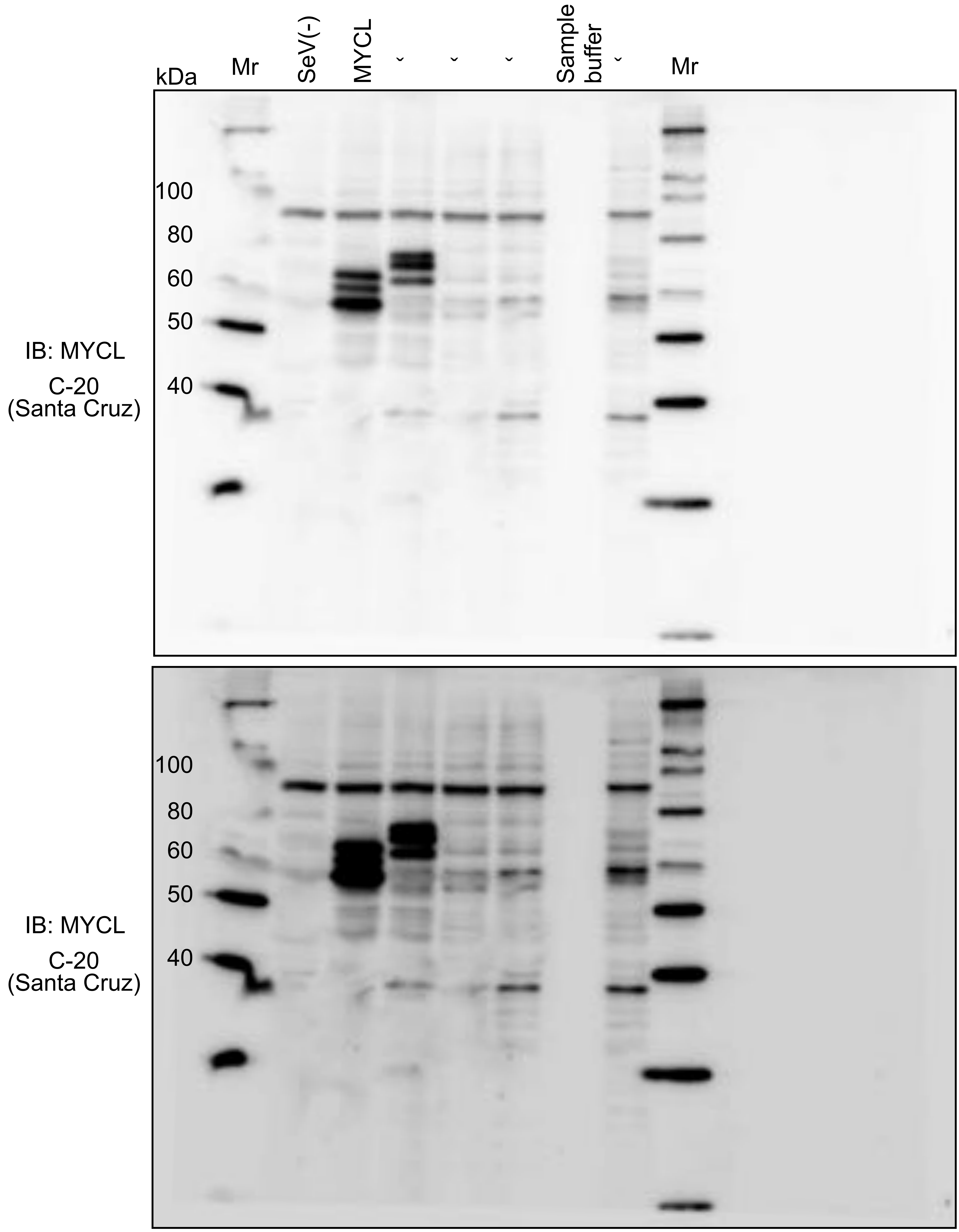

Contrast adjusted so that the edge of the membrane is visible.

ˇ : Other samples
(not shown in this study)
