# Supplementary material for: MYCL promotes iPSC-like colony formation via MYC Box 0 and 2 domains
Source: Sci Rep. 2021 Dec 20;11:24254. doi: 10.1038/s41598-021-03260-5 (PMC8688507; doi:10.1038/s41598-021-03260-5)
Supplement: Supplementary file 2 — Supplementary Information 2. [file 41598_2021_3260_MOESM2_ESM.docx]

**Supplementary Information**

**MYCL promotes iPSC-like colony formation via MYC Box 0 and 2 domains**

**Authors**

Chiaki Akifuji^1^, Mio Iwasaki^1^, Yuka Kawahara^1^, Chiho Sakurai^1^, Yu-Shen Cheng^1^, Takahiko Imai^1^, and Masato Nakagawa^1^*

**Affiliation**

^1^ Department of Life Science Frontiers, Center for iPS cell Research and Application (CiRA), Kyoto University, Kyoto 606-8507, Japan

*Correspondence: nakagawa@cira.kyoto-u.ac.jp

**Supplementary Figure S1　Comparison of reprogramming conditions.**

1. Schematic representation of HDF reprogramming with Sendai virus (SeV). HDFs were transduced with SeV carrying KLF4-OCT3/4-SOX2 (KOS), KLF4 (K), and c-MYC on day 0. We used an MOI (multiplicity of infection) of 20 for each virus. The reprogramming medium was DMEM+10%FBS or StemFit AK03N with or without bFGF for the first seven days. Reprogramming HDFs were replated at 5 × 10^4^ cells/well of a 6-well plate on day 7. StemFit AK03N with or without bFGF was used as the culture medium after replating. Each condition is labeled as (a), (b), or (c). MC, medium change.
2. Number of colonies obtained by conditions (a), (b), and (c). Mean ± SD values are shown. n=2.
3. Schematic representation of HDF reprogramming with SeV. HDFs were transduced with SeV carrying KLF4-OCT3/4-SOX2 (KOS), KLF4 (K), and c-MYC on day 0. We used an MOI of 14.65 or 4.3 for each virus. StemFit AK03N without bFGF was used as the reprogramming medium for the first seven days. Reprogramming HDFs were replated at 5 × 10^4^ cells on day 7. StemFit AK03N with bFGF was used as the culture medium after replating. Each condition is labeled as (d) or (e). MC, medium change.
4. Number of colonies obtained by conditions (d) and (e). Mean ± SD values are shown. n=2.

**Supplementary Figure S2　Colony images in SeV reprogramming.**

　Representative immunostaining images of reprogramming HDFs stained by anti-TRA-1-60

　antibody (green) and Hoechst (blue) 21 days after the transduction of SeV including c-MYC

　or MYCL in experiment 1 (A) or 2 (B). Scale bars, 300 μm. Ph, phase contrast.

**Supplementary Figure S3　Colony images in EpiP reprogramming.**

　Representative immunostaining images of reprogramming HDFs stained by anti-TRA-1-60

　antibody (green) and Hoechst (blue) 21 days after the transfection of EpiP including

　c-MYC (A) or MYCL (B). The field of view of multiple locations in the same well was

　imaged. Scale bars, 300 μm. Ph, phase contrast.

**Supplementary Figure S4　Cell surface marker expression in EpiP reprogramming.**

Representative flow cytometry images including negative controls for TRA-1-60 and CD13 for HDFs. HDFs without the transduction of any EpiP (EpiP (-)) or with the transduction of EpiP including c-MYC or MYCL. Transfected isotypes showed mixed cells based on staining with isotype controls of TRA-1-60 and CD13 antibodies. EpiP (-), c-MYC, and MYCL were stained by anti-TRA-1-60 and -CD13 antibodies every three days from days 1 to 19 days plus day 21 after the transfection. Numbers indicate the expression percentage of each quadrant.

**Supplementary Figure S5　Preparation of EpiP for domain deletion mutants of c-MYC and MYCL.**

1. Schematic representation of WT and deletion mutants of c-MYC and MYCL proteins. Black boxes show the conserved domains of the MYC proteins. Domain names are indicated above from left to right: MYC Box 0, 1, 2, 3a (c-MYC only), 3b, 4, and bHLHLZ. The numbers on the right indicate amino acid lengths.
2. Alignment of the amino acid sequence between c-MYC and MYCL using MultAlin (http://multalin.toulouse.inra.fr/multalin/). Red shows high consensus amino acids (90%), and blue shows low consensus amino acids (50%). Asterisk (*) shows the conserved tryptophan site: 135 in c-MYC and 96 in MYCL.

**Supplementary Figure S6　EpiP reprogramming with deletion mutants of c-MYC and MYCL.**

　Representative immunostaining images of reprogramming HDFs stained by anti-TRA-1-60

　antibody (green) and Hoechst (blue) 21 days after the transfection of EpiP including

　c-MYC-WT/mutant (A) or MYCL-WT/mutant (B). Scale bars, 300 μm. Ph, phase contrast.

**Supplementary Figure S7　Protein expression of c-MYC-WT/mutants in EpiP reprogramming.**

Representative western blots of reprogramming HDFs transfected with EpiP including c-MYC-WT/mutants on day 2. c-MYC-induced reprogramming HDFs were blotted with anti-c-MYC (upper and middle) and -β-actin (lower) antibodies. MW, molecular weight. Anti-c-MYC antibodies 9E10 and D84C12 recognize the C-terminus (408 – 439 aa) and N-terminus within 30 aa, respectively. 9E10 does not recognize c-MYC-ΔbHLHLZ. D84C12 does not recognize c-MYC-ΔMB0.

**Supplementary Figure S8　Protein expression of MYCL-WT/mutants in EpiP reprogramming.**

Representative western blots of reprogramming HDFs induced with EpiP including MYCL-WT/mutants on day 2. Anti-MYCL and -β-actin antibodies were used. MW, molecular weight.

**Supplementary Figure S9　EpiP reprogramming with deletion mutants of c-MYC and MYCL.**

Representative flow cytometry images. c-MYC and MYCL mean reprogramming HDFs transfected with EpiP including c-MYC-WT/mutants or MYCL-WT/mutants labeled on top, respectively. The transfected cells were stained with anti-TRA-1-60 and -CD13 antibodies on day 16. Numbers indicate the expression percentage of each quadrant.

**Supplementary Figure S10　Comprehensive analysis of protein expressions regulated by c-MYC- and MYCL-WT/ΔMB0 in SeV and EpiP reprogramming.**

1. Venn diagram of up-regulated proteins in c-MYC-reprogramming. The bar graphs below show GO analyses for enriched terms of molecular functions and KEGG pathways in all four groups.
2. Venn diagram of up-regulated proteins in MYCL and c-MYC-ΔMB0 reprogramming. The bar graphs below show GO analyses for enriched terms of molecular functions or KEGG pathways for all four groups.

**Supplementary Figure S11　Phosphorylated proteins during reprogramming by MYCL and c-MYC.**

1. Phosphorylated proteins enriched in MYCL-transduced reprogramming cells by SeV were identified with LC-MS/MS, and the molecular functions were analyzed by GO analysis.
2. Phosphorylated proteins enriched in c-MYC-transduced reprogramming cells by SeV were identified with LC-MS/MS, and the molecular functions were analyzed by GO analysis.

**Supplementary Figure S12　Expression of the MYCL-W96E mutant.**

Representative western blots incubated with anti-MYCL and -β-actin antibodies. Human dermal fibroblasts were transfected with reprogramming factors of EpiP, including MYCL-WT or MYCL-W96E. Reprogramming HDFs were harvested 2 days after the transfection. MW, molecular weight.

**Supplementary Figure S13　Comparison of anti-MYCL antibodies for western blotting.**

Representative western blots incubated with anti-MYCL antibodies, AF4050 and C-20. HDFs were transduced without SeV (SeV (-)) or with SeV, including MYCL-WT (MYCL). Reprogramming HDFs were harvested 2 days after the transfection.

**Supplementary Figure S14　Full-length membrane incubated with anti-c-MYC antibody (9E10), related with Fig. S7.**

**Supplementary Figure S15　Full-length membrane incubated with anti-c-MYC antibody (D84C12), related with Fig. S7.**

**Supplementary Figure S16　Full-length membrane incubated with anti-β-actin antibody, related with Fig. S7.**

**Supplementary Figure S17　Full-length membrane incubated with anti-MYCL antibody, related with Fig. S8.**

**Supplementary Figure S18　Full-length membrane incubated with anti-β-actin antibody, related with Fig. S8.**

**Supplementary Figure S19　Full-length membrane incubated with anti-MYCL antibody, related with Fig. S12.**

**Supplementary Figure S20　Full-length membrane incubated with anti-β-actin antibody, related with Fig. S12.**

**Supplementary Figure S21　Full-length membrane incubated with anti-MYCL (AF4050) antibody, related with Fig. S13.**

**Supplementary Figure S22　Full-length membrane incubated with anti-MYCL (C-20) antibody, related with Fig. S13.**

**Table S1　Primers used for the domain deletion mutants of MYCL and c-MYC.**

The primers for the deletion mutants were designed using the Primer Design tool of the In-Fusion HD Cloning Kit (639650, Takara) and inserted into pENTR1A. pENTR1A replacement with pCXLE was done using the Gateway system.

**Supplementary methods**

**Sample preparation for mass spectrometry analysis.** Cells were lysed with ice-cold lysis buffer with 1% phosphatase inhibitors (P5726-1ML, P0044-1ML, Sigma-Aldrich) and 1% protease inhibitor (P8340-1ML, Sigma-Aldrich). The lysates were subjected to reduction, alkylation, Lys-C/trypsin digestion (enzyme ratio: 1/100, VA1170, V5111, Promega) and desalting, as previously described^1^. The resulting peptides were labeled with isobaric tags for relative and absolute quantification (4352135, iTRAQ, Sciex). Briefly, 120 μg of desalted peptide samples were dried and dissolved in 10 μL of 500 mM triethylammonium bicarbonate (T7408-100ML, Sigma-Aldrich). Approximately 20 μL of iTRAQ reagents were added to 23 μL of ethanol and mixed with the peptide sample. After incubation for one and a half hours at room temperature, 16 μL of 10% TFA (204-02743, Wako) and 400 μL of loading buffer (0.5% trifluoroacetic acid and 4% (v/v) acetonitrile (018-19853, Wako)) were added to quench the reaction. The sample mixture was desalted using StageTip^2^. For the phosphoproteome analysis, 100 μg of iTRAQ labeled peptides were used for the phosphopeptide enrichment by HAMMOC, as previously reported^3^. A total of 8 μg (TT5600) and 500 ng (timsTOF Pro) samples for the whole proteome were subjected to nanoLC-MS/MS analysis. For the phosphoproteome, an enriched total of 150 μg samples was subjected to nanoLC-MS/MS analyses. One-tenth of the iTRAQ-labeled AP samples was mixed and subjected to nanoLC-MS/MS analysis.

**Nano-liquid chromatography coupled to tandem mass spectrometry (nanoLC-MS/MS) analysis.** Samples were subjected to nanoLC-MS/MS using a TripleTOF 5600 System (AB Sciex) equipped with an HTC-PAL autosampler (CTC Analytics) or timsTOF Pro with a nanoElute (Bruker). For the TripleTOF 5600 system, loaded peptides were separated on a monolithic column (4-m length, 100 μm i.d., 5020-4000, GL Science) using a Dionex UltiMate 3000 RSLCnano System. The mobile phases were composed of 0.5% acetic acid with 5% (v/v) DMSO (045-28335, Wako) (solution A) and 0.5% acetic acid in 80% (v/v) acetonitrile with 5% (v/v) DMSO (solution B)^4^. For the whole proteome analysis, a flow rate of 400 nL/min of 5-15% solution B for 205 min, 15-35% solution B for 549 min, 35-40% solution B for 103 min, 40-100% solution B for 5 min, 100% solution B for 118 min, and 5% solution B for 100 min was used (total 1,080 min). For the phosphoproteome analyses, a flow rate of 400 nL/min of 5-10% solution B for 210 min, 10-28% solution B for 375 min, 28-40% solution B for 20 min, 40-100% solution B for 5 min, 100% solution B for 10 min, and 5% solution B for 100 min was used (total 720 min). The coiled monolithic capillary column was connected to a self-pulled emitter (100 μm i.d., 3-5 μm tip) formed with SutterP-2000 (Novato) and a conductive distal coating end applied with Ion Coater Model IB-2 (Eiko Engineering) from which the spray voltage was applied. For the proteome analyses of the affinity precipitation samples, loaded peptides were separated on a self-pulled analytical column (150 mm length, 100 μm i.d.) packed with ReproSil-Pur C18-AQ (3 μm, r13.aq., Dr. Maisch GmbH) at a flow rate of 400 nL/min of 5-40% solution B for 120 min, 40-100% solution B for 5 min, 100% solution B for 5 min, and 5% solution B for 30 min (total 160 min). The applied spray voltage was 2300 V, and the interface heater temperature was 150 ℃. The MS scan range was 300-1500 m/z every 0.25 s, and the MS/MS scan range was 80-1500 m/z every 0.1 s. The maximum number of candidate ions monitored per cycle was 10, and the cycle time was 1.3 s. The resolution of the Q1 scan was UNIT. To minimize repeated scanning, previously scanned ions were excluded for 30 s. Analyses were performed in duplicate, and blank runs were inserted between samples. For the timsTOF Pro system, loaded peptides were separated on an Aurora column (25 cm length, 75 μm i.d., AUR25075C18AC, IonOpticks) using a nanoElute (Bruker). The mobile phases were composed of 0.1% formic acid (066-00461, Wako) (solution A) and 0.1% formic acid in acetonitrile (solution B). For the whole proteome analysis, a flow rate of 400 nL/min of 2-17% solution B for 150 min, 17-25% solution B for 75 min, 25-37% solution B for 25 min, 37-80% solution B for 25 min, and 80% solution B for 10 min was used (total 285 min). The applied spray voltage was 1500 V, and the interface heater temperature was 180 ℃. To obtain MS and MS/MS spectra, the Parallel Accumulation Serial Fragmentation (PASEF) acquisition method (Bruker) was used. Briefly, the trapped ion mobility spectrometry (TIMS) section was operated with a 100-ms ramp time and a scan range of 0.6-1.6 Vs cm-2. One cycle was composed of one MS scan followed by 10 PASEF MS/MS scans, resulting in a total cycle time of 1.16 s. MS and MS/MS spectra were recorded from m/z 100 to 1700. To avoid the selection of singly charged ions, a polygon filter was applied. Analyses were performed in duplicate, and blank runs were inserted between samples.

**Proteome data analysis for protein identification.** For proteome data obtained by the TripleTOF 5600 System, raw data files were analyzed as previously described^5^. For proteome data obtained by the timsTOF Pro system, raw data files were analyzed using Compass DataAnalysis Software (Bruker), and peak lists were generated. The peak lists were analyzed using Mascot v2.5 (Matrix Science) against selected human entries of UniProt/Swiss-Prot release 2020_03 with the carbamidomethylation of cysteine as the fixed modification and the N-terminal iTRAQ, iTRAQ of lysine, protein N-terminal acetylation and methionine oxidation as the variable modification. A precursor mass tolerance of 20 ppm, a fragment ion mass tolerance of 0.1 Da, and strict trypsin and Lys-C specificity, which allowed up to two missed cleavages, were used for the analysis. For the peptide identification, peptides were rejected if any of the following conditions were not satisfied: (a) peptide confidence was below 0.05, (b) the charge state was more than 5, (c) or the peptide length was less than 6 amino acids. Finally, peptides were grouped into protein groups based on previously established rules^6^. False discovery rates (FDRs) were estimated by searching against a decoy sequence database (<1%). For the peptide and protein quantification, the iTRAQ area was normalized by the total area of the whole proteome for each sample. RiMS was used to increase the accuracy of the proteome data obtained by the TripleTOF5600 system^5^. The reliability of the phosphosite localization was assessed using PhosCalc, version 1.2^7^. PTM score-based probabilities were determined using PhosCalc, and phosphosites were grouped into class I (phosphosite probability P > 0.75), class II (0.5 < P ≤ 0.75), or class III (P ≤ 0.5)^8^. Class I phosphosites were accepted automatically as unambiguous sites. The MS/MS data have been deposited to the ProteomeXchange Consortium via jPOSTrepo^9^ (https://repository.jpostdb.org/) with the dataset identifier JPST001236 (PXD027079) for the whole proteome (TT5600 system), JPST001238 (PXD027086) for the whole proteome (timsTOF Pro system), JPST001239 (PXD027083) for the affinity precipitated proteome (TT5600 system), and JPST001237 (PXD027078) for the phosphoproteome (TT5600 system).

**Supplementary information references**

1. Yamana, R. *et al.* Rapid and Deep Profiling of Human Induced Pluripotent Stem Cell Proteome by One-shot NanoLC–MS/MS Analysis with Meter-scale Monolithic Silica Columns. *J. Proteome Res.* **12**, 214–221 (2013).

2. Rappsilber, J., Ishihama, Y. & Mann, M. Stop and Go Extraction Tips for Matrix-Assisted Laser Desorption/Ionization, Nanoelectrospray, and LC/MS Sample Pretreatment in Proteomics. *Anal. Chem.* **75**, 663–670 (2003).

3. Sugiyama, N. *et al.* Phosphopeptide Enrichment by Aliphatic Hydroxy Acid-modified Metal Oxide Chromatography for Nano-LC-MS/MS in Proteomics Applications*S. *Mol. Cell. Proteomics* **6**, 1103–1109 (2007).

4. Hahne, H. *et al.* DMSO enhances electrospray response, boosting sensitivity of proteomic experiments. *Nat. Methods* **10**, 989–991 (2013).

5. Iwasaki, M., Tabata, T., Kawahara, Y., Ishihama, Y. & Nakagawa, M. Removal of Interference MS/MS Spectra for Accurate Quantification in Isobaric Tag-Based Proteomics. *J. Proteome Res.* **18**, 2535–2544 (2019).

6. Nesvizhskii, A. I. & Aebersold, R. Interpretation of Shotgun Proteomic Data. *Mol. Cell. Proteomics* **4**, 1419–1440 (2005).

7. MacLean, D., Burrell, M. A., Studholme, D. J. & Jones, A. M. PhosCalc: A tool for evaluating the sites of peptide phosphorylation from Mass Spectrometer data. *BMC Res. Notes* **1**, 30 (2008).

8. Olsen, J. V. *et al.* Global, In Vivo, and Site-Specific Phosphorylation Dynamics in Signaling Networks. *Cell* **127**, 635–648 (2006).

9. Moriya, Y. *et al.* The jPOST environment: an integrated proteomics data repository and database. *Nucleic Acids Res.* **47**, D1218–D1224 (2019).
