# Supplementary material for: MYCL promotes iPSC-like colony formation via MYC Box 0 and 2 domains
Source: Sci Rep. 2021 Dec 20;11:24254. doi: 10.1038/s41598-021-03260-5 (PMC8688507; doi:10.1038/s41598-021-03260-5)
Supplement: Supplementary file 3 — Supplementary Information 3. [file 41598_2021_3260_MOESM3_ESM.docx]

**【Table S1】**

**Primers used for the domain deletion mutants of MYCL and c-MYC**

| **Primer** | **sequence** |
| --- | --- |
| Mut_MYCL-dMB0_Fw | aaccatgcgctccacggcgcccagc |
| Mut_MYCL-dMB0_Rv | gtggagcgcatggttcctttaaagcc |
| Mut_MYCL-dMB1_Fw | ctccacgccctggggcttgggtccc |
| Mut_MYCL-dMB1_Rv | ccccagggcgtggagcggtagaaatc |
| Mut_MYCL-dMB2_Fw | cgcctccgagagagctgtgagcgac |
| Mut_MYCL-dMB2_Rv | gctctctcggaggcgtagttcctgcc |
| Mut_MYCL-dMB3b_Fw | ggagaatcagtctctgggtattcgg |
| Mut_MYCL-dMB3b_Rv | agagactgattctccgagtcgcttgg |
| Mut_MYCL-dMB4_Fw | atctccatccatcagcaacgttttcc |
| Mut_MYCL-dMB4_Rv | gctttctggaggaaaacgttgctga |
| Mut_MYCL-dbHLHZ_Fw | tgtgacctaaatctagacccagctt |
| Mut_MYCL-dbHLHZ_Rv | tagatttaggtcacatcctcagtatc |
| Mut_c-MYC-dMB0_Fw | accaacaggaactatgaccagcagca |
| Mut_c-MYC-dMB0_Rv | gctctgctgctgctgctggtcatag |
| Mut_c-MYC-dMB1_Fw | gagctgcagcccccggcgagccgccg |
| Mut_c-MYC-dMB1_Rv | gagcccggagcggcggctcgccggg |
| Mut_c-MYC-dMB2_Fw | gagaccttcatcaaaaacgtctcaga |
| Mut_c-MYC-dMB2_Rv | ggccagcttctctgagacgtttttg |
| Mut_c-MYC-dMB3a_Fw | ctgagcgccgccgcctcactcaacga |
| Mut_c-MYC-dMB3a_Rv | cgagctgctgtcgttgagtgaggcg |
| Mut_c-MYC-dMB3b_Fw | ggaggaacaagaagatgagcaggctc |
| Mut_c-MYC-dMB3b_Rv | ccttttgccaggagcctgctcatct |
| Mut_c-MYC-dMB4_Fw | ggaggaacaagaagatgagcaggctc |
| Mut_c-MYC-dMB4_Rv | cttccgagtggagggaggatgtgtg |
| Mut_c-MYC-dbHLHLZ_Fw | gacaccgaggagaatgtctaaatcta |
| Mut_c-MYC-dbHLHLZ_Rv | aagctgggtctagatttagacattc |
